# Catalytic asymmetric tandem Friedel–Crafts alkylation/Michael addition reaction for the synthesis of highly functionalized chromans

**DOI:** 10.3762/bjoc.9.137

**Published:** 2013-06-24

**Authors:** Jiahuan Peng, Da-Ming Du

**Affiliations:** 1 School of Chemical Engineering and Environment, Beijing Institute of Technology, Beijing 100081, China

**Keywords:** asymmetric catalysis, bis(oxazoline), chroman, Friedel–Crafts alkylation, tandem reaction

## Abstract

The enantioselective tandem Friedel–Crafts alkylation/Michael addition reaction of indoles with nitroolefin enoates catalyzed by a diphenylamine-linked bis(oxazoline)-Zn(OTf)_2_ complex was investigated. This tandem reaction afforded functionalized chiral chromans in good yields with moderate to high stereoselectivities (up to 95:5 dr, up to 99% ee).

## Introduction

The development of efficient and convenient methods to access complex compounds with multiple stereogenic centers is one of the significant challenges in organic chemistry. Catalytic asymmetric tandem or cascade reactions are powerful tools to afford complex molecules with multiple stereogenic centers [1–13]. Newly developed tandem/domino reactions are increasingly applied in the synthesis of natural products and other biologically active compounds [14–16].

Dihydrocoumarins, chromans, and chromenes can be found in many natural products and synthetic molecules, and they also possess potentially useful biological properties [17–20]. The benzopyran framework has attracted considerable attention because of the importance of chromans and their biological properties. Numerous synthetic routes have been reported over the past few decades [21–32]. Chiral indolyl(nitro)chromans have been successfully synthesized in our previous study [33]. Good results were obtained in the diastereo- and enantioselective Friedel–Crafts alkylation of indoles with 3-nitro-2*H*-chromenes catalyzed by diphenylamine-linked bis(oxazoline)-Zn(II) complexes. On the other hand, indole and its derivatives are one of the most intensively investigated classical heterocycles owing to their prevalence in bioactive compounds. Indoles have been successfully utilized in asymmetric Friedel–Crafts reactions with nitroolefin and its derivatives in previous reports [34–49]. During the preparation of this manuscript, a similar report on the enantioselective synthesis of highly substituted chromans by a Zinc(II)-catalyzed tandem Friedel–Crafts alkylation/Michael addition reaction has appeared [50]. Herein, we wish to detail our independent research on the asymmetric tandem Friedel–Crafts alkylation/Michael addition reaction of indoles with nitroolefin enoates catalyzed by bis(oxazoline)-Zn(OTf)_2_ complexes, resulting in functionalized chiral chromans (dihydrobenzopyrans) in moderate to high diastereoselectivities (up to 95:5 dr) and enantioselectivities (up to 99% ee).

## Results and Discussion

**Figure 1:**
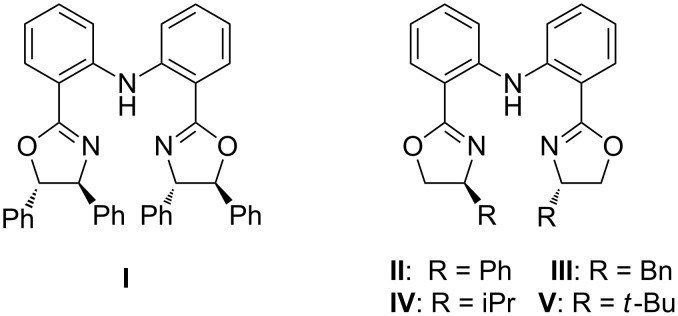
Diphenylamine-linked bis(oxazoline).

Our initial exploratory efforts began with the optimization of the model reaction between nitroolefin enoate **1a** and indole **2a**. First, a series of chiral bis(oxazoline) ligands (**I**–**V**) with Zn(OTf)_2_ as catalysts were investigated in this reaction (Figure 1). The results are summarized in Table 1. It was found that the reaction exhibited good yield and high stereoselectivity with catalysis by the **I**-Zn(OTf)_2_ complex (Table 1, entry 1). Interestingly, with the ligands **III**–**V** (Table 1, entries 3–5), the reaction gave the opposite stereoselectivities probably because of the lower steric hindrance compared with **I** and **II**. Although the opposite diastereomer was obtained in good yield and stereoselectivity with the use of ligand **IV**, ligand **I** was the preferred one according to the results.

**Table 1:** Effect of ligands on the asymmetric tandem Friedel–Crafts alkylation/Michael addition reaction.

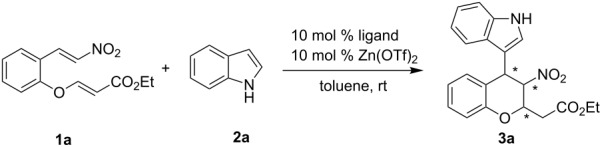				

entry^a^	ligand	yield (%)^b^	dr^c^	ee (%)^c,d^

1	**I**	48	96:4	83/–
2	**II**	59	88:12	55/–
3	**III**	43	33:67	–/68
4	**IV**	56	17:83	–/73
5	**V**	35	29:71	–/53

^a^Reaction conditions: nitroolefin enoate **1a** (0.1 mmol) with indole **2a** (0.1 mmol) in 1.5 mL of toluene catalyzed by 10 mol % ligand-Zn(OTf)_2_ complex for 24 h at room temperature. ^b^Isolated yields by column chromatography. ^c^Determined by HPLC on Daicel Chiralpak IA column (*n*-hexane/2-propanol 85:15, 0.5 mL/min). ^d^ee for the major diastereomer.

In order to increase the yield and stereoselectivity of the desired product, further screening of reaction parameters such as the ratio of substrates and temperature were investigated. When 1.5 equiv of indole was used in the reaction, a significant improvement of the yield was realized (Table 2, entry 2). The enantioselectivity of the product **3a** was improved slightly by lowering the reaction temperature (Table 2, entries 7 and 8). Raising the temperature to 50 °C led to a decrease of yield, diastereoselectivity and enantioselectivity. After a brief screening of the solvent, toluene was found to be the best choice.

**Table 2:** Optimization of reaction conditions.

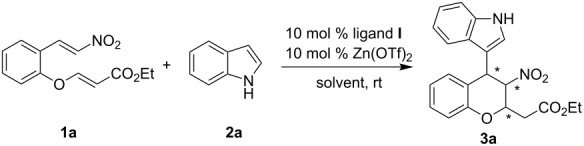				

entry^a^	solvent	yield (%)^b^	dr^c^	ee (%)^c^

1^d^	toluene	48	96:4	83
2	toluene	58	96:4	84
3	xylene	49	94:6	82
4	α,α,α-trifluorotoluene	56	95:5	54
5	CH_2_ClCH_2_Cl	37	89:11	69
6	THF	trace	–	–
7^e^	toluene	55	97:3	87
8^f^	toluene	58	96:4	87
9^g^	toluene	45	93:7	74

^a^Reaction conditions: nitroolefin enoate **1a** (0.1 mmol) with indole **2a** (0.15 mmol) in 1.5 mL of toluene catalyzed by 10 mol % ligand **I**-Zn(OTf)_2_ complex for 24 h at room temperature. ^b^Isolated yields by column chromatography. ^c^Determined by HPLC on Daicel Chiralpak IA column (*n*-hexane/2-propanol 85:15, 0.5 mL/min). ^d^1 equiv of indole **2a** (0.1 mmol) was used. ^e^The reaction was performed at 0 °C for 48 h. ^f^The reaction was performed at –10 °C for 48 h. ^g^The reaction was performed at 50 °C for 24 h.

Different additives (10 mol %) were then tested in the presence of 10 mol % of **I**-Zn(OTf)_2_ complex. Remarkably, a substantial improvement of the yield was realized when Et_3_N was added; however, the diastereo- and enantioselectivity dropped significantly (Table 3, entry 2). With both NH(C_2_H_5_)_2_ and TMEDA, no desired product was observed (Table 3, entries 3 and 4). DABCO led to no significant increase in yield and stereoselectivity (Table 3, entry 5). A substantial increase in enantioselectivity was observed with the use of LiO*t*-Bu, but the yield remained moderate (Table 3, entry 7). Among the additives probed, the best results (73% yield, 96:4 dr and 89% ee) were achieved when NaO*t-*Bu was used as an additive in the reaction (Table 3, entry 8).

**Table 3:** Effect of additives on asymmetric tandem Friedel–Crafts alkylation/Michael addition reaction.

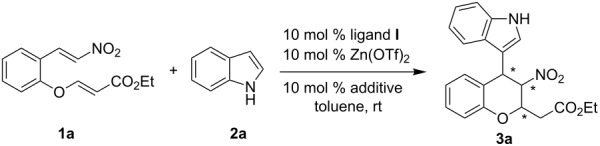				

entry^a^	additive	yield (%)^b^	dr^c^	ee (%)^c^

1	–	58	96:4	83
2	Et_3_N	76	79:21	63
3	NH(C_2_H_5_)_2_	0	–	–
4	TMEDA	0	–	–
5	DABCO	41	93:7	82
6	CsCO_3_	62	98:2	80
7	LiO*t*-Bu	47	95:5	92
8	NaO*t*-Bu	73	96:4	89
9	KO*t*-Bu	62	96:4	84

^a^Reaction conditions: nitroolefin enoate **1a** (0.1 mmol) with indole **2a** (0.15 mmol) in 1.5 mL of toluene catalyzed by 10 mol % ligand **I**-Zn(OTf)_2_ complex with 10 mol % additive for 24 h at room temperature. ^b^Isolated yields by column chromatography. ^c^Determined by HPLC on Daicel Chiralpak IA column (*n*-hexane/2-propanol 85:15, 0.5 mL/min).

**Figure 2:**
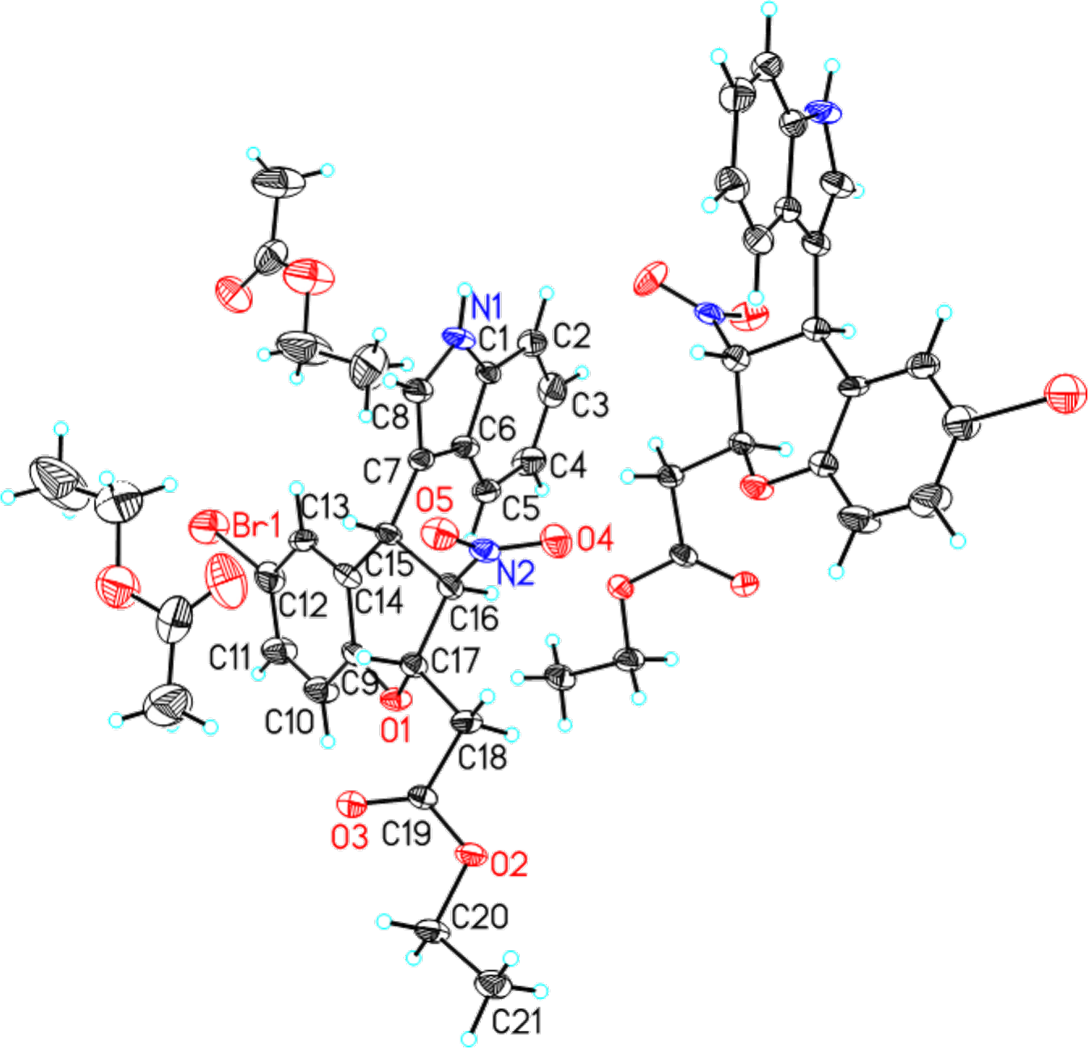
X-ray crystal structure of the major diastereomer of **3g** (one symmetric molecule and two solvent molecules are not labeled for clarity).

After optimization of the reaction conditions, the substrate scope of the enantioselective Friedel–Crafts alkylation/Michael addition of nitroolefin enoates **1** with indoles **2** was explored. The results are summarized in Table 4 (see Supporting Information File 1 for full experimental data). Both electron withdrawing and electron-rich substituents in the 5-position of the indole caused moderate decrease in enantioselectivity and diastereoselectivity (Table 4, entries 2–4). Nitroolefin enoates **1b** or **1c** with chlorine or bromine on the aromatic ring reacted smoothly to afford products **3f** or **3g** with good yields and stereoselectivities (Table 4, entries 6 and 7). We found that incorporation of an electron-donating methoxy group on the aromatic ring of the nitroolefin enaote had a significant effect on both yield and stereoselectivity. In the case of methoxy-substituted nitroolefin enoates **1e** (Table 4, entry 9), the yield of the product **3i** decreased to 12% and the enantioselectivity decreased to 24% even though the reaction time was prolonged to 96 h. A similar result was observed in the case of product **3j** (Table 4, entry 10). A steric effect was also observed in this reaction. When the substrate **1g** bearing two sterically hindered bromine atoms on the phenyl ring was used, the yield and stereoselectivity of the desired product **3k** decreased significantly (Table 4, entry 11). The configuration of the major diastereomer of **3g** was determined to be C15(*S*), C16(*R*), C17(*S*) (Figure 2), and those of other products were assigned by analogy [51].

**Table 4:** Asymmetric tandem Friedel–Crafts alkylation/Michael addition reaction of nitroolefin enoates with indoles.

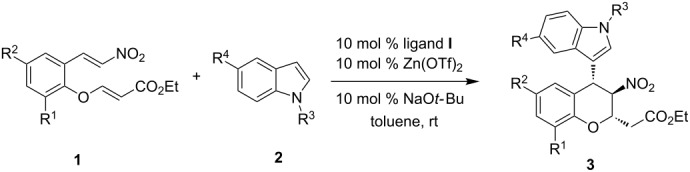								

entry^a^	R^1^	R^2^	R^3^	R^4^	product	yield (%)^b^	dr^c^	ee (%)^c,d^

1^e^	H	H	H	H	**3a**	73	96:4	89
2	H	H	H	CH_3_	**3b**	69	90:10	80
3	H	H	H	OCH_3_	**3c**	76	93:7^f^	72
4	H	H	H	Cl	**3d**	49	82:18^f^	62
5	H	H	CH_3_	H	**3e**	60	88:12^f^	67
6	H	Cl	H	H	**3f**	65	95:5	91
7	H	Br	H	H	**3g**	66	93:7	87
8	H	NO_2_	H	H	**3h**	56	85:15^f^	80
9^g^	OCH_3_	H	H	H	**3i**	12	–^h^	24
10^g^	OC_2_H_5_	H	H	H	**3j**	47	85:15^f^	31
11	Br	Br	H	H	**3k**	21	72:28	57

^a^Reaction conditions: nitroolefin enoates **1** (0.2 mmol) with indoles **2** (0.3 mmol) in 3 mL of toluene catalyzed by 10 mol % ligand-Zn(OTf)_2_ complex with 10 mol % NaO*t*-Bu for 24 h at room temperature. ^b^Isolated yields by column chromatography. ^c^Determined by HPLC. ^d^ee for the major diastereomer. ^e^Nitroolefin enoate **1a** (0.1 mmol) with indole **2a** (0.15 mmol). ^f^Determined by the weight ratio of isolated diastereomers. ^g^The reaction time was 96 h. ^h^The minor diastereomer was not detected.

**Scheme 1:**
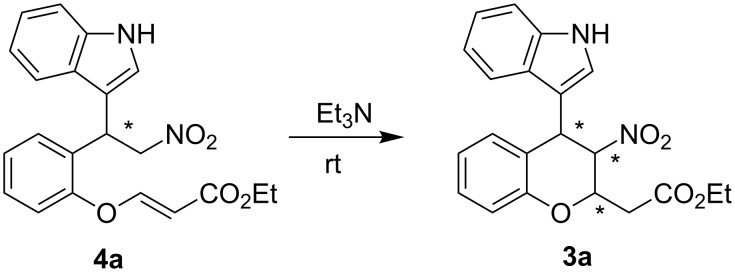
The transformation of Friedel–Crafts alkylation product **4a** to cycloadduct **3a**.

The results of the substrate scope are unsatisfactory. The yields of the desired products **3** were affected by the side products **4**, which were the Friedel–Crafts alkylation products of nitroolefin enoates and indoles. Fortunately, it was found that the model Friedel–Crafts alkylation product **4a**, which was isolated, could be transformed to the desired cycloadduct **3a** with high stereoselectivity in the presence of 5 equiv of Et_3_N at room temperature (Scheme 1). With the success of this model reaction, the substrate scope in Table 4 was reinvestigated. The corresponding reactions proceeded smoothly to afford desired products **3** and side products **4** at –10 °C. After the nitroolefin enoates were consumed, 5 equiv of Et_3_N was added to the reaction at room temperature. The new results are summarized in Table 5. All reactions proceeded smoothly affording desired products **3** with good to excellent yields. However, the diastereoselectivities of the products **3** decreased in all cases. Excellent enantioselectivities were observed with indoles bearing electron-rich substituents (Table 5, entries 2 and 3). Both the electron-withdrawing and electron-rich substituents on the aromatic ring of nitroolefin enoates afforded **3** in low diastereoselectivities (Table 5, entries 6–10). Good to excellent enantioselectivities were observed in these cases. **3k** was obtained in moderate stereoselectivity (Table 5, entry 11).

**Table 5:** Asymmetric tandem Friedel–Crafts alkylation/Michael addition reaction of nitroolefin enoates with indoles.

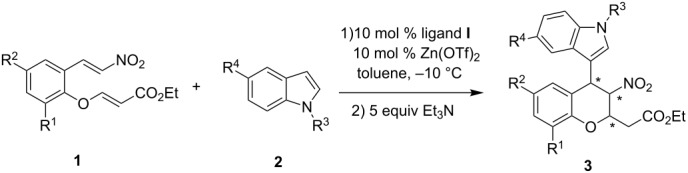								

entry^a^	R^1^	R^2^	R^3^	R^4^	product	Yield (%)^b^	dr^c^	ee (%)^c,d^

1	H	H	H	H	**3a**	88	92:8	92/88
2	H	H	H	CH_3_	**3b**	88	44:56^e^	62/98
3	H	H	H	OCH_3_	**3c**	96	28:72^e^	73/99
4	H	H	H	Cl	**3d**	58	76:24	82/53
5	H	H	CH_3_	H	**3e**	89	82:18^e^	65/79
6	H	Cl	H	H	**3f**	86	74:26	95/95
7	H	Br	H	H	**3g**	100	60:40	91/93
8	H	NO_2_	H	H	**3h**	89	49:51	95/95
9	OCH_3_	H	H	H	**3i**	85	58:42^e^	39/83
10	OC_2_H_5_	H	H	H	**3j**	94	54:46^e^	30/97
11	Br	Br	H	H	**3k**	75	38:62	53/90

^a^Reaction conditions: nitroolefin enoate **1a** (0.2 mmol) with indole **2a** (0.3 mmol) in 3 mL of toluene catalyzed by 10 mol % ligand-Zn(OTf)_2_ complex for 72 h at –10 °C. Subsequently, 5 equiv of Et_3_N was added. ^b^Isolated yields by column chromatography. ^c^Determined by HPLC. ^d^ee for both diastereomers. ^e^Determined by the weight ratio of isolated diastereomers.

## Conclusion

In conclusion, we have developed a convenient catalytic asymmetric tandem Friedel–Crafts alkylation/Michael addition reaction of nitroolefin enoates **1** with indoles **2** catalyzed by a tridentate bis(oxazoline) **I**-Zn(OTf)_2_ complex. Moderate to high stereoselectivities (up to 95:5 dr, up to 99% ee) and good to excellent yields of the functionalized chiral chromans were obtained. Further applications of these catalysts in other reactions are underway in our laboratory.

## Experimental

General procedure **A** for the catalytic asymmetric tandem Friedel–Crafts alkylation/Michael addition reaction of indoles with nitroolefin enoates: Into a dried Schlenk tube were added Zn(OTf)_2_ (7.3 mg, 0.02 mmol), ligand **I** (12.2 mg, 0.02 mmol) and NaO*t*-Bu (1.9 mg, 0.02 mmol) under argon followed by the addition of toluene (3 mL). The solution was stirred at room temperature for 0.5 h, and then nitroolefin enoate **1** (0.2 mmol) was added. The mixture was stirred for 10 min then the indole **2** (0.3 mmol) was added. After stirring for 48 h at room temperature, the solvent was removed under vacuum. Purification by column chromatography afforded the desired products **3**.

General procedure **B** for the catalytic asymmetric tandem Friedel–Crafts alkylation/Michael addition reaction of indoles with nitroolefin enoates: Into a dried Schlenk tube were added Zn(OTf)_2_ (7.3 mg, 0.02 mmol) and ligand **I** (12.2 mg, 0.02 mmol) under argon followed by the addition of toluene (3 mL). The solution was stirred at room temperature for 0.5 h and then nitroolefin enoate **1** (0.2 mmol) was added. The mixture was stirred for 10 min then the indole **2** (0.3 mmol) was added. After stirring for 72 h at –10 °C, Et_3_N (100 mg, 1 mmol) was added, and the mixture was stirred for another 24 h at room temperature. The solvent was removed under vacuum. Purification by column chromatography afforded the desired products **3**.

## Supporting Information

File 1 Characterization data, copies of NMR spectra and HPLC chromatographs of products **3**.

File 2 Crystallographic data of compound **3g**.
