# Supplementary material for: Catalytic asymmetric tandem Friedel–Crafts alkylation/Michael addition reaction for the synthesis of highly functionalized chromans
Source: Beilstein J Org Chem. 2013 Jun 24;9:1210–6. doi: 10.3762/bjoc.9.137 (PMC3701415; doi:10.3762/bjoc.9.137)
Supplement: File 1 — Characterization data, copies of NMR spectra and HPLC chromatographs of products 3. [file Beilstein_J_Org_Chem-09-1210-s001.pdf]

Supporting Information

for

Catalytic asymmetric tandem Friedel–Crafts alkylation/Michael addition reaction for the synthesis of highly functionalized chromans

Jiahuan Peng and Da-Ming Du*

Address: School of Chemical Engineering and Environment, Beijing Institute of Technology, Beijing 100081, China

Email: Da-Ming Du*- dudm@bit.edu.cn

* Corresponding author

Characterization data, copies of NMR spectra and HPLC chromatographs of products 3.

General Methods: Commercially available compounds were used without further purification. Solvents were dried according to standard procedures. Column chromatography was performed using silica gel (200–300 mesh). Melting points were determined on a XT–4 melting point apparatus without correction. The ^{1}H NMR spectra were measured on Varian Mercury-plus 400 MHz spectrometer. Chemical shifts were recorded in δ (ppm) relative to tetramethylsilane (TMS) as the internal standard. ^{13}C NMR spectra were measured at 100 MHz. Chemical shifts are reported in ppm with the solvent resonance as the internal standard. Infrared spectra were obtained with a Perkin Elmer Spectrum One spectrometer. ESI-HRMS spectra were measured with a Bruker APEX IV Fourier-Transform mass spectrometer. Optical rotations were measured with a WZZ–3 polarimeter. Enantiomeric excesses were determined by chiral HPLC using an Agilent 1200 LC instrument with Daicel Chiralpak IA, IB or ADH column.

Materials: Chiral bis(oxazoline) **I**−**X** [1] and nitroalkene enoates **1** [2] were prepared according to the reported procedures. The racemic reference compounds of **3** were synthesized by the direct treatment of nitroalkene enoates **1** and indoles **2** in the presence of 10 mol% $Zn(OTf)_2$.

General Procedure (A) for the catalytic asymmetric tandem Friedel–Crafts alkylation/Michael addition reaction of indoles with nitroolefin enoates: To a dried Schlenk tube were added $Zn(OTf)_2$ (7.3 mg, 0.02 mmol), ligand **I** (12.2 mg, 0.02 mmol) and NaO*t*Bu (1.9 mg, 0.02 mmol) under argon followed by the addition of toluene (3 mL). The solution was stirred at room temperature for 0.5 h and then nitroolefin enoate **1** (0.2 mmol) was added. The mixture was stirred for 10 min then the indole **2** (0.3 mmol) was added. After stirring for 48 h at room temperature, the solvent was removed under vacuum. Purification by column chromatography afforded the desired product **3**.

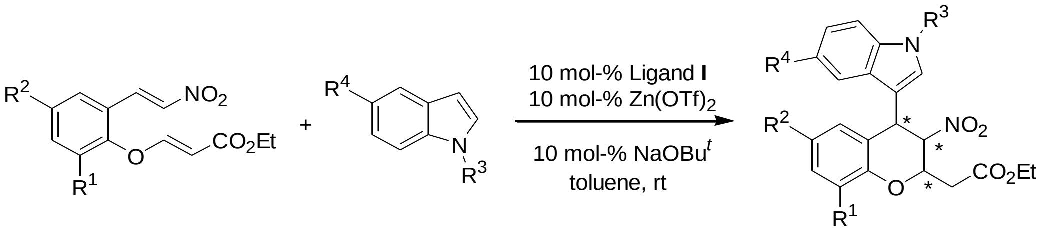

1 **2** **3**

Ethyl 2-(4-(1*H*-indol-3-yl)-3-nitro-chroman-2-yl)acetate (3a): Compound **3a** (the mixture of the major and minor diastereomer) was obtained according to the general procedure **A** (27.7 mg, 73% yield). It was analyzed to determine the diastereoselectivity and enantioselectivity of the reaction (96:4 dr, 89% ee for the major diastereomer) by HPLC (Daicel Chiralpak IA column, *n*-hexane/2-propanol 85:15, flow rate 0.5 mL·min^{-1}, detection at 254 nm), major diastereomer: t_{major} = 19.9 min, t_{minor} = 18.7 min; minor diastereomer: t_R = 17.3 min. The major diastereomer was purified by flash chromatography and obtained as a white solid, mp 163–168 °C. $[\alpha]_D^{25}$ = +76.5 (*c* 1.51 g/100 mL, CH_2Cl_2). ^{1}H NMR (400 MHz, $CDCl_3$) δ 8.17 (s, 1H, NH), 7.36 (d, *J* = 8.0 Hz, 1H, ArH), 7.17–7.14 (m, 3H, ArH), 6.95–6.93

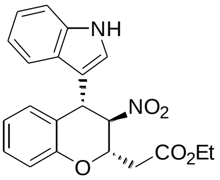

(m, 4H, ArH), 6.81 (t, J = 7.2 Hz, 1H, ArH), 5.23 (t, J = 10.4 Hz, 1H, CH), 5.09 (d, J = 10.4 Hz, 1H, CH), 4.94–4.88 (m, 1H, CH), 4.23 (q, J = 7.0 Hz, 2H, CH_2), 2.84–2.71 (m, 2H, CH_2), 1.31–1.26 (m, 3H, CH_3) ppm. ^{13}C NMR (100 MHz, $CDCl_3$) δ169.1, 152.6, 136.8, 129.5, 128.4, 124.8, 124.5, 122.5, 122.1, 121.9, 119.9, 119.2, 116.6, 111.9, 111.6, 88.2, 73.2, 61.2, 40.5, 37.2, 14.1 ppm. IR (KBr): ν 3416, 3061, 2983, 2908, 1737, 1585, 1552, 1485, 1456, 1365, 1281, 1230, 1189, 1085, 1055, 745 cm^{-1}. HRMS (ESI): m/z calcd. for $C_{21}H_{20}N_2NaO_5$ $[M + Na]^+$ 403.12644, found: 403.12618.

Ethyl 2-(4-(5-methyl-1*H*-indol-3-yl)-3-nitro-chroman-2-yl)acetate (3b): Compound **3b** (the mixture of the major and minor diastereomer) was obtained according to the general procedure **A** (54.2 mg, 69% yield). It was analyzed to determine the diastereoselectivity and enantioselectivity of the reaction (90:10 dr, 80% ee for the major diastereomer) by HPLC (Daicel Chiralpak IB column, n-hexane/2-propanol = 90:10, flow rate 0.5 mL·min^{-1}, detection at 254 nm), major diastereomer: t_{major} = 26.4 min, t_{minor} = 28.4 min; minor diastereomer: t_R = 22.7 min. The major diastereomer was purified by flash chromatography and obtained as a yellow oil. $[\alpha]_D^{25}$: +98.7 (c 3.83 g/100 mL, CH_2Cl_2). ^{1}H NMR (400 MHz, $CDCl_3$) δ 8.08 (s, 1H, NH), 7.24 (d, J = 7.6 Hz, 1H, ArH), 7.17 (t, J = 7.4 Hz, 1H, ArH), 7.08 (d, J = 2.4 Hz, 1H, ArH), 6.99–6.93 (m, 3H, ArH), 6.81 (t, J = 7.2 Hz, 1H, ArH), 6.73 (s, 1H, ArH), 5.22 (t, J = 10.0 Hz, 1H, CH), 5.06 (d, J = 10.4 Hz, 1H, CH), 4.94–4.88 (m, 1H, CH), 4.23 (q, J = 7.2 Hz, 2H, CH_2), 2.86–2.71 (m, 2H, CH_2), 2.29 (s, 3H, CH_3), 1.29 (t, J = 7.2 Hz, 3H, CH_3) ppm. ^{13}C NMR (100 MHz, $CDCl_3$) δ 169.1, 152.6, 135.2, 129.5, 129.2, 128.4, 125.2, 124.5, 124.2, 122.2, 122.1, 118.7, 116.5, 111.4, 111.2, 88.2, 73.3, 61.2, 40.4, 37.3, 21.4, 14.1 ppm. IR (KBr): ν 3415, 2981, 2925, 1736, 1584, 1552, 1485, 1455, 1367, 1280, 1229, 1197, 1054, 798, 759 cm^{-1}. HRMS (ESI): calcd. for $C_{22}H_{23}N_2O_5$ $[M + H]^+$ 395.16015, found: 395.16069; calcd. for $C_{22}H_{22}N_2NaO_5$ $[M + Na]^+$ 417.14209, found: 417.14216.

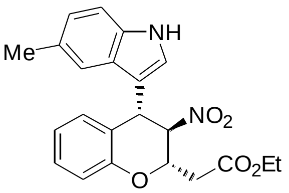

Ethyl 2-(4-(5-methoxy-1*H*-indol-3-yl)-3-nitro-chroman-2-yl)acetate (3c): Compound **3c** (the mixture of the major and minor diastereomer) was obtained according to the general procedure **A** (62.0 mg, 76% yield). It was analyzed to determine the diastereoselectivity of the reaction by the ratio of weight (93:7 dr). The enantioselectivity for major diastereomer (72% ee) was determined by HPLC (Daicel Chiralpak IA column, n-hexane/2-propanol= 90:10, flow rate 0.5 mL·min^{-1}, detection at 254 nm), t_{major} = 40.1 min, t_{minor} = 38.2 min. The major diastereomer was purified by flash chromatography and obtained as a yellow oil. $[\alpha]_D^{25}$: +58.7 (c 2.35 g/100 mL, CH_2Cl_2). ^{1}H NMR (400 MHz, $CDCl_3$) δ 8.13 (s, 1H, NH), 7.23 (d, J = 8.8 Hz, 1H, ArH), 7.16 (t, J = 7.4 Hz, 1H, ArH), 7.08 (d, J = 2.4 Hz, 1H, ArH), 6.97–6.94 (m, 2H, ArH), 6.84–6.80 (m, 2H, ArH), 6.35 (d, J = 2.0 Hz, 1H, ArH), 5.18 (t, J = 10.2 Hz, 1H, CH), 5.07 (d, J = 10.8 Hz, 1H, CH), 4.93–4.88 (m, 1H, CH), 4.23 (q, J = 7.2 Hz, 2H, CH_2), 3.62 (s, 3H, OCH_3), 2.85–2.71 (m, 2H, CH_2), 1.29 (t, J = 7.2 Hz, 3H, CH_3) ppm. ^{13}C NMR (100 MHz, $CDCl_3$) δ 169.1, 153.9, 152.6, 131.9, 129.5, 128.4, 125.5, 125.0, 122.1, 121.9, 116.5, 112.3, 111.6, 101.2, 88.2, 73.2, 61.2, 55.6, 40.3, 37.2, 14.1 ppm. IR (KBr): ν 3417, 2985, 2938, 1736, 1625,

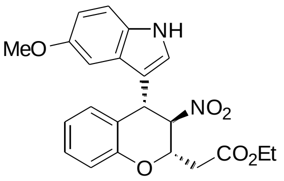

1584, 1553, 1486, 1456, 1366, 1302, 1280, 1222, 1175, 1055, 1029, 925, 804, 760 cm^{-1}. HRMS (ESI): calcd. for $C_{22}H_{23}N_2O_6$ $[M + H]^+$ 411.15506, found: 411.15478.

Ethyl 2-(4-(5-chloro-1*H*-indol-3-yl)-3-nitro-chroman-2-yl)acetate (3d): Compound **3d** (the mixture of the major and minor diastereomer) was obtained according to the general procedure **A** (40.6 mg, 49% yield). It was analyzed to determine the diastereoselectivity of the

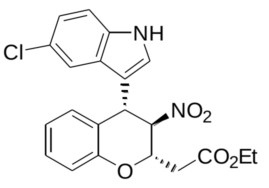

reaction by the ratio of weight (82:18 dr). The enantioselectivity for the major diastereomer (62% ee) was determined by HPLC (Daicel Chiralpak IB column, *n*-hexane/2-propanol 90:10, flow rate 0.5 mL·min^{-1}, detection at 254 nm), t_{major} = 36.5 min, t_{minor} = 41.2 min. The major diastereomer was purified by flash chromatography and obtained as a yellow oil. $[\alpha]_D^{25}$: +39.9 (*c* 1.47 g/100 mL, CH_2Cl_2). ^1H NMR (400 MHz, $CDCl_3$) δ 8.25 (s, 1H, NH), 7.29 (d, *J* = 8.4 Hz, 1H, ArH), 7.22–7.11 (m, 3H, ArH), 6.97 (d, *J* = 8.4 Hz, 1H, ArH), 6.90–6.82 (m, 3H, ArH), 5.21–5.16 (m, 1H, CH), 5.06 (d, *J* = 10.4 Hz, 1H, CH), 4.93–4.88 (m, 1H, CH), 4.24 (q, *J* = 7.2 Hz, 2H, CH_2), 2.87–2.72 (m, 2H, CH_2), 1.31 (t, *J* = 7.2 Hz, 3H, CH_3) ppm. ^{13}C NMR (100 MHz, $CDCl_3$) δ 169.0, 152.6, 135.2, 129.2, 128.7, 125.9, 125.8, 125.6, 123.0, 122.3, 121.3, 118.6, 116.7, 112.7, 111.6, 88.1, 73.2, 61.3, 40.4, 37.2, 14.1 ppm. IR (KBr): ν 3423, 2982, 1729, 1584, 1552, 1484， 1456, 1366, 1279, 1228, 1055, 893, 798, 757 cm^{-1}. HRMS (ESI): calcd. for $C_{21}H_{20}ClN_2O_5$ $[M + H]^+$ 415.10553, found: 415.10519; calcd. for $C_{21}H_{23}ClN_3O_5$ $[M + NH_4]^+$ 432.13207, found: 432.13171.

Ethyl 2-(4-(1-methyl-1*H*-indol-3-yl)-3-nitro-chroman-2-yl)acetate (3e) [3]: Compound **3e**

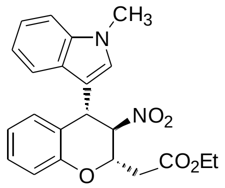

(the mixture of the major and minor diastereomer) was obtained according to the general procedure **A** (47.3 mg, 60% yield). It was analyzed to determine the diastereoselectivity of the reaction by the ratio of weight (88:12 dr). The enantioselectivity for major diastereomer (67% ee) was determined by HPLC (Daicel Chiralpak IA column, *n*-hexane/2-propanol 95:5, flow rate 0.5 mL·min^{-1}, detection at 254 nm), t_{major} = 29.3 min, t_{minor} = 22.4 min. The major diastereomer was purified by flash chromatography and obtained as a yellow oil. $[\alpha]_D^{25}$: +69.7 (*c* 1.56 g/100 mL, CH_2Cl_2). ^1H NMR (400 MHz, $CDCl_3$) δ 7.30 (d, *J* = 8.4 Hz, 1H, ArH), 7.24–7.14 (m, 2H, ArH), 7.01–6.94 (m, 5H, ArH), 6.82–6.78 (m, 1H, ArH), 5.23–5.17 (m, 1H, CH), 5.07 (d, *J* = 10.4 Hz, 1H, CH), 4.92–4.86 (m, 1H, CH), 4.22 (q, *J* = 7.2 Hz, 2H, CH_2), 3.74 (s, 3H, NCH_3), 2.83–2.69 (m, 2H, CH_2), 1.29 (t, *J* = 7.2 Hz, 3H, CH_3) ppm. ^{13}C NMR (100 MHz, $CDCl_3$) δ 169.0, 152.6, 137.6, 129.5, 128.9, 128.3, 125.5, 122.2, 122.1, 122.0, 119.4, 119.3, 116.5, 110.3, 109.6, 88.4, 73.2, 61.1, 40.3, 37.2, 32.8, 14.1 ppm. IR (KBr): ν 3059, 2982, 2936, 1740, 1584, 1552, 1485, 1456, 1369, 1332, 1281, 1231, 1212, 1187, 1054, 921, 760, 742 cm^{-1}. HRMS (ESI): calcd. for $C_{22}H_{23}N_2O_5$ $[M + H]^+$ 395.16015, found: 395.15976; calcd. for $C_{22}H_{22}N_2NaO_5$ $[M + Na]^+$ 417.14209, found: 417.14127. The preparation of chiral **3e** with a different configuration was reported in the literature[3], $[\alpha]_D^{22}$ –66.63 (*c* 1.00, $CHCl_3$), 83% ee (>95:5 dr).

Ethyl 2-(6-chloro-4-(1*H*-indol-3-yl)-3-nitro-chroman-2-yl)acetate (3f): Compound **3f** (the mixture of the major and minor diastereomer) was obtained according to the general procedure **A** (53.8 mg, 65% yield). It was analyzed to determine the diastereoselectivity and enantioselectivity of the reaction (95:5 dr, 91% ee for the major diastereomer) by HPLC (Daicel Chiralpak IA column, *n*-hexane/2-propanol 90:10, flow rate 0.5 mL·min^{-1}, detection at 254 nm), major diastereomer: t_{major} = 32.7 min, t_{minor} = 28.4 min; minor diastereomer: t_{major} = 22.9 min, t_{minor} = 20.5 min. The major diastereomer was purified by flash chromatography and obtained as a yellow oil. $[\alpha]_D^{25}$: +12.6 (*c* 2.34 g/100 mL, CH_2Cl_2). ^{1}H NMR (400 MHz, $CDCl_3$) δ 8.24 (s, 1H, NH), 7.36 (d, *J* = 8.4 Hz, 1H, ArH), 7.20–7.11 (m, 3H, ArH), 6.99 (d, *J* = 4.0 Hz, 2H, ArH), 6.90 (d, *J* = 8.0 Hz, 2H, ArH), 5.24 (t, *J* = 10.2 Hz, 1H, CH), 5.04 (d, *J* = 10.4 Hz, 1H, CH), 4.91–4.86 (m, 1H, CH), 4.22 (q, *J* = 7.2 Hz, 2H, CH_2), 2.84–2.71 (m, 2H, CH_2), 1.28 (t, *J* = 7.2 Hz, 3H, CH_3) ppm. ^{13}C NMR (100 MHz, $CDCl_3$) δ 168.9, 151.2, 136.8, 128.9, 128.6, 127.1, 124.6, 124.5, 123.8, 122.6, 120.1, 118.9, 118.0, 111.7, 111.0, 87.6, 73.3, 61.3, 40.2, 37.0, 14.1 ppm. IR (KBr): *ν* 3417, 3060, 2983, 1736, 1554, 1479, 1458, 1365, 1263, 1228, 1186, 1096, 1147, 898, 823, 744 cm^{-1}. HRMS (ESI): calcd. for $C_{21}H_{20}ClN_2O_5$ $[M + H]^+$ 415.10553, found: 415.10520; calcd. for $C_{21}H_{23}ClN_3O_5$ $[M + NH_4]^+$ 432.13207, found: 432.13170.

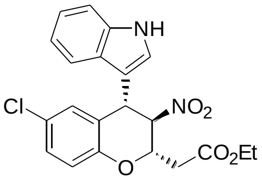

Ethyl 2-(6-bromo-4-(1*H*-indol-3-yl)-3-nitro-chroman-2-yl)acetate (3g): Compound **3g** (the mixture of the major and minor diastereomer) was obtained according to the general procedure **A** (60.9 mg, 66% yield). It was analyzed to determine the diastereoselectivity and enantioselectivity of the reaction (93:7 dr, 87% ee for the major diastereomer) by HPLC (Daicel Chiralpak IB column, *n*-hexane/2-propanol 90:10, flow rate 0.5 mL·min^{-1}, detection at 254 nm), major diastereomer: t_{major} = 35.7 min, t_{minor} = 38.8 min; minor diastereomer: t_{major} = 32.5 min, t_{minor} = 25.4 min. The major diastereomer was purified by flash chromatography and obtained as a yellow solid; mp 156–159 °C. $[\alpha]_D^{25}$: +6.3 (*c* 1.88 g/100 mL, CH_2Cl_2). ^{1}H NMR (400 MHz, $CDCl_3$) δ 8.22 (s, 1H, NH), 7.37 (d, *J* = 8.4 Hz, 1H, ArH), 7.28–7.25 (m, 1H, ArH), 7.21–7.17 (m, 1H, ArH), 7.13 (d, *J* = 2.4 Hz, 1H, ArH), 7.06–7.00 (m, 3H, ArH), 6.85 (d, *J* = 8.8 Hz, 1H, ArH), 5.23 (t, *J* = 10.2 Hz, 1H, CH), 5.05 (d, *J* = 10.8 Hz, 1H, CH), 4.91–4.86 (m, 1H, CH), 4.22 (q, *J* = 7.0 Hz, 2H, CH_2), 2.83–2.70 (m, 2H, CH_2), 1.28 (t, *J* = 7.0 Hz, 3H, CH_3) ppm. ^{13}C NMR (100 MHz, $CDCl_3$) δ 168.9, 151.8, 136.8, 131.8, 131.5, 124.6, 124.5, 124.3, 122.6, 120.2, 118.9, 118.5, 114.5, 111.7, 111.1, 87.6, 73.4, 61.3, 40.1, 37.0, 14.1 ppm. IR (KBr): *ν* 3418, 2982, 1736, 1553, 1476, 1458, 1365, 1265, 1229, 1184, 1047, 820, 744 cm^{-1}. HRMS (ESI): calcd. for $C_{21}H_{20}BrN_2O_5$ $[M + H]^+$ 459.05501, found: 459.05524; calcd. for $C_{21}H_{19}BrN_2NaO_5$ $[M + Na]^+$ 481.03696, found: 481.03643.

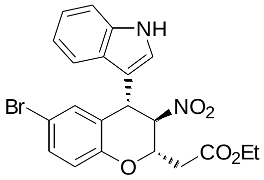

Ethyl 2-(4-(1*H*-indol-3-yl)-3,6-dinitro-2*H*-chroman-2-yl)acetate (3h): Compound **3h** (the mixture of the major and minor diastereomer) was obtained according to the general procedure **A** (47.8 mg, 56% yield). It was analyzed to determine the diastereoselectivity of the reaction by the ratio of weight (85:15 dr). The enantioselectivity for major diastereomer (80% ee) was determined by HPLC (Daicel Chiralpak AD-H column,

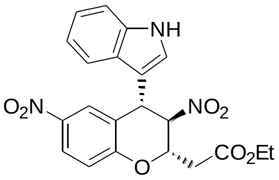

n-hexane/2-propanol 80:20, flow rate 1.0 mL·min^{-1}, detection at 254 nm), t_{major} = 31.1 min, t_{minor} = 28.2 min. The major diastereomer was purified by flash chromatography and obtained as a yellow oil. $[\alpha]_D^{25}$: −11.3 (*c* 1.04 g/100 mL, CH_2Cl_2). ^1H NMR (400 MHz, $CDCl_3$) δ 8.33 (s, 1H, NH), 8.07 (dd, J_1 = 8.8 Hz, J_2 = 2.4 Hz, 1H, ArH), 7.86 (d, *J* = 2.4 Hz, 1H, ArH), 7.39 (d, *J* = 8.0 Hz, 1H, ArH), 7.20–7.16 (m, 2H, ArH), 7.07 (d, *J* = 9.2 Hz, 1H, ArH), 6.98 (t, *J* = 7.6 Hz, 1H, ArH), 6.91 (d, *J* = 8.0 Hz, 1H, ArH), 5.32 (t, *J* = 10.0 Hz, 1H, CH), 5.12 (d, *J* = 10.4 Hz, 1H, CH), 5.04–4.99 (m, 1H, CH), 4.23 (q, *J* = 7.2 Hz, 2H, CH_2), 2.89–2.77 (m, 2H, CH_2), 1.29 (t, *J* = 7.2 Hz, 3H, CH_3) ppm. ^{13}C NMR (100 MHz, $CDCl_3$) δ 168.6, 157.5, 142.6, 137.0, 125.8, 124.9, 124.4, 124.2, 123.2, 122.8, 120.3, 118.5, 117.5, 112.0, 109.9, 86.7, 73.9, 61.4, 40.2, 36.7, 14.1 ppm. IR (KBr): *ν* 3417, 3062, 2983, 2928, 1733, 1586, 1556, 1521, 1481, 1459, 1342, 1258, 1208, 1095, 1042, 911, 842, 747 cm^{-1}. HRMS (ESI): calcd. for $C_{21}H_{20}N_3O_7$ [M + H]$^+$ 426.12958, found: 426.12945.

Ethyl 2-(4-(1*H*-indol-3-yl)-8-methoxy-3-nitro-chroman-2-yl)acetate (3i): Compound **3i** (the major diastereomer) was obtained according to the general procedure **A** (9.9 mg, 12% yield). It was analyzed to determine the diastereoselectivity of the reaction by the ratio of weight (minor diastereomer was not detected). Enantioselectivity for the major diastereomer (24% ee) was determined by HPLC (Daicel Chiralpak IA column, *n*-hexane/2-propanol 90:10, flow rate 1.0 mL·min^{-1}, detection at 254 nm), t_{major} = 21.6 min, t_{minor} = 18.9 min; The major diastereomer was purified by flash chromatography and obtained as a white solid; mp 160–162 °C. $[\alpha]_D^{25}$: +22.0 (*c* 0.88 g/100 mL, CH_2Cl_2). ^1H NMR (400 MHz, $CDCl_3$) δ 8.20 (s, 1H, NH), 7.35 (d, *J* = 8.4 Hz, 1H, ArH), 7.17–7.12 (m, 2H, ArH), 6.98–6.93 (m, 2H, ArH), 6.79–6.73 (m, 2H, ArH), 6.56–6.54 (m, 1H, ArH), 5.25 (t, *J* = 10.0 Hz, 1H, CH), 5.11 (d, *J* = 10.4 Hz, 1H, CH), 4.94–4.88 (m, 1H, CH), 4.23–4.17 (m, 2H, CH_2), 3.87 (s, 3H, CH_3), 2.91 (dd, J_1 = 7.6 Hz, J_2 = 16.0 Hz, 1H, CH_2), 2.79 (dd, J_1 = 4.0 Hz, J_2 = 16.0 Hz, 1H, CH_2), 1.28 (t, *J* = 7.0 Hz, 3H, CH_3) ppm. ^{13}C NMR (100 MHz, $CDCl_3$) δ 169.0, 148.1, 142.5, 136.8, 124.9, 124.4, 123.1, 122.5, 121.8, 121.0, 119.9, 119.3, 112.1, 111.6, 110.6, 88.2, 73.4, 61.2, 56.1, 40.4, 37.1, 14.1 ppm. IR (KBr): *ν* 3414, 2985, 1729, 1587, 1551, 1480, 1459, 1369, 1325, 1267, 1202, 1088, 1078, 767, 741 cm^{-1}. HRMS (ESI): calcd. for $C_{22}H_{23}N_2O_6$ [M + H]$^+$ 411.15506, found: 411.15549; calcd. for $C_{22}H_{26}N_3O_6$ [M + NH_4]$^+$ 428.18161, found: 428.18254.

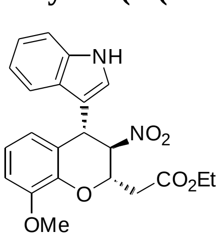

Ethyl 2-(8-ethoxy--4-(1*H*-indol-3-yl)-3-nitro-chroman-2-yl)acetate (3j): Compound **3j** (the mixture of the major and minor diastereomer) was obtained according to the general procedure **A** (40.3 mg, 47% yield). It was analyzed to determine the diastereoselectivity of the reaction by the ratio of weight (85:15 dr). Enantioselectivity for the major diastereomer (31% ee) was determined by HPLC (Daicel Chiralpak IA column, *n*-hexane/2-propanol 85:15, flow rate 0.5 mL·min^{-1}, detection at 254 nm), t_{major} = 22.2 min, t_{minor} = 19.0 min. The major diastereomer was purified by flash chromatography and obtained as a yellow oil. $[\alpha]_D^{25}$: +29.3 (*c* 0.75 g/100 mL, CH_2Cl_2). ^1H NMR (400 MHz, $CDCl_3$) δ 8.21 (s, 1H, NH), 7.34 (d, *J* = 8.4 Hz, 1H, ArH), 7.17–7.11 (m, 2H, ArH), 6.98–6.92 (m, 2H, ArH), 6.79–6.71 (m, 2H, ArH), 6.55 (d, *J* = 7.6 Hz, 1H, ArH), 5.20 (t, *J* = 10.0 Hz, 1H, CH), 5.11 (d, *J* = 10.4 Hz, 1H, CH), 4.94–4.88 (m, 1H, CH), 4.21 (q, *J* = 7.2 Hz, 2H, CH_2), 4.07 (q, *J* =

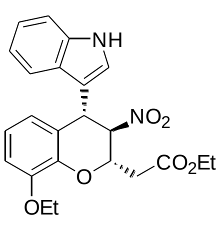

7.0 Hz, 2H, CH_2), 2.91 (dd, J_1 = 8.4 Hz, J_2 = 16.0 Hz, 1H, CH_2), 2.76 (dd, J_1 = 3.6 Hz, J_2 = 15.6 Hz, 1H, CH_2), 1.44 (t, J = 7.0 Hz, 3H, CH_3), 1.30 (t, J = 7.0 Hz, 3H, CH_3) ppm. ^{13}C NMR (100 MHz, $CDCl_3$) δ 169.1, 147.4, 136.8, 124.9, 124.3, 123.2, 122.5, 121.9, 121.2, 119.9, 119.3, 112.5, 112.1, 111.5, 88.5, 73.5, 64.9, 61.2, 40.4, 37.3, 14.9, 14.2 ppm. IR (KBr): ν 3411, 2982, 2935, 1737, 1552, 1472, 1366, 1342, 1266, 1203, 1094, 1065, 767, 744 cm^{-1}. HRMS (ESI): calcd. for $C_{23}H_{25}N_2O_6$ $[M + H]^+$ 425.17071, found: 425.17123; calcd. for $C_{23}H_{28}N_3O_6$ $[M + NH_4]^+$ 442.19726, found: 442.19779.

Ethyl 2-(6,8-dibromo-4-(1*H*-indol-3-yl)-3-nitro-chroman-2-yl)acetate (3k): Compound **3k** (the mixture of the major and minor diastereomer) was obtained according to the general procedure **A** (23.1 mg, 21% yield). It was analyzed to determine the diastereoselectivity and enantioselectivity of the reaction (72:28 dr, 57% ee for the major diastereomer) by HPLC (Daicel Chiralpak IA column, *n*-hexane/2-propanol 90:10, flow rate 1.0 mL·min^{-1}, detection at 254 nm), major diastereomer: t_{major} = 17.6 min, t_{minor} = 16.3 min; minor diastereomer: t_{major} = 10.0 min, t_{minor} = 9.3 min. The major diastereomer was purified by flash chromatography and obtained as a yellow oil. $[\alpha]_D^{25}$: +29.3 (*c* 0.75 g/100 mL, CH_2Cl_2). ^{1}H NMR (400 MHz, $CDCl_3$) δ 8.24 (s, 1H, NH), 7.62 (d, J = 2.0 Hz, 1H, ArH), 7.56 (d, J = 8.0 Hz, 1H, ArH), 7.41 (d, J = 8.0 Hz, 1H, ArH), 7.30–7.18 (m, 4H, ArH), 6.61 (d, J = 2.0 Hz, 1H, ArH), 5.16 (t, J = 1.8 Hz, 1H, CH), 5.02 (s, 1H, CH), 4.80–4.77 (m, 1H, CH), 4.17–4.08 (m, 2H, CH_2), 2.97 (dd, J_1 = 8.4 Hz, J_2 = 16.8 Hz, 1H, CH_2), 2.75 (dd, J_1 = 5.2 Hz, J_2 = 16.8 Hz, 1H, CH_2), 1.18 (t, J = 7.2 Hz, 3H, CH_3) ppm. ^{13}C NMR (100 MHz, $CDCl_3$) δ 169.3, 149.4, 136.4, 134.2, 132.0, 125.1, 124.8, 123.9, 123.3, 120.6, 117.5, 116.1, 113.9, 111.9, 111.8, 84.0, 69.1, 61.3, 36.6, 36.3, 14.0 ppm. IR (KBr): ν 3415, 3063, 2982, 2930, 1733, 1552, 1455, 1366, 1316, 1286, 1244, 1191, 1097, 1026, 921, 864, 745 cm^{-1}. HRMS (ESI): calcd. for $C_{21}H_{19}Br_2N_2O_5$ $[M + H]^+$ 536.96552, found: 536.96564; calcd. for $C_{21}H_{22}$ $Br_2N_3O_5$ $[M + NH_4]^+$ 553.99207, found: 553.99228.

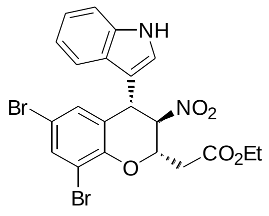

General procedure (B) for the catalytic asymmetric tandem Friedel–Crafts alkylation/Michael addition reaction of indoles with nitroolefin enoates: To a dried Schlenk tube were added $Zn(OTf)_2$ (7.3 mg, 0.02 mmol), ligand **I** (12.2 mg, 0.02 mmol) under argon followed by addition of the toluene (3 mL). The solution was stirred at room temperature for 0.5 h and then nitroolefin enoate **1** (0.2 mmol) was added. The mixture was stirred for 10 min then the indole **2** (0.3 mmol) was added. After stirring for 72 h at −10 °C, Et_3N (100 mg, 1 mmol) was added and stirred for another 24 h at room remperature. The solvent was removed under vacuum. Purification by column chromatography afforded the desired product **3**.

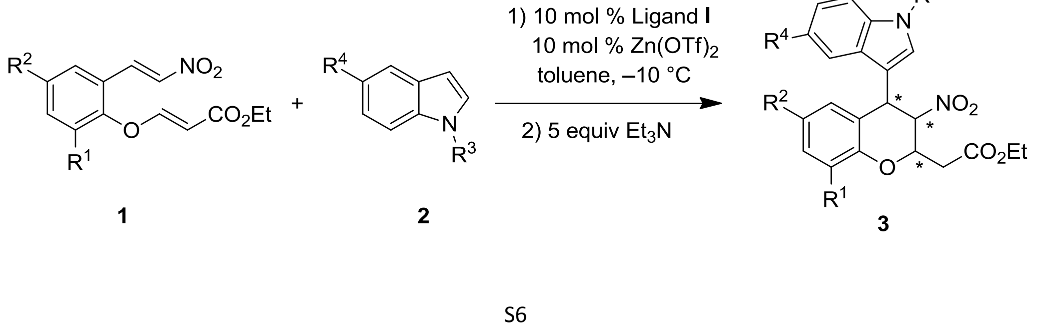

1 **2** **3**

Ethyl 2-(4-(1*H*-indol-3-yl)-3-nitro-chroman-2-yl)acetate (3a): Compound **3a** (the mixture of the major and minor diastereomer) was obtained according to the general procedure **B** (67.2 mg, 88% yield). It was analyzed to determine the diastereoselectivity and enantioselectivity of the reaction (92:8 dr, 92% ee for the major diastereomer and 88% ee for the minor diastereomer) by HPLC (Daicel Chiralpak IA column, *n*-hexane/2-propanol 85:15, flow rate 0.5 mL·min^{-1}, detection at 254 nm), major diastereomer: t_{major} = 19.8 min, t_{minor} = 18.9 min; minor diastereomer: t_{major} = 16.9 min, t_{minor} = 14.4 min. $[\alpha]_D^{25}$ = +90.1 (*c* 3.02 g/100 mL, CH_2Cl_2).

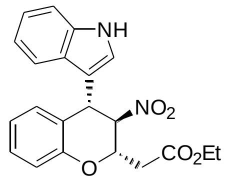

Ethyl 2-(4-(5-methyl-1*H*-indol-3-yl)-3-nitro-chroman-2-yl)acetate (3b): Compound **3b** (the mixture of the major and minor diastereomer) was obtained according to the general procedure **B** (69.1 mg, 88% yield). It was analyzed to determine the diastereoselectivity of the reaction by the ratio of weight (44:56 dr). Enantioselectivity for major diastereomer (98% ee) and minor diastereomer (62% ee) were determined by HPLC separately (Daicel Chiralpak IA column, *n*-hexane/2-propanol 90:10, flow rate 0.5 mL·min^{-1}, detection at 254 nm and IB column, *n*-hexane/2-propanol 90:10, flow rate 0.5 mL·min^{-1}, detection at 254 nm), major diastereomer: t_{major} = 23.3 min, t_{minor} = 19.0 min; minor diastereomer: t_{major} = 28.0 min, t_{minor} = 30.7 min. The major diastereomer was purified by flash chromatography and obtained as a yellow oil. $[\alpha]_D^{25}$(major diastereomer): −2.8 (*c* 2.17 g/100 mL, CH_2Cl_2), $[\alpha]_D^{25}$(minor diastereomer): +29.9 (*c* 1.78 g/100 mL, CH_2Cl_2).

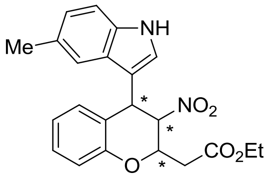

Ethyl 2-(4-(5-methoxy-1*H*-indol-3-yl)-3-nitro-chroman-2-yl)acetate (3c): Compound **3c** (the mixture of the major and minor diastereomer) was obtained according to the general procedure **B** (78.8 mg, 96% yield). It was analyzed to determine the diastereoselectivity of the reaction by the ratio of weight (28:72 dr). Enantioselectivity for the major diastereomer (99% ee) and the minor diastereomer (73% ee) were determined by HPLC separately (Daicel Chiralpak IB column, *n*-hexane/2-propanol 90:10, flow rate 0.5 mL·min^{-1}, detection at 254 nm and IA column, *n*-hexane/2-propanol 90:10, flow rate 0.5 mL·min^{-1}, detection at 254 nm), major diastereomer: t_{major} = 34.8 min, t_{minor} = 33.2 min; minor diastereomer: t_{major} = 42.9 min, t_{minor} = 40.8 min.
The major diastereomer was purified by flash chromatography and obtained as a yellow oil. $[\alpha]_D^{25}$(major diastereomer): −3.15 (*c* 2.67 g/100 mL, CH_2Cl_2), $[\alpha]_D^{25}$(minor diastereomer): +34.8 (*c* 1.36 g/100 mL, CH_2Cl_2).

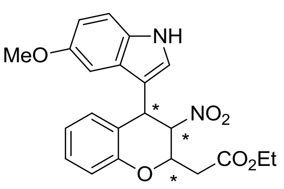

Ethyl 2-(4-(5-chloro-1*H*-indol-3-yl)-3-nitro-chroman-2-yl)acetate (3d): Compound **3d** (the mixture of the major and minor diastereomer) was obtained according to the general procedure **B** (47.8 mg, 58% yield). It was analyzed to determine the diastereoselectivity and enantioselectivity of the reaction (76:24 dr, 82% ee for the major diastereomer and 53% ee for the minor diastereomer) by HPLC (Daicel Chiralpak IB column, *n*-hexane/2-propanol 90:10, flow rate 0.5 mL·min^{-1}, detection at 254 nm), major diastereomer:

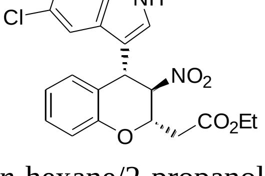

t_{major} = 36.4 min, t_{minor} = 41.2 min; minor diastereomer: t_{major} = 28.6 min, t_{minor} = 27.1 min. The major diastereomer was purified by flash chromatography and obtained as a yellow oil. $[\alpha]_D^{25}$(major diastereomer): +17.6 (*c* 1.67 g/100 mL, CH_2Cl_2).

Ethyl 2-(4-(1-methyl-1*H*-indol-3-yl)-3-nitro-chroman-2-yl)acetate (3e): Compound **3e** (the mixture of the major and minor diastereomer) was obtained according to the general procedure **B** (70.1 mg, 89% yield). It was analyzed to determine the diastereoselectivity of the reaction by the ratio of weight (82:18 dr). Enantioselectivity for major diastereomer (65% ee) and minor diastereomer (79% ee) were determined by HPLC separately (Daicel Chiralpak IA column, *n*-hexane/2-propanol = 95:5, flow rate 0.5 mL·min^{-1}, detection at 254 nm), major diastereomer: t_{major} = 29.0 min, t_{minor} = 22.1 min; minor diastereomer: t_{major} = 23.7 min, t_{minor} = 21.4 min. The major diastereomer was purified by flash chromatography and obtained as a yellow oil. $[\alpha]_D^{25}$(major diastereomer): +53.0 (*c* 2.81 g/100 mL, CH_2Cl_2), $[\alpha]_D^{25}$(minor diastereomer): −15.1 (*c* 1.07 g/100 mL, CH_2Cl_2).

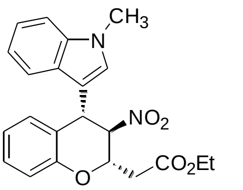

Ethyl 2-(6-chloro-4-(1*H*-indol-3-yl)-3-nitro-chroman-2-yl)acetate (3f): Compound **3f** (the mixture of the major and minor diastereomer) was obtained according to the general procedure **B** (71.4 mg, 86% yield). It was analyzed to determine the diastereoselectivity and enantioselectivity of the reaction (74:26 dr, 95% ee for the major diastereomer, 95% ee for the minor diastereomer) by HPLC (Daicel Chiralpak IA column, *n*-hexane/2-propanol 90:10, flow rate 0.5 mL·min^{-1}, detection at 254 nm), major diastereomer: t_{major} = 32.2 min, t_{minor} = 28.5 min; minor diastereomer: t_{major} = 22.1 min, t_{minor} = 20.1 min. The major diastereomer was purified by flash chromatography and obtained as a yellow oil. $[\alpha]_D^{25}$(major diastereomer): +10.0 (*c* 2.07 g/100 mL, CH_2Cl_2).

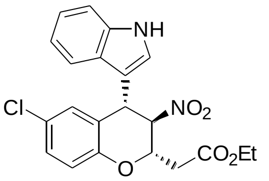

Ethyl 2-(6-bromo-4-(1*H*-indol-3-yl)-3-nitro-chroman-2-yl)acetate (3g): Compound **3g** (the mixture of the major and minor diastereomer) was obtained according to the general procedure **B** (91.8 mg, 100% yield). It was analyzed to determine the diastereoselectivity and enantioselectivity of the reaction (60:40 dr, 91% ee for the major diastereomer, 93% ee for the minor diastereomer) by HPLC (Daicel Chiralpak IB column, *n*-hexane/2-propanol 90:10, flow rate 0.5 mL·min^{-1}, detection at 254 nm), major diastereomer: t_{major} = 37.9 min, t_{minor} = 41.6 min; minor diastereomer: t_{major} = 34.0 min, t_{minor} = 26.6 min. $[\alpha]_D^{25}$(major diastereomer): +7.4 (*c* 2.74 g/100 mL, CH_2Cl_2).

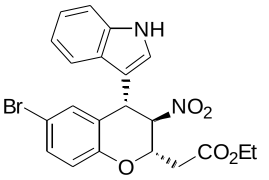

Ethyl 2-(4-(1*H*-indol-3-yl)-3,6-dinitro-chroman-2-yl)acetate (3h): Compound **3h** (the mixture of the major and minor diastereomer) was obtained according to the general procedure **B** (76.1 mg, 89% yield). It was analyzed to determine the diastereoselectivity and enantioselectivity of the reaction (49:51 dr, 95% ee for both diastereomers) by HPLC (Daicel Chiralpak IA + AD-H column, *n*-hexane/2-propanol 80:20, flow rate 1.0 mL·min^{-1}, detection at 254 nm), major diastereomer: t_{major} = 18.4 min, t_{minor} = 22.3 min;

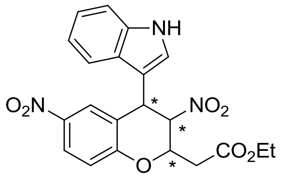

minor diastereomer: t_{major} = 28.5 min, t_{minor} = 26.5 min. Both diastereomers were purified by flash chromatography and obtained as yellow oils. $[\alpha]_D^{25}$(major diastereomer): −160.0 (*c* 1.32 g/100 mL, CH_2Cl_2).

Ethyl 2-(4-(1*H*-indol-3-yl)-8-methoxy-3-nitro-chroman-2-yl)acetate (3i): Compound **3i** (the mixture of the major and minor diastereomer) was obtained according to the general procedure **B** (69.6 mg, 85% yield). It was analyzed to determine the diastereoselectivity of the reaction by the ratio of weight (58:42 dr). Enantioselectivity for major diastereomer (39% ee) and minor diastereomer (83% ee) were determined by HPLC separately (Daicel Chiralpak IA column, *n*-hexane/2-propanol 90:10, flow rate 1.0 mL·min^{-1}, detection at 254 nm and IA column, *n*-hexane/2-propanol 95:5, flow rate 0.5 mL·min^{-1}, detection at 254 nm), major diastereomer: t_{major} = 23.0 min, t_{minor} = 20.2 min; minor diastereomer: t_{major} = 79.4 min, t_{minor} = 85.7 min. The major diastereomer was purified by flash chromatography and obtained as a white solid; mp 167−172 °C. The minor diastereomer was purified by flash chromatography and obtained as yellow oil. $[\alpha]_D^{25}$ (major diastereomer): +39.2 (*c* 1.55 g/100 mL, CH_2Cl_2), $[\alpha]_D^{25}$ (minor diastereomer): +28.0 (*c* 1.27 g/100 mL, CH_2Cl_2).

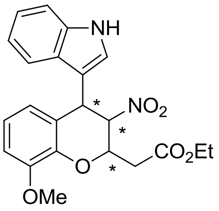

Ethyl 2-(8-ethoxy-4-(1*H*-indol-3-yl)-3-nitro-chroman-2-yl)acetate (3j): Compound **3j** (the mixture of the major and minor diastereomer) was obtained according to the general procedure **B** (79.7 mg, 94% yield). It was analyzed to determine the diastereoselectivity of the reaction by the ratio of weight (54:46 dr). Enantioselectivity for major diastereomer (30% ee) and minor diastereomer (97% ee) were determined by HPLC separately (Daicel Chiralpak IA column, *n*-hexane/2-propanol 85:15, flow rate 0.5 mL·min^{-1}, detection at 254 nm and AD-H column, *n*-hexane/2-propanol 90:10, flow rate 0.5 mL·min^{-1}, detection at 254 nm), major diastereomer: t_{major} = 22.5 min, t_{minor} = 19.5 min; minor diastereomer: t_{major} = 51.5 min, t_{minor} = 49.2 min. Both diastereomers were purified by flash chromatography and obtained as a yellow oil. $[\alpha]_D^{25}$ (major diastereomer): +38.4 (*c* 1.78 g/100 mL, CH_2Cl_2), $[\alpha]_D^{25}$ (minor diastereomer): +58.0 (*c* 1.57 g/100 mL, CH_2Cl_2).

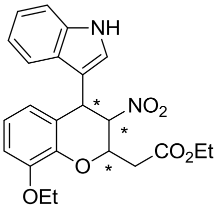

Ethyl 2-(6,8-dibromo-4-(1*H*-indol-3-yl)-3-nitro-chroman-2-yl)acetate (3k): Compound **3k** (the mixture of the major and minor diastereomer) was obtained according to general procedure **B** (81.2 mg, 75% yield). It was analyzed to determine the diastereoselectivity and enantioselectivity of the reaction (38:62 dr, 90% ee for the major diastereomer, 53% ee for the minor diastereomer) by HPLC (Daicel Chiralpak IA column, *n*-hexane/2-propanol 90:10, flow rate 1.0 mL·min^{-1}, detection at 254 nm), major diastereomer: t_{major} = 9.8 min, t_{minor} = 9.2 min; minor diastereomer: t_{major} = 17.5 min, t_{minor} = 16.3 min. The major diastereomer was purified by flash chromatography and obtained as a white solid; mp 189−191 °C. The minor diastereomer was purified by flash chromatography and obtained as a white solid, mp 91−95 °C. $[\alpha]_D^{25}$(major diastereomer): +41.0 (*c* 1.46 g/100 mL, CH_2Cl_2), $[\alpha]_D^{25}$(minor diastereomer): +39.2 (*c* 0.97 g/100 mL, CH_2Cl_2).

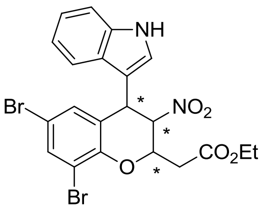

References:

1. (a) Liu, H.; Xu, J.-X.; Du, D. M. *Org. Lett.* **2007**, *9*, 4725. (b) Lu, S. F.; Du, D. M.; Zghang, S.-W.; Xu, J. *Tetrahedron: Asymmetry* **2004**, *15* (21), 3433.
2. (a) Ciganek, E. *Synthesis* **1995**, 1311. (b) Lu, L.-Q.; Li, F.; An, J.; Zhang, J.-J.; An, X.-L.; Hua, Q.-L.; Xiao, W.-J. *Angew. Chem., Int. Ed.* **2009**, *48*, 9542.
3. Li, C.; Liu, F.-L.; Zou, Y.-Q.; Lu, L.-Q.; Chen, J.-R.; Xiao, W.-J. *Synthesis* **2013,** *45*, 601–608 (See for a similar preparation of a stereoisomer of **3e**.)

Copies of ^{1}H and ^{13}C NMR spectra of new compounds

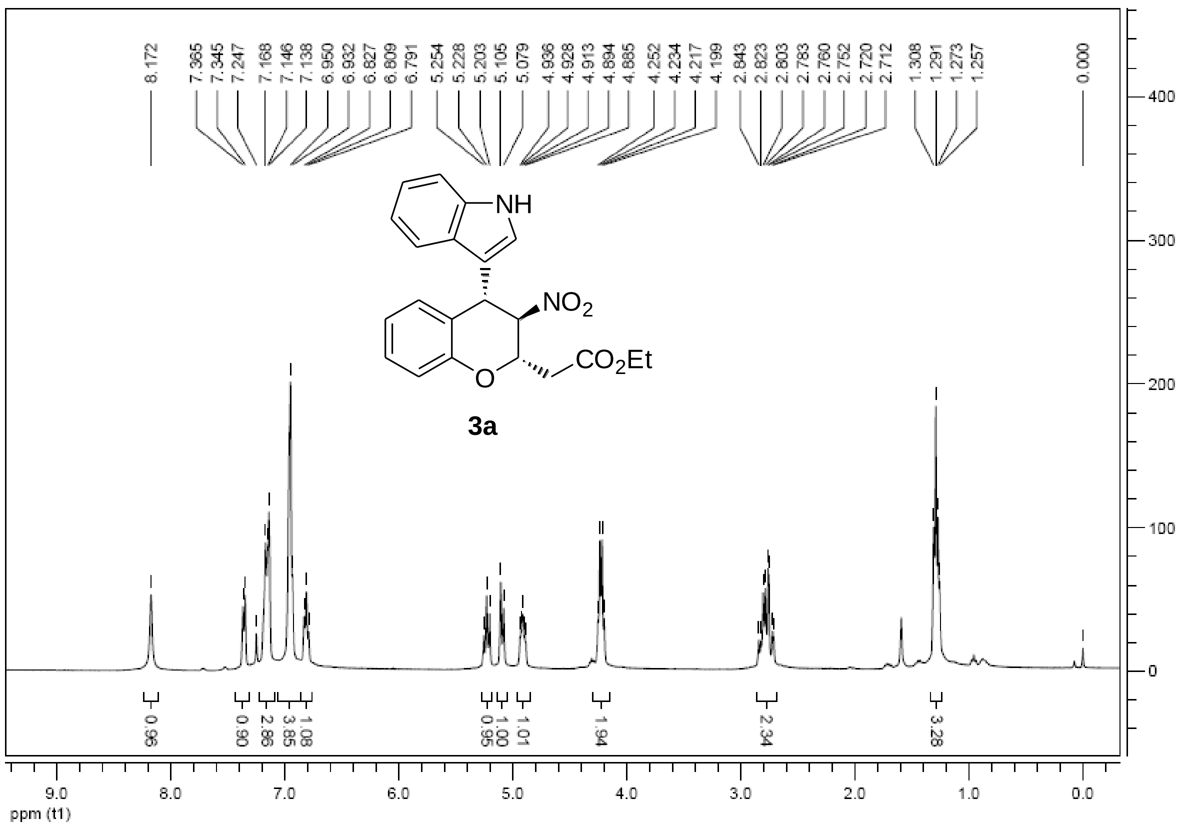

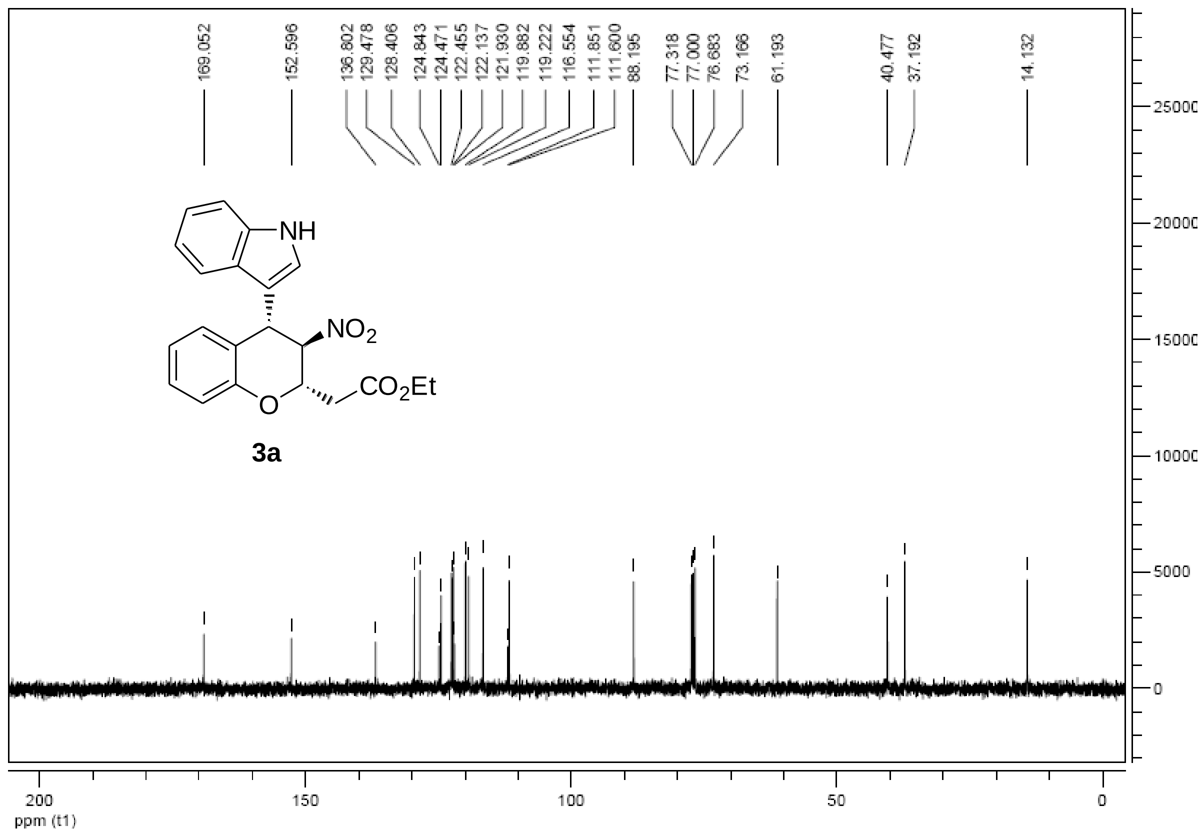

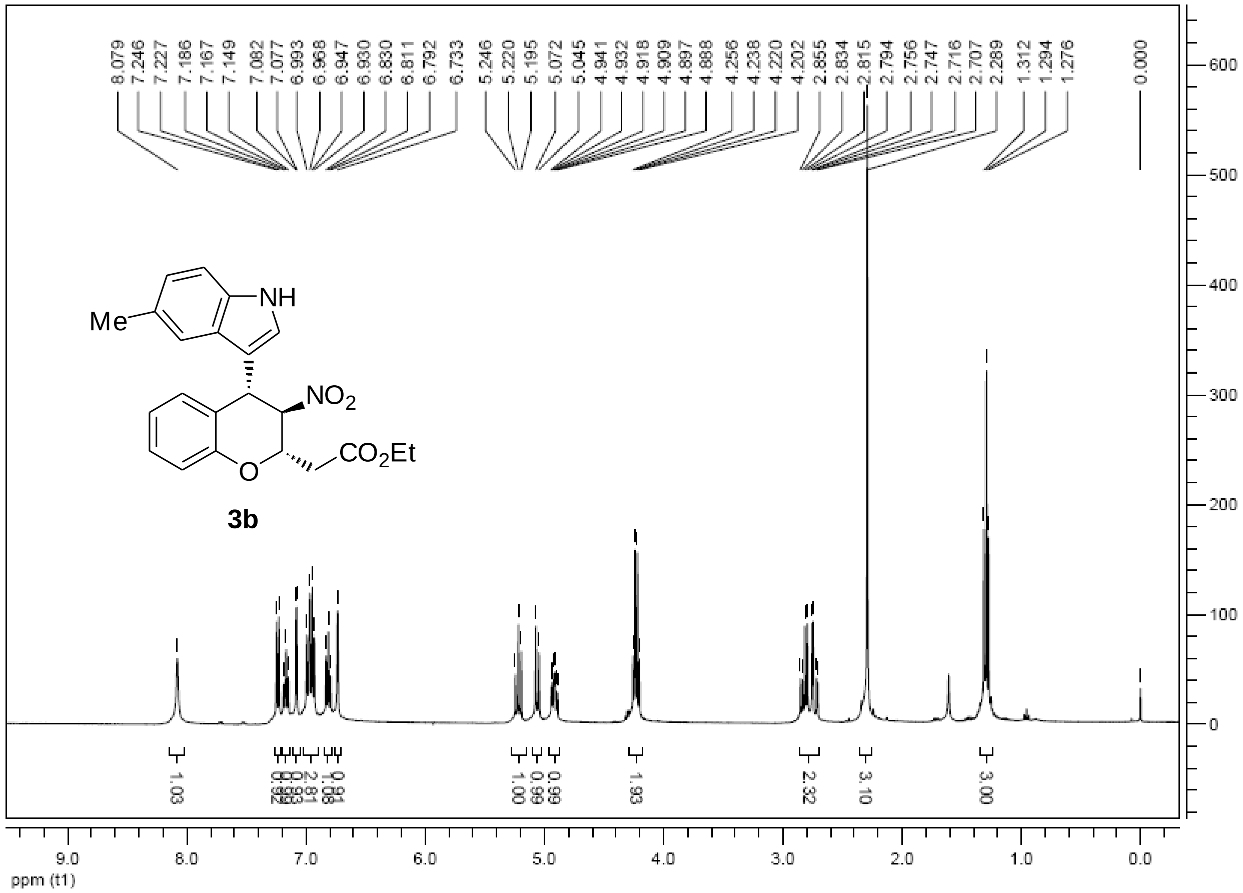
8.079 7.246 7.227 7.186 7.167 7.149 7.082 7.077 6.993 6.968 6.947 6.930 6.830 6.811 6.792 6.733 5.246 5.220 5.195 5.072 5.045 4.941 4.932 4.918 4.909 4.897 4.888 4.256 4.238 4.220 4.202 2.855 2.834 2.815 2.794 2.756 2.747 2.716 2.707 2.289 1.312 1.294 1.276 0.000
Me
NH
NO_2
CO_2Et
O
3b
1.03
2.81
1.00
0.99
0.99
1.93
2.32
3.10
3.00
ppm (t1)

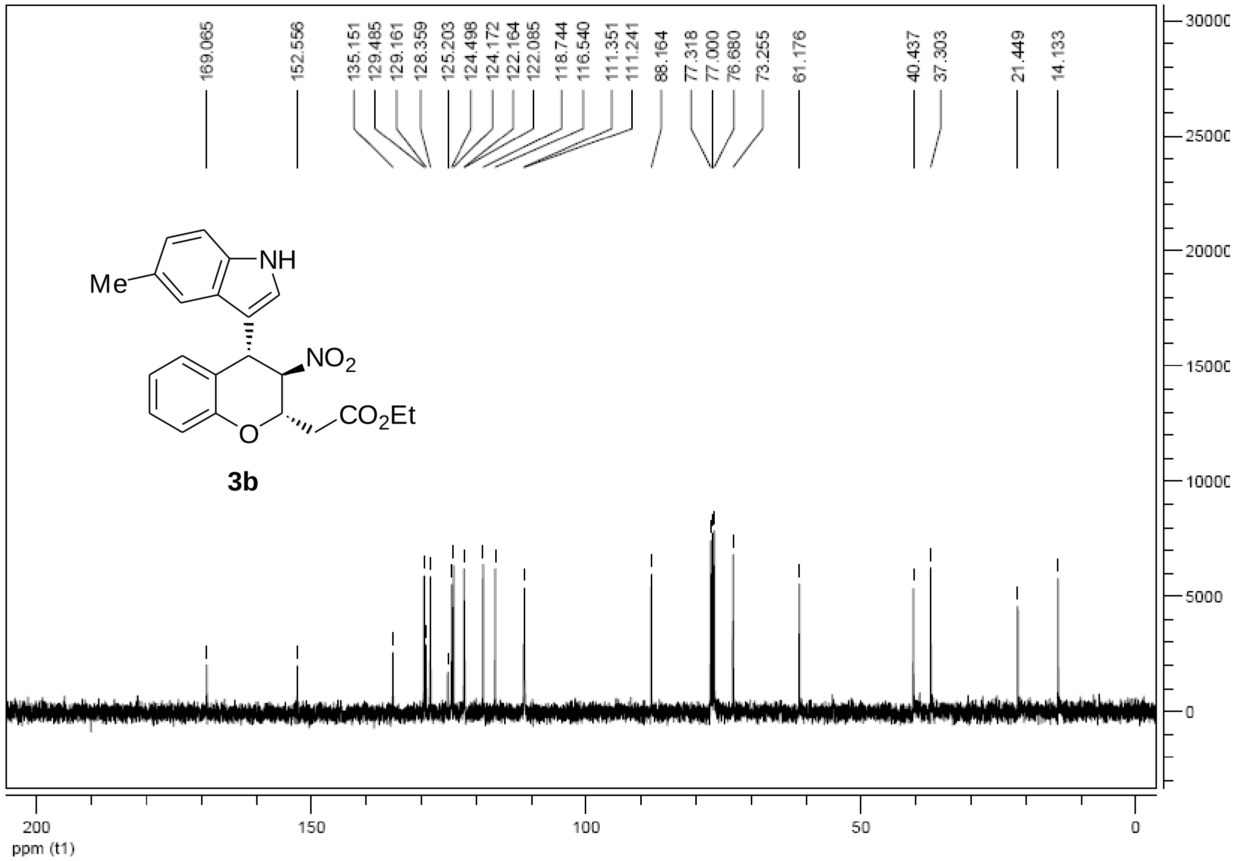
169.065 152.556 135.151 129.485 129.161 128.359 125.203 124.498 124.172 122.164 122.085 118.744 116.540 111.351 111.241 88.164 77.318 77.000 76.680 73.255 61.176 40.437 37.303 21.449 14.133
Me
NH
NO_2
CO_2Et
O
3b
ppm (t1)

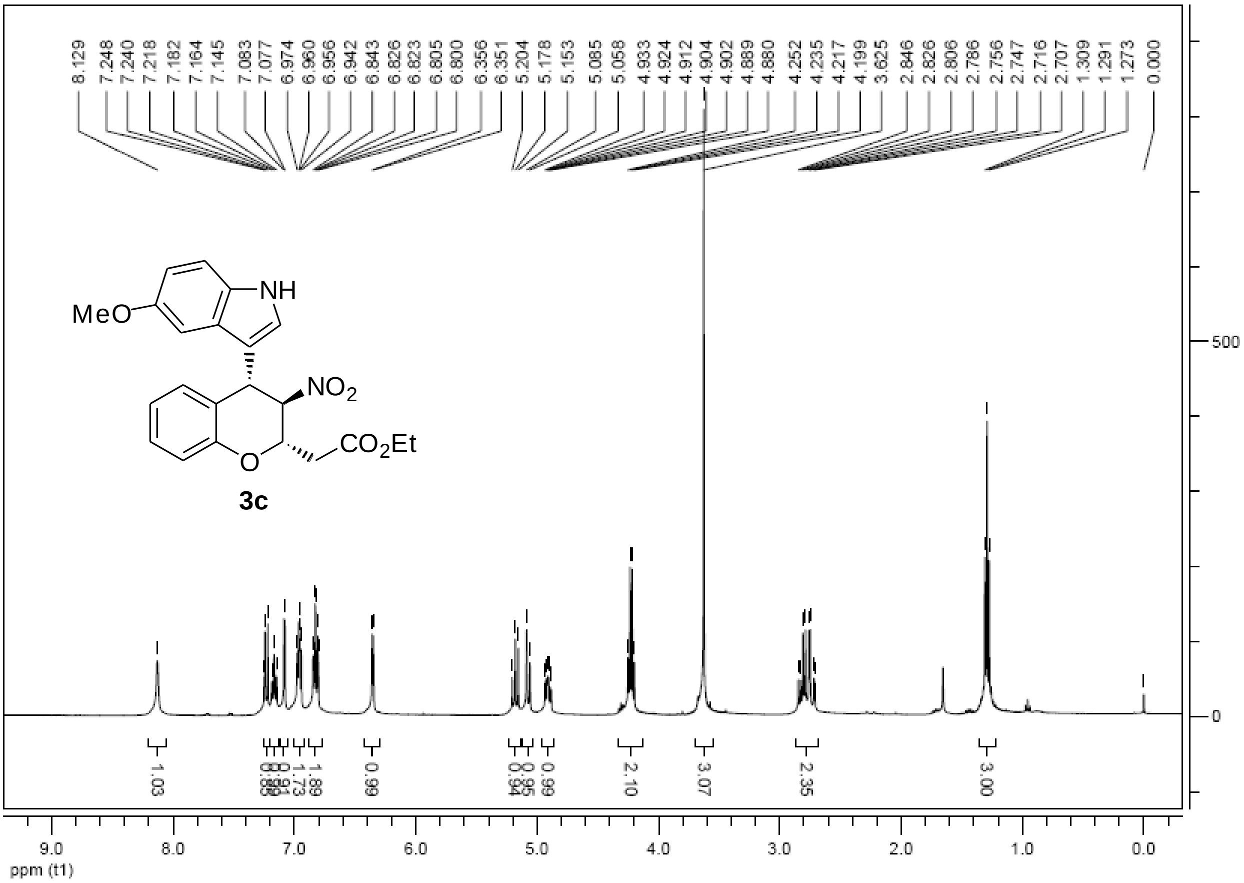
8.129 7.248 7.240 7.218 7.182 7.164 7.145 7.083 7.077 6.974 6.960 6.956 6.942 6.843 6.826 6.823 6.805 6.800 6.356 6.351 5.204 5.178 5.153 5.085 5.058 4.933 4.924 4.912 4.904 4.902 4.889 4.880 4.252 4.235 4.217 4.199 3.625 2.846 2.826 2.806 2.786 2.756 2.747 2.716 2.707 1.309 1.291 1.273 0.000
MeO
NH
NO2
CO2Et
O
3c
1.03
1.73
1.89
0.99
0.99
2.10
3.07
2.35
3.00
500
0
9.0 8.0 7.0 6.0 5.0 4.0 3.0 2.0 1.0 0.0
ppm (t1)

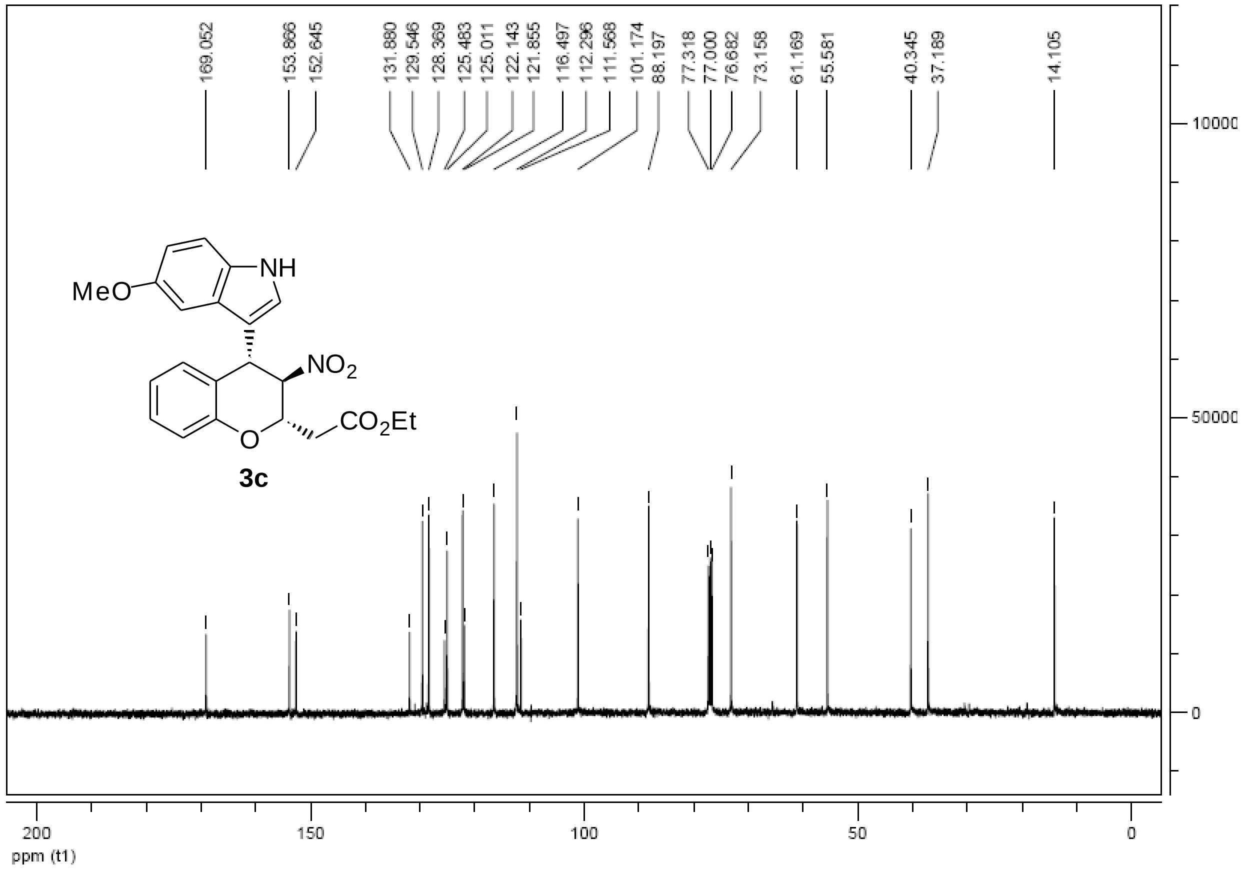
169.052 153.866 152.645 131.880 129.546 128.369 125.483 125.011 122.143 121.855 116.497 112.296 111.568 101.174 88.197 77.318 77.000 76.682 73.158 61.169 55.581 40.345 37.189 14.105
MeO
NH
NO2
CO2Et
O
3c
100000
50000
0
200 150 100 50 0
ppm (t1)

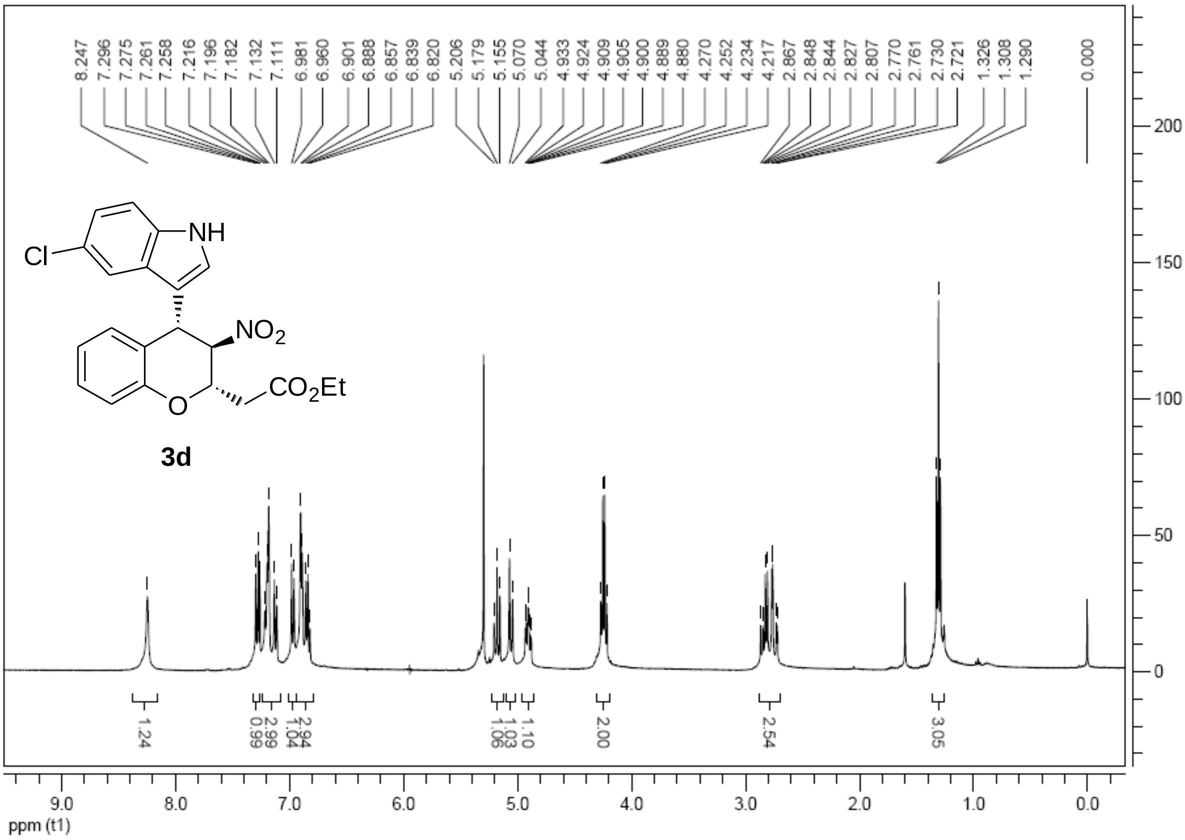
8.247 7.296 7.275 7.261 7.258 7.216 7.196 7.182 7.132 7.111 6.981 6.960 6.901 6.888 6.857 6.839 6.820 5.206 5.179 5.155 5.070 5.044 4.933 4.924 4.909 4.905 4.900 4.889 4.880 4.270 4.252 4.234 4.217 2.867 2.848 2.844 2.827 2.807 2.770 2.761 2.730 2.721 1.326 1.308 1.290 0.000
Cl
NH
NO2
CO2Et
O
3d
1.24 0.99 2.99 1.04 2.94 1.06 1.03 1.10 2.00 2.54 3.05
ppm (t1)

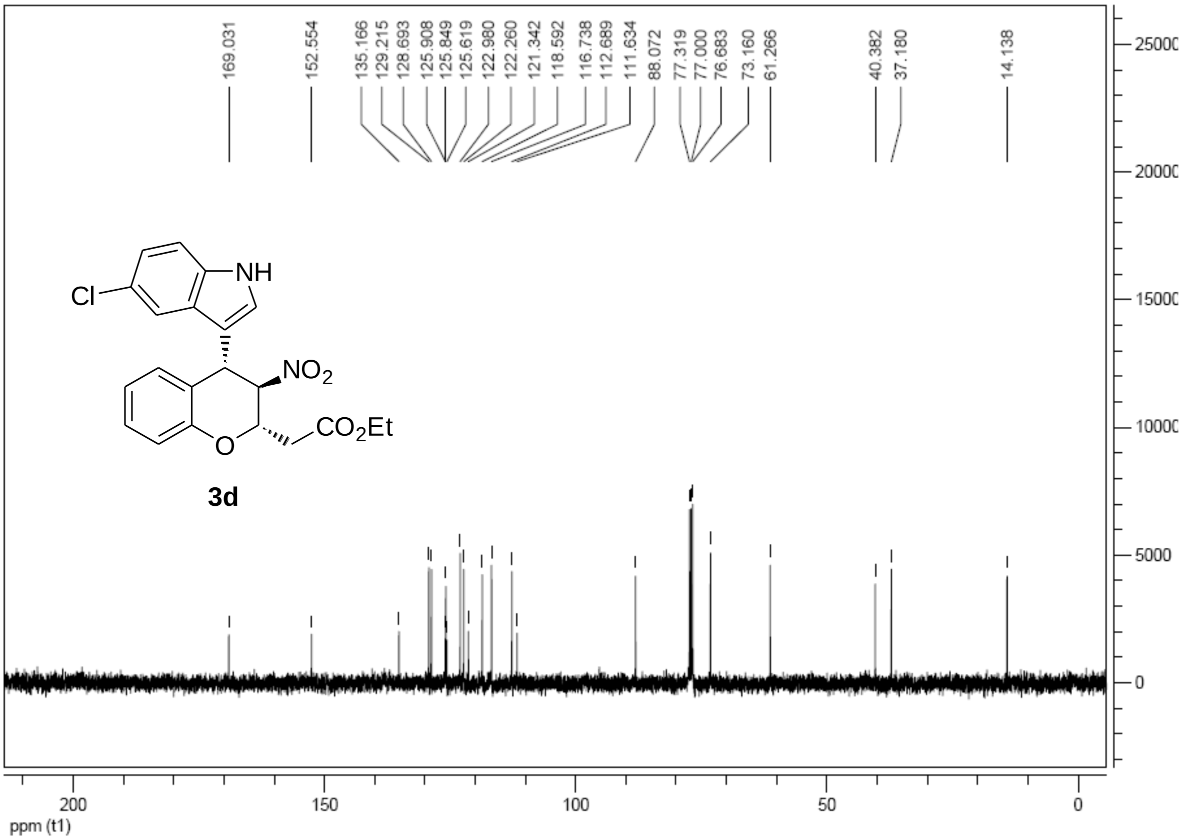
169.031 152.554 135.166 129.215 128.693 125.908 125.849 125.619 122.980 122.260 121.342 118.592 116.738 112.689 111.634 88.072 77.319 77.000 76.683 73.160 61.266 40.382 37.180 14.138
Cl
NH
NO2
CO2Et
O
3d
ppm (t1)

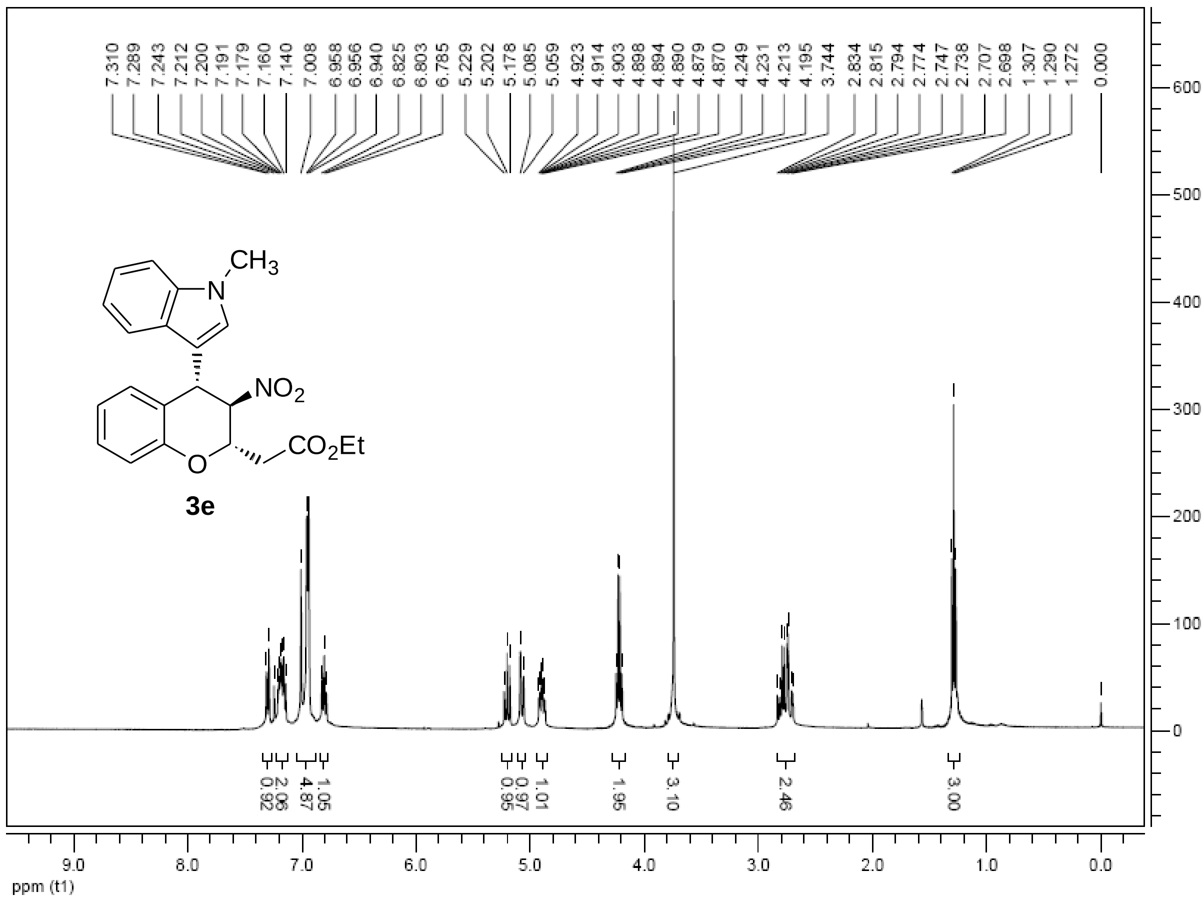

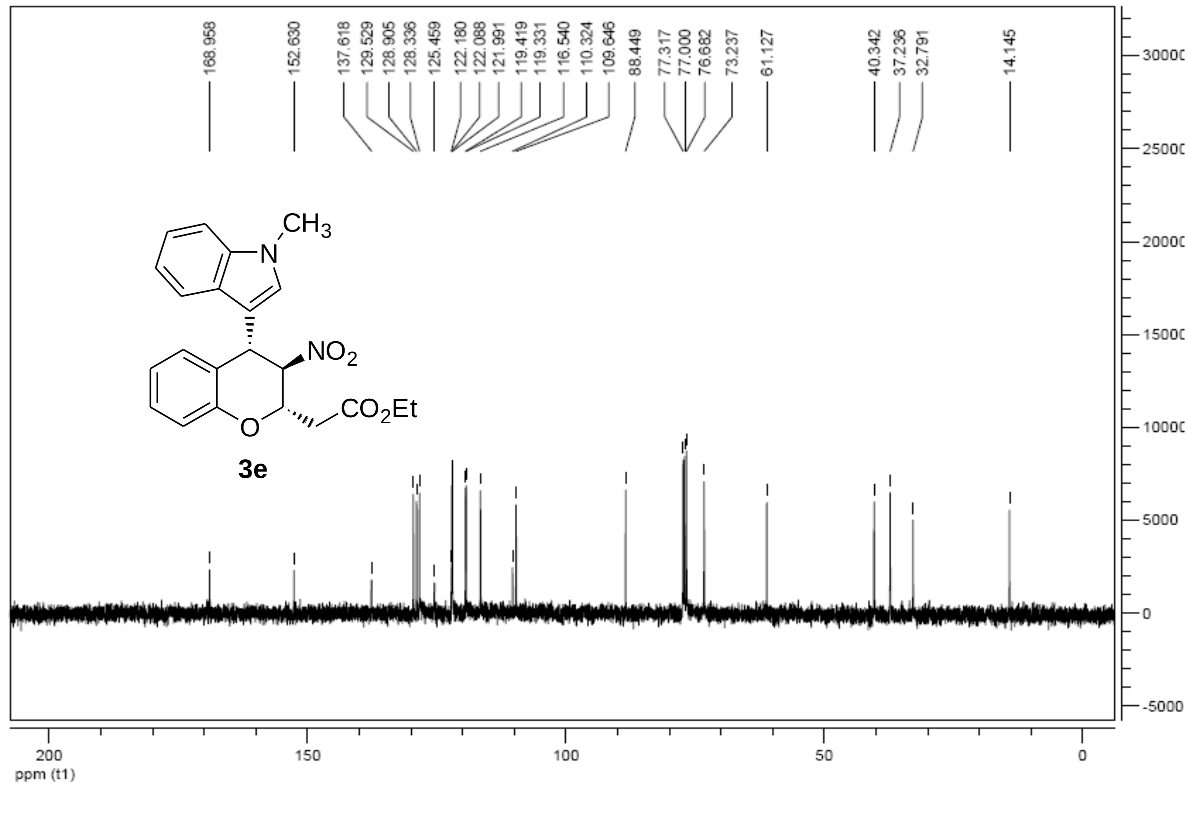

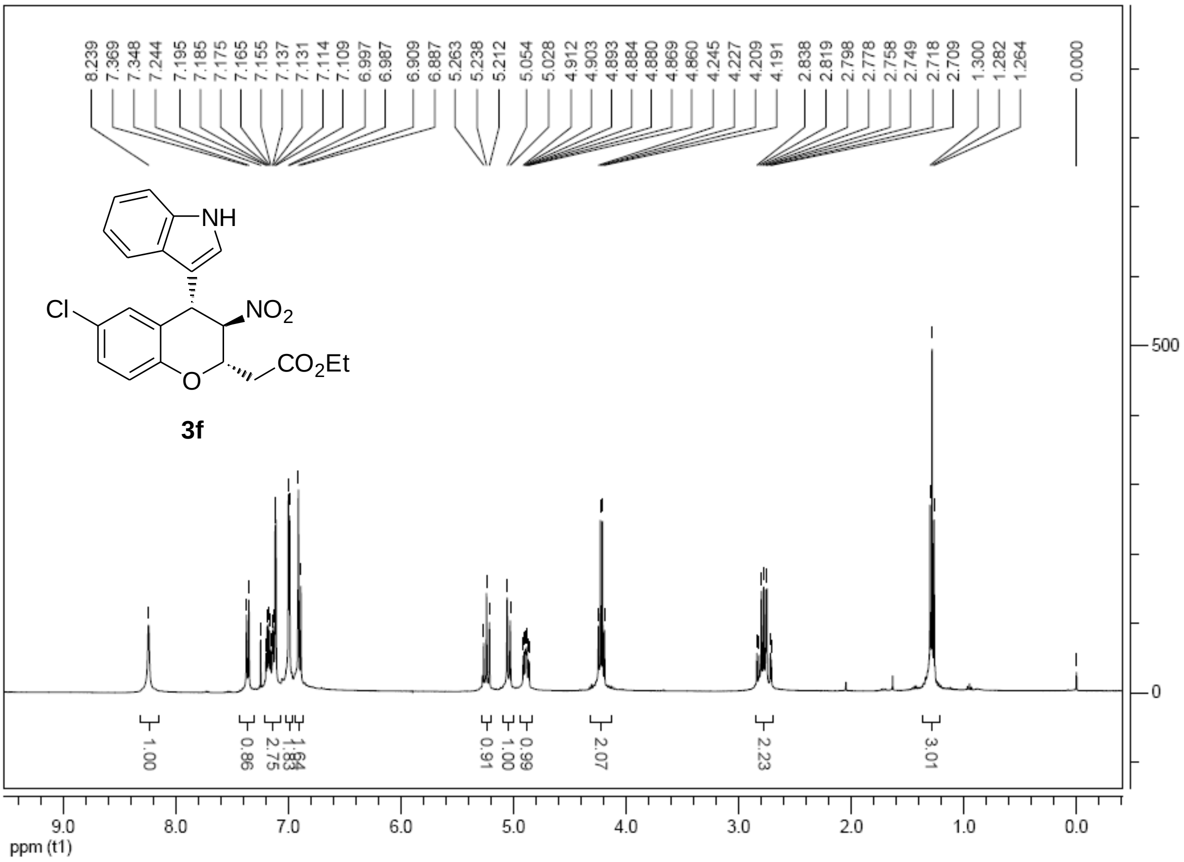

8.239
7.369
7.348
7.244
7.195
7.185
7.175
7.165
7.155
7.137
7.131
7.114
7.109
6.997
6.987
6.909
6.887
5.263
5.238
5.212
5.054
5.028
4.912
4.903
4.893
4.884
4.880
4.869
4.860
4.245
4.227
4.209
4.191
2.838
2.819
2.798
2.778
2.758
2.749
2.718
2.709
1.300
1.282
1.264
0.000
NH
Cl
NO2
CO2Et
O
3f
500
0
1.00
0.86
2.75
1.83
1.64
0.91
1.00
0.99
2.07
2.23
3.01
9.0
8.0
7.0
6.0
5.0
4.0
3.0
2.0
1.0
0.0
ppm (t1)

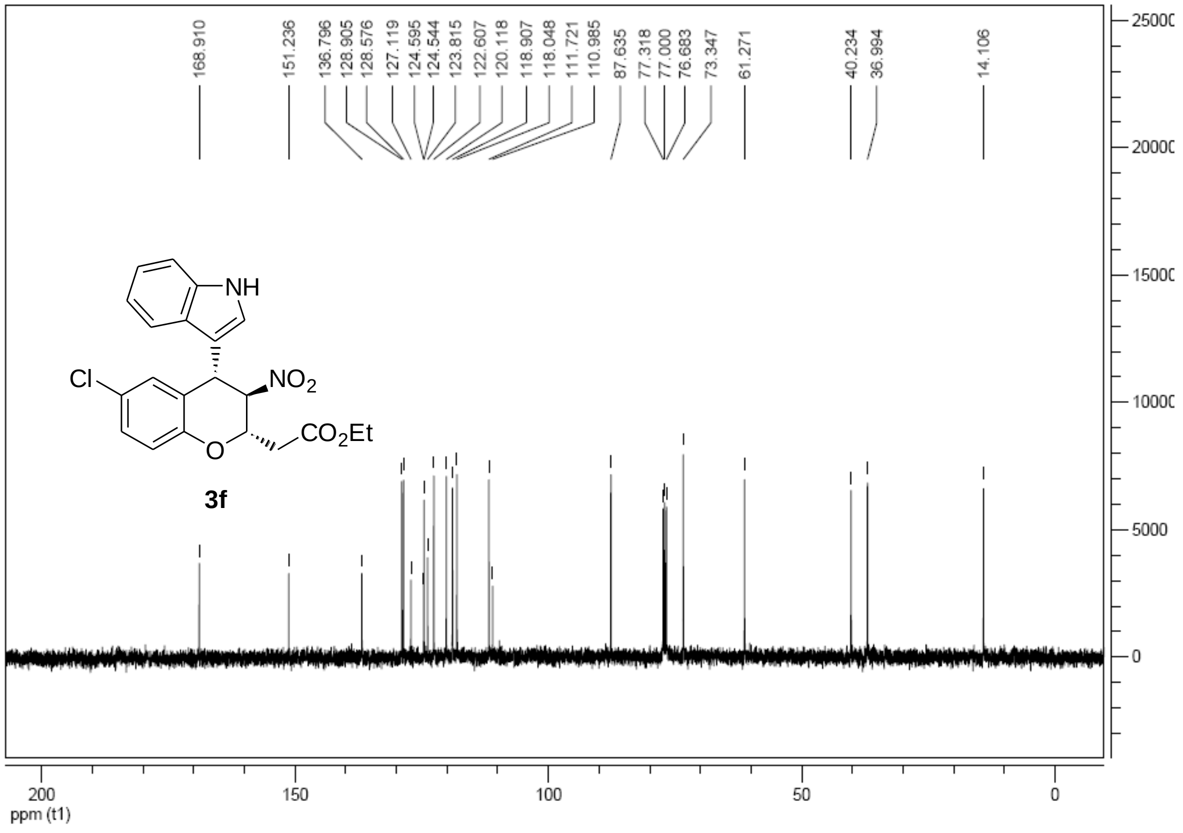

168.910
151.236
136.796
128.905
128.576
127.119
124.595
124.544
123.815
122.607
120.118
118.907
118.048
111.721
110.985
87.635
77.318
77.000
76.683
73.347
61.271
40.234
36.994
14.106
NH
Cl
NO2
CO2Et
O
3f
25000
20000
15000
10000
5000
0
200
150
100
50
0
ppm (t1)

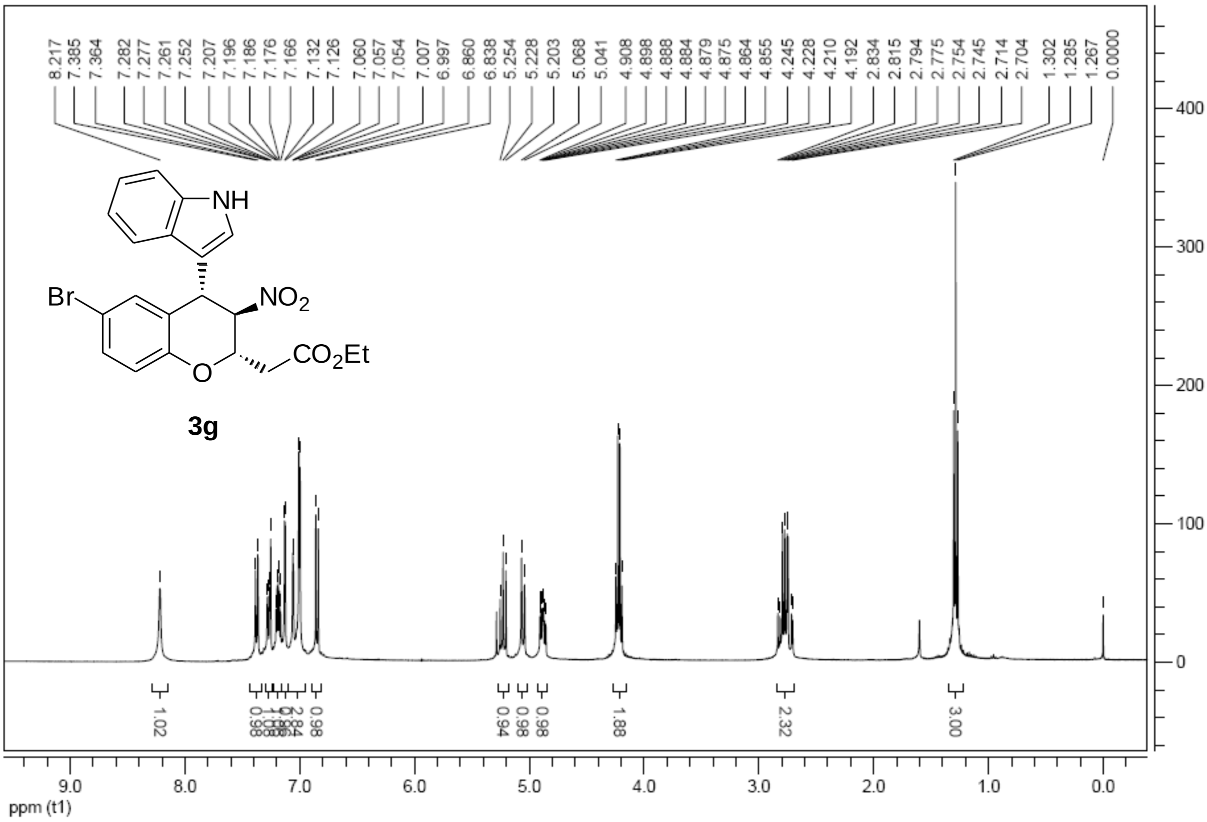

8.217
7.385
7.364
7.282
7.277
7.261
7.252
7.207
7.196
7.186
7.176
7.166
7.132
7.126
7.060
7.057
7.054
7.007
6.997
6.860
6.838
5.254
5.228
5.203
5.068
5.041
4.908
4.898
4.888
4.884
4.879
4.875
4.864
4.855
4.245
4.228
4.210
4.192
2.834
2.815
2.794
2.775
2.754
2.745
2.714
2.704
1.302
1.285
1.267
0.0000
NH
Br
NO2
CO2Et
O
3g
1.02
0.98
0.98
2.84
0.98
0.94
0.98
0.98
1.88
2.32
3.00
ppm (t1)

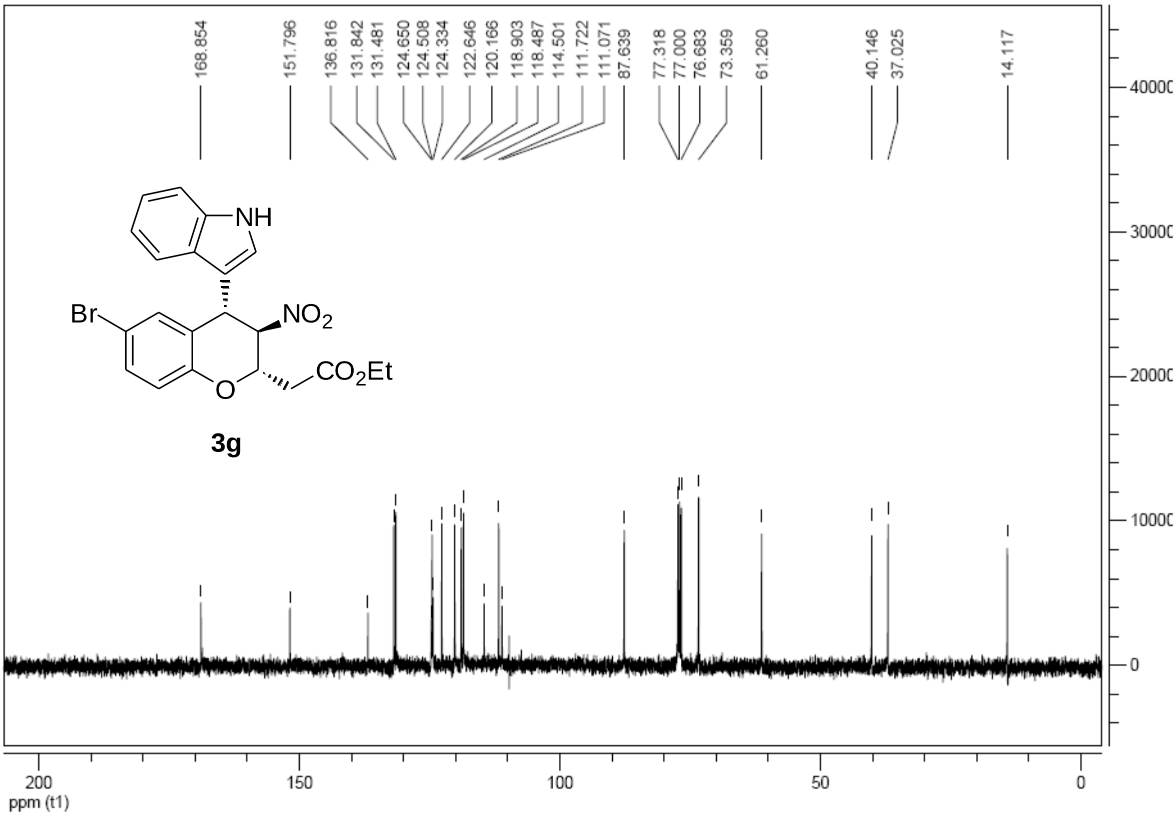

168.854
151.796
136.816
131.842
131.481
124.650
124.508
124.334
122.646
120.166
118.903
118.487
114.501
111.722
111.071
87.639
77.318
77.000
76.683
73.359
61.260
40.146
37.025
14.117
NH
Br
NO2
CO2Et
O
3g
ppm (t1)

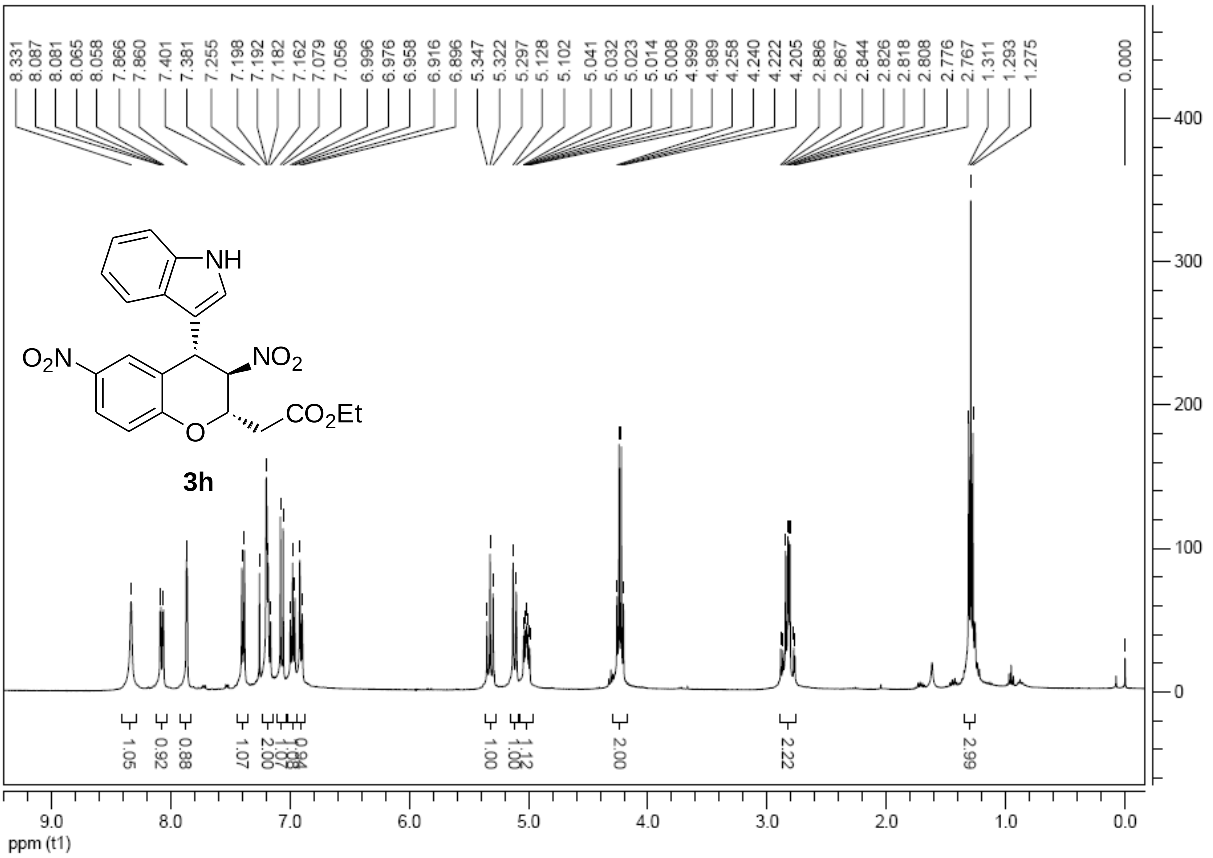

8.331
8.087
8.081
8.065
8.058
7.866
7.860
7.401
7.381
7.255
7.198
7.192
7.182
7.162
7.079
7.056
6.996
6.976
6.958
6.916
6.896
5.347
5.322
5.297
5.128
5.102
5.041
5.032
5.023
5.014
5.008
4.999
4.989
4.258
4.240
4.222
4.205
2.886
2.867
2.844
2.826
2.818
2.808
2.776
2.767
1.311
1.293
1.275
0.000
NH
O_2N
NO_2
CO_2Et
O
3h
1.05
0.92
0.88
1.07
2.00
1.00
1.12
2.00
2.22
2.99
ppm (t1)

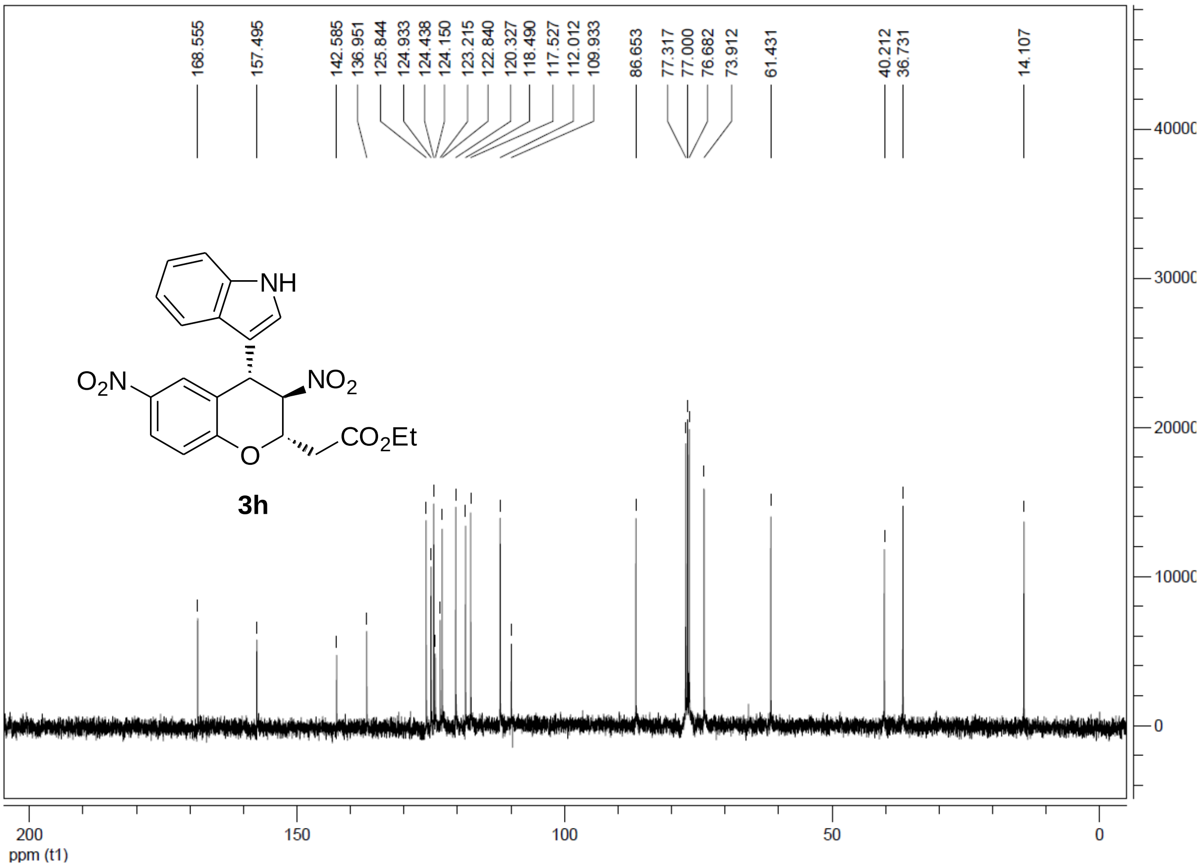

168.555
157.495
142.585
136.951
125.844
124.933
124.438
124.150
123.215
122.840
120.327
118.490
117.527
112.012
109.933
86.653
77.317
77.000
76.682
73.912
61.431
40.212
36.731
14.107
NH
O_2N
NO_2
CO_2Et
O
3h
ppm (t1)

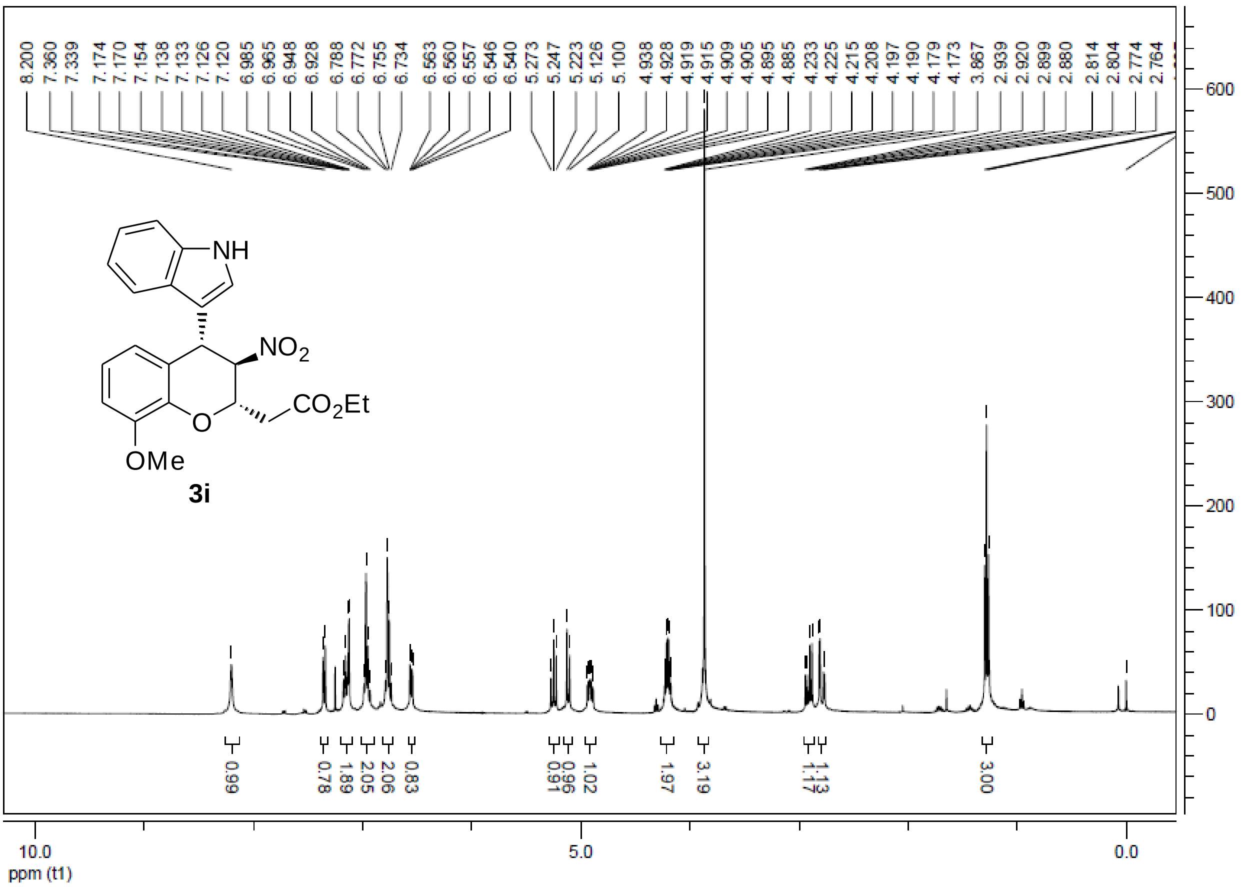

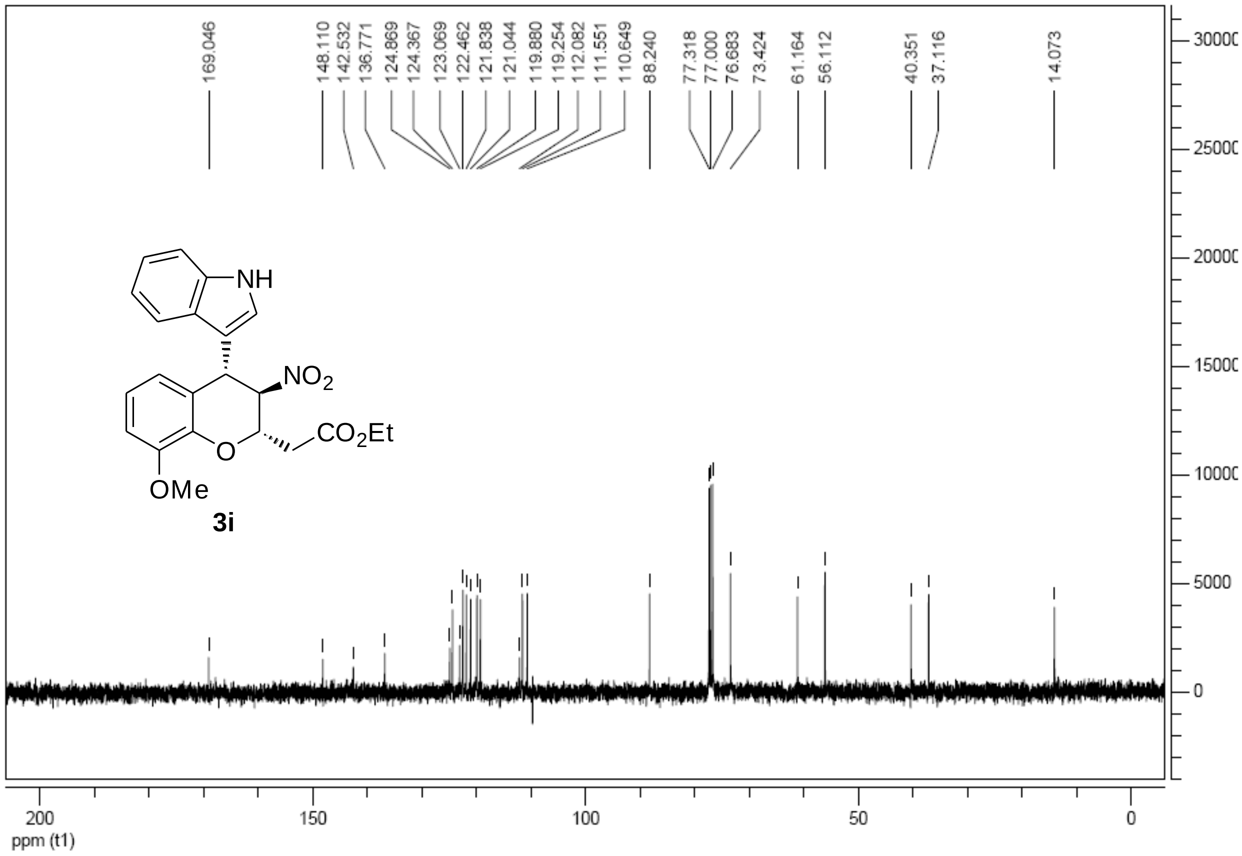

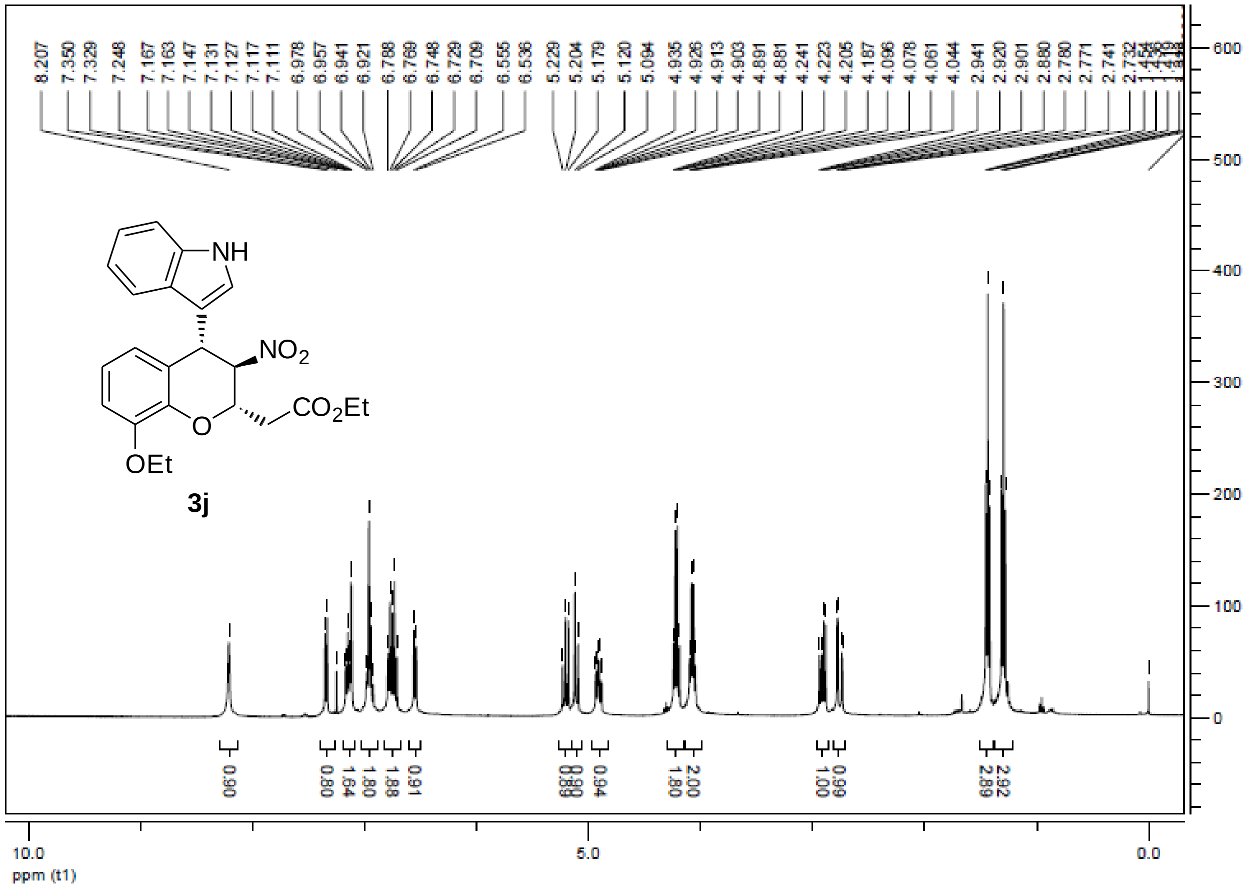

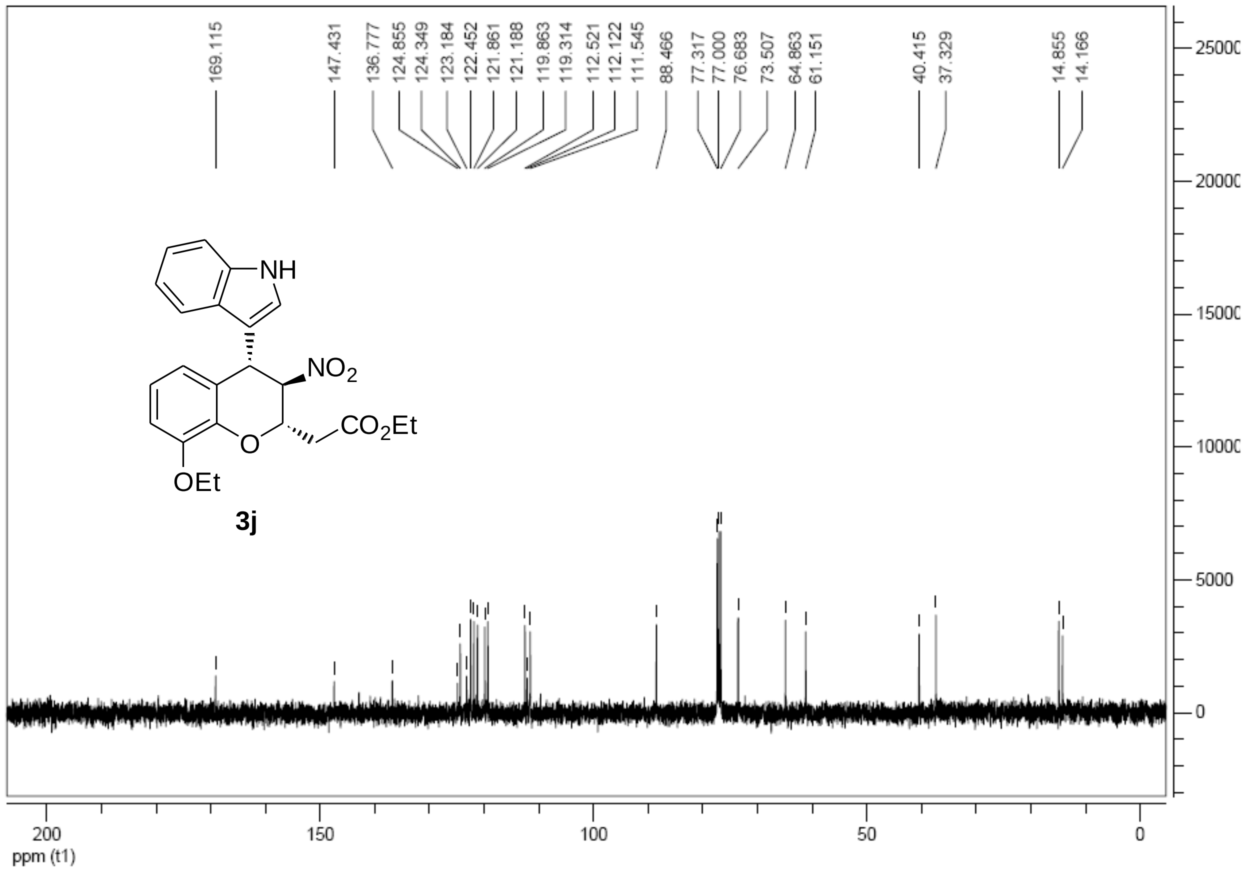

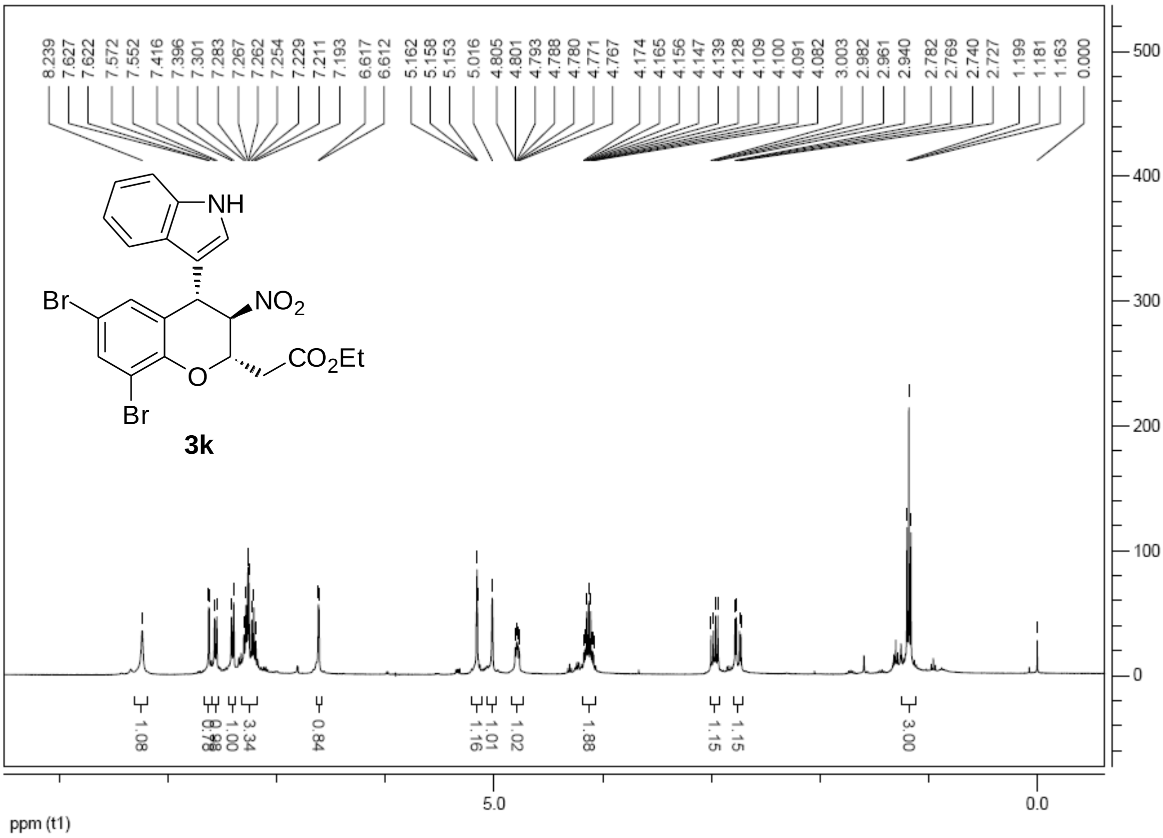
8.239 7.627 7.622 7.572 7.552 7.416 7.396 7.301 7.283 7.267 7.262 7.254 7.229 7.211 7.193 6.617 6.612 5.162 5.158 5.153 5.016 4.805 4.801 4.793 4.788 4.780 4.771 4.767 4.174 4.165 4.156 4.147 4.139 4.128 4.109 4.100 4.091 4.082 3.003 2.982 2.961 2.940 2.782 2.769 2.740 2.727 1.199 1.181 1.163 0.000
NH
Br
NO_2
CO_2Et
O
Br
3k
1.08
1.00
3.34
0.84
1.16
1.01
1.02
1.88
1.15
1.15
3.00
500
400
300
200
100
0
5.0
0.0
ppm (t1)

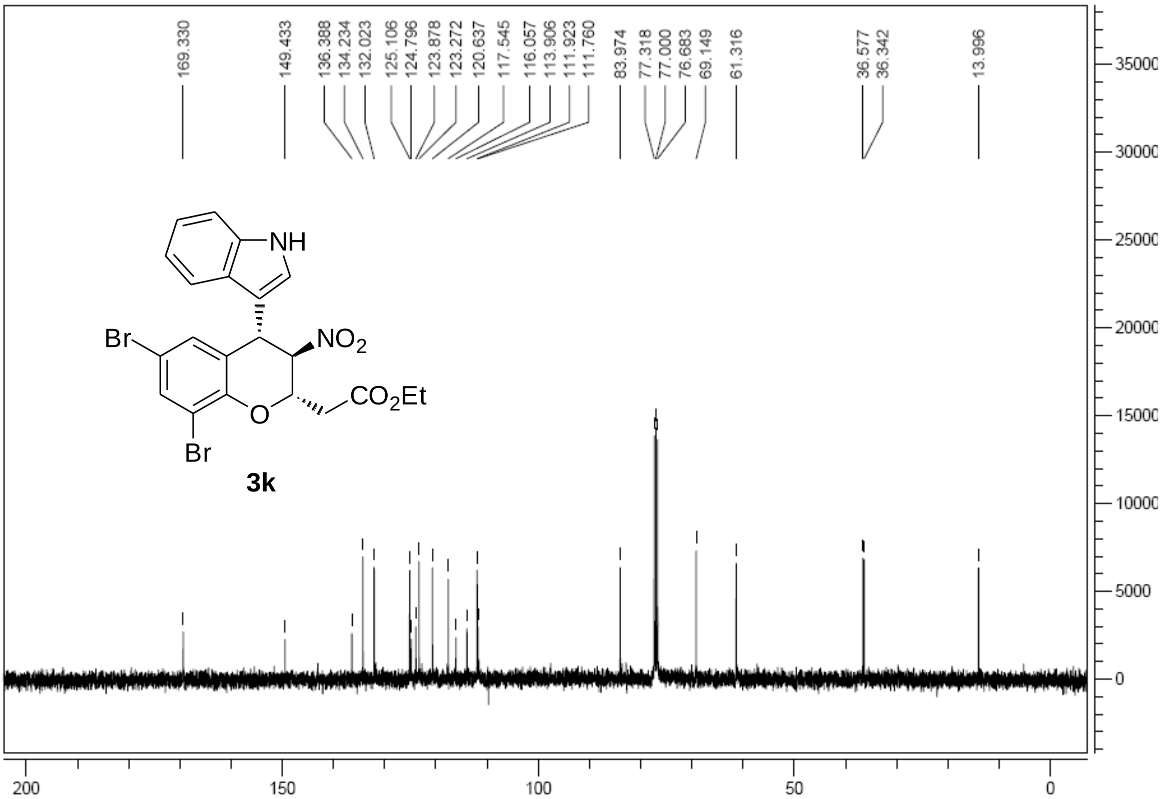
169.330 149.433 136.388 134.234 132.023 125.106 124.796 123.878 123.272 120.637 117.545 116.057 113.906 111.923 111.760 83.974 77.318 77.000 76.683 69.149 61.316 36.577 36.342 13.996
NH
Br
NO_2
CO_2Et
O
Br
3k
35000
30000
25000
20000
15000
10000
5000
0
200
150
100
50
0
ppm (t1)

Copies of HPLC chromatograms of Friedel–Crafts alkylation products 3
HPLC for general procedure A:

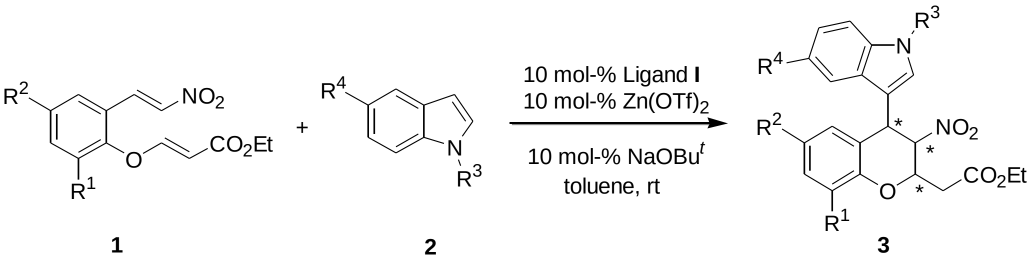

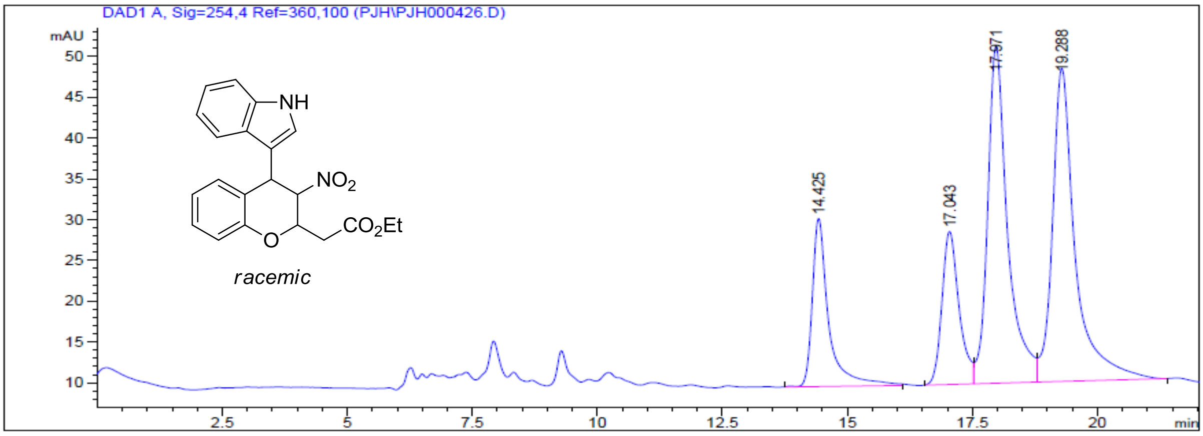

Peak #	RetTime [min]	Type	Width [min]	Area [mAU*s]	Height [mAU]	Area %
1	14.425	BB	0.3348	470.94208	20.59301	14.3463
2	17.043	BV	0.3578	446.39096	18.74902	13.5984
3	17.971	VV	0.4082	1147.83350	41.30601	34.9664
4	19.288	VB	0.4621	1217.51172	38.37741	37.0890

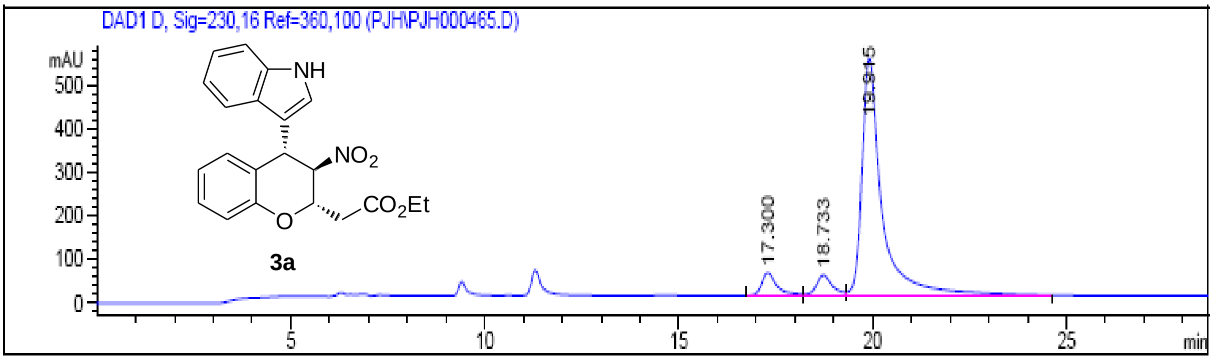

Peak #	RetTime [min]	Type	Width [min]	Area [mAU*s]	Height [mAU]	Area %
1	17.300	BB	0.3808	204.86740	8.05411	4.2582
2	18.733	BV	0.3891	261.34995	10.26371	5.4322
3	19.915	VB	0.4789	4344.92334	132.39555	90.3096

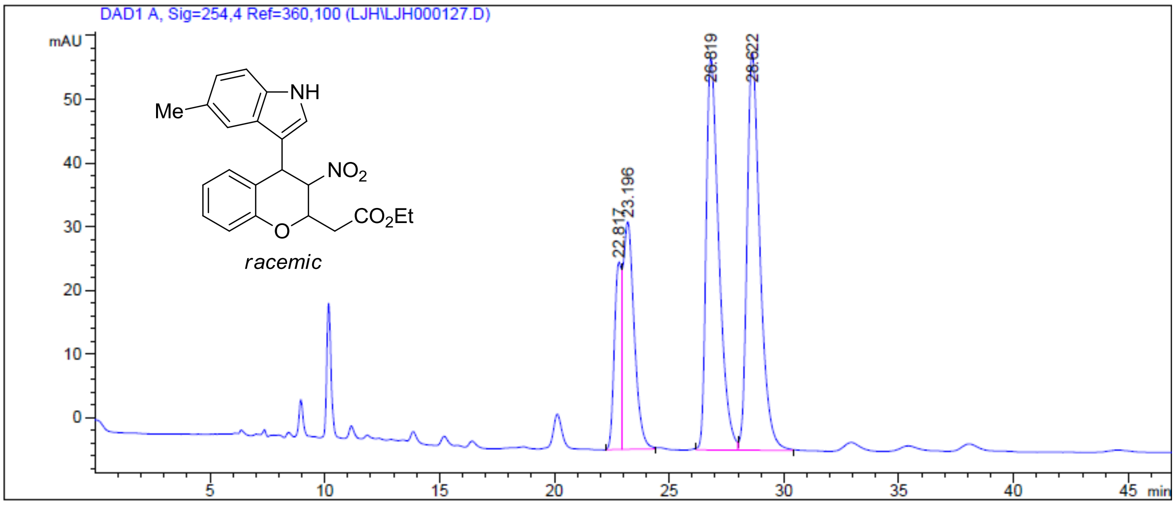

Peak #	RetTime [min]	Type	Width [min]	Area [mAU*s]	Height [mAU]	Area %
1	22.817	BV	0.3231	613.68274	29.45423	9.2343
2	23.196	VB	0.4966	1215.54846	35.75359	18.2908
3	26.819	BV	0.5863	2375.89819	61.55963	35.7511
4	28.622	VB	0.5921	2440.53882	62.42520	36.7238

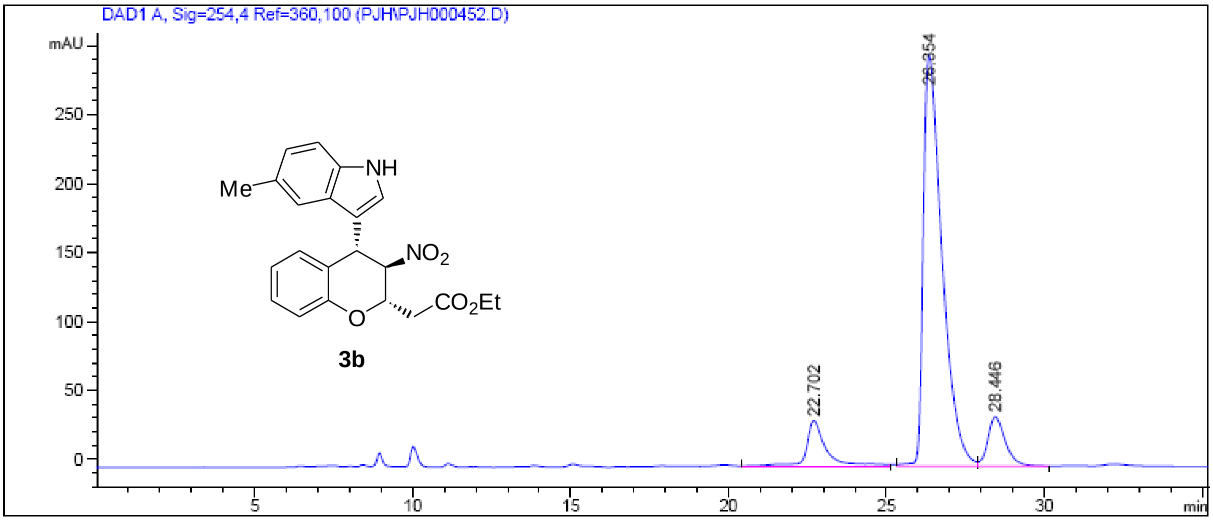

Peak #	RetTime [min]	Type	Width [min]	Area [mAU*s]	Height [mAU]	Area %
1	22.702	VB	0.6327	1497.18750	33.29017	9.8494
2	26.354	BV	0.6173	1.23321e4	298.94305	81.1276
3	28.446	VB	0.5839	1371.57043	35.72448	9.0230

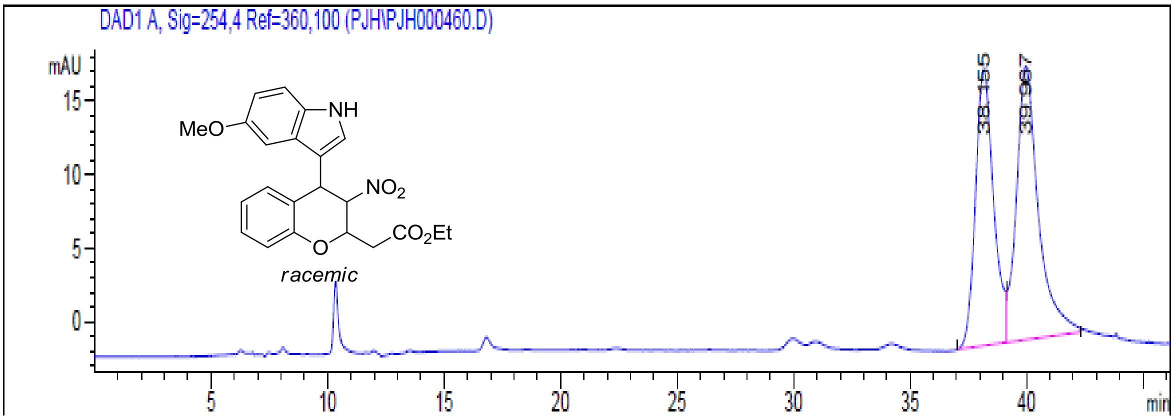

Peak #	RetTime [min]	Type	Width [min]	Area [mAU*s]	Height [mAU]	Area %
1	38.155	BV	0.8164	1017.82837	18.77684	45.2758
2	39.967	VB	0.9472	1230.23523	18.60975	54.7242

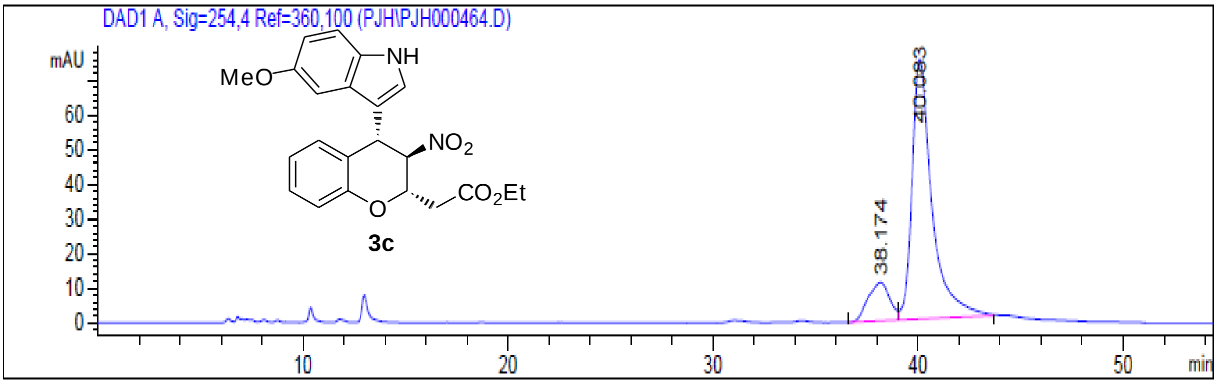

Peak #	RetTime [min]	Type	Width [min]	Area [mAU*s]	Height [mAU]	Area %
1	38.174	BV	0.9971	821.18170	11.23021	13.9044
2	40.083	VB	0.9908	5084.72754	75.01569	86.0956

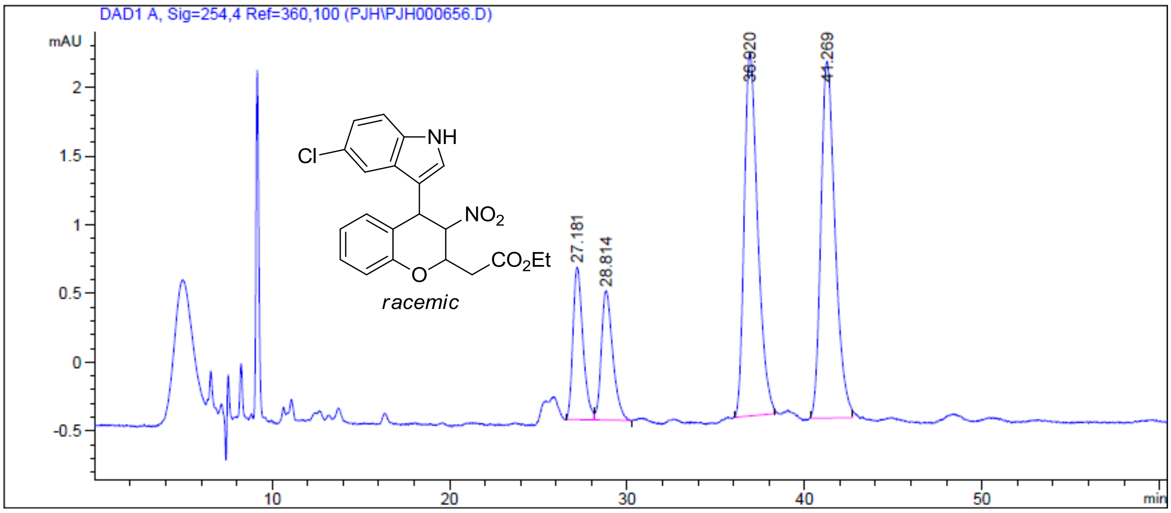

Peak #	RetTime [min]	Type	Width [min]	Area [mAU*s]	Height [mAU]	Area %
1	27.181	BV	0.5521	43.28242	1.10725	11.7468
2	28.814	VB	0.5982	42.10659	9.40729e-1	11.4277
3	36.920	BB	0.7853	138.91309	2.64237	37.7009
4	41.269	BB	0.8198	144.15857	2.59607	39.1245

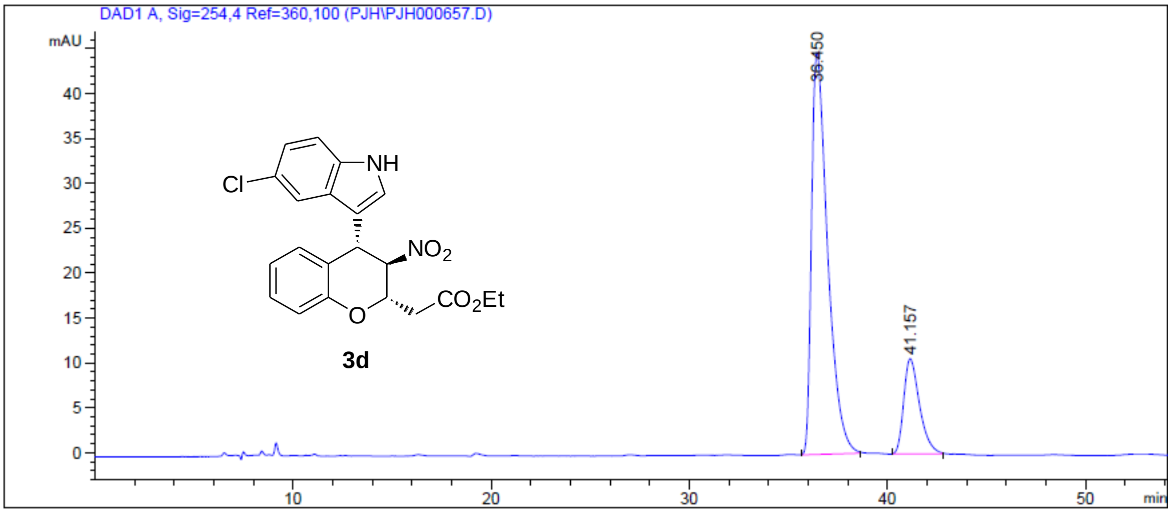

Peak #	RetTime [min]	Type	Width [min]	Area [mAU*s]	Height [mAU]	Area %
1	36.450	BB	0.8414	2522.50000	44.76487	81.0962
2	41.157	BB	0.8193	588.00244	10.59714	18.9038

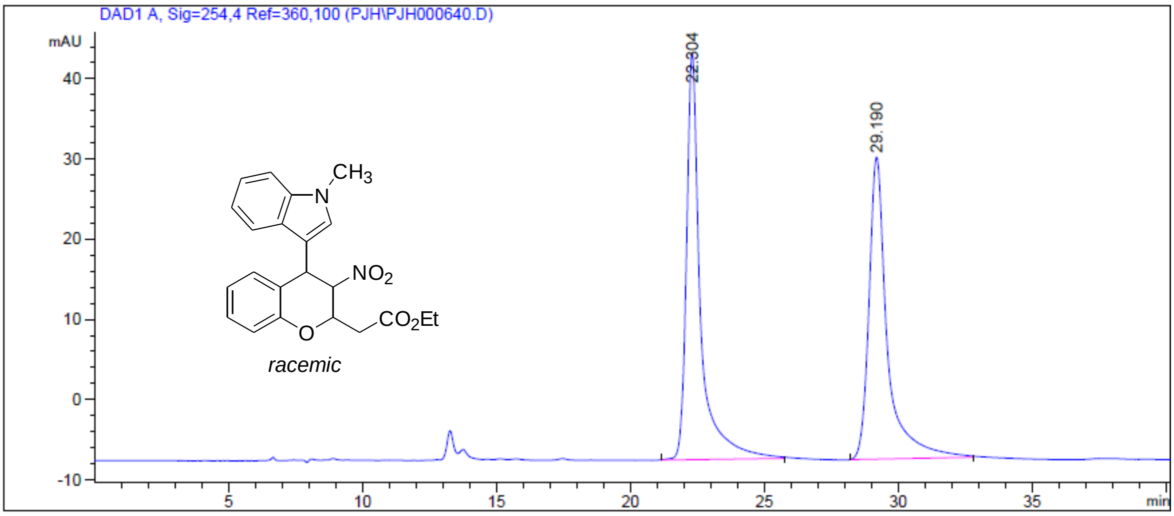

Peak #	RetTime [min]	Type	Width [min]	Area [mAU*s]	Height [mAU]	Area %
1	22.304	BB	0.5271	1856.25916	50.70624	50.7742
2	29.190	BB	0.6971	1799.64893	37.68553	49.2258

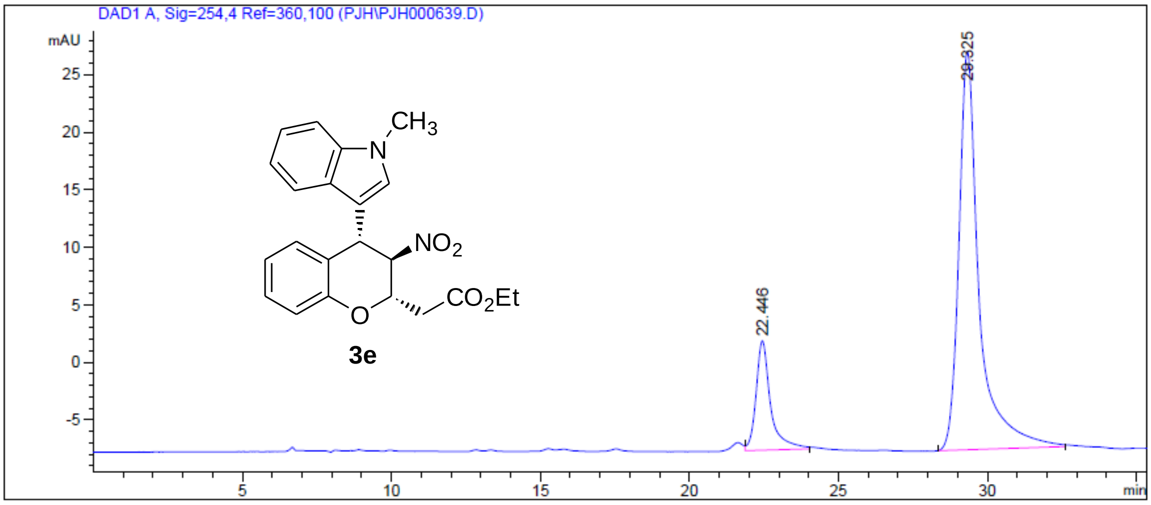

Peak #	RetTime [min]	Type	Width [min]	Area [mAU*s]	Height [mAU]	Area %
1	22.446	VB	0.4973	323.63562	9.50392	16.5062
2	29.325	BB	0.6878	1637.05017	34.61126	83.4938

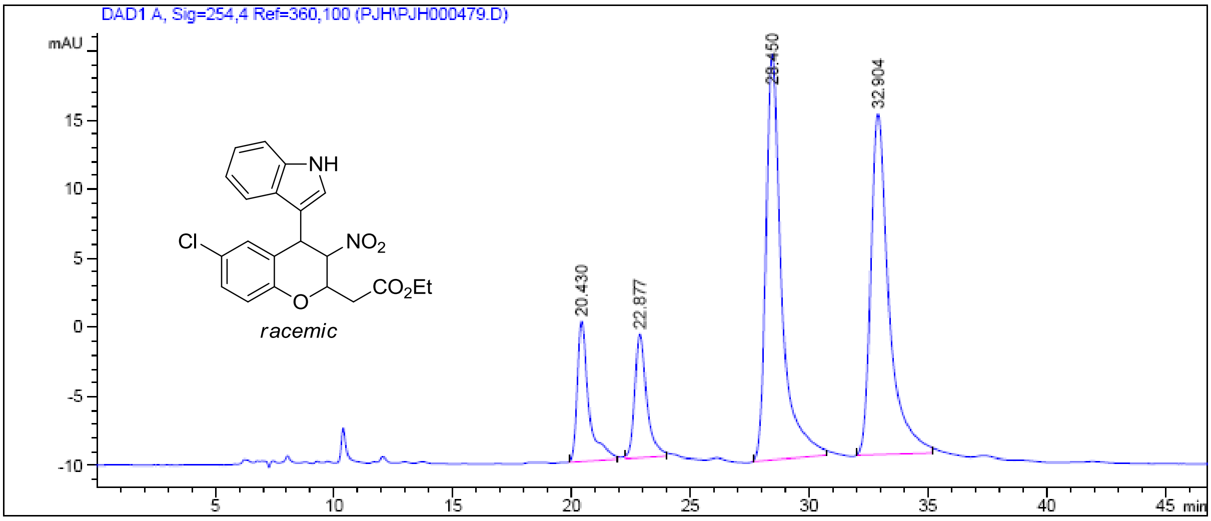

Peak #	RetTime [min]	Type	Width [min]	Area [mAU*s]	Height [mAU]	Area %
1	20.430	BB	0.4955	343.98013	10.14562	10.3510
2	22.877	BB	0.5273	315.23315	8.94243	9.4860
3	28.450	BB	0.6762	1349.34094	29.35835	40.6043
4	32.904	BB	0.7865	1314.59058	24.63610	39.5586

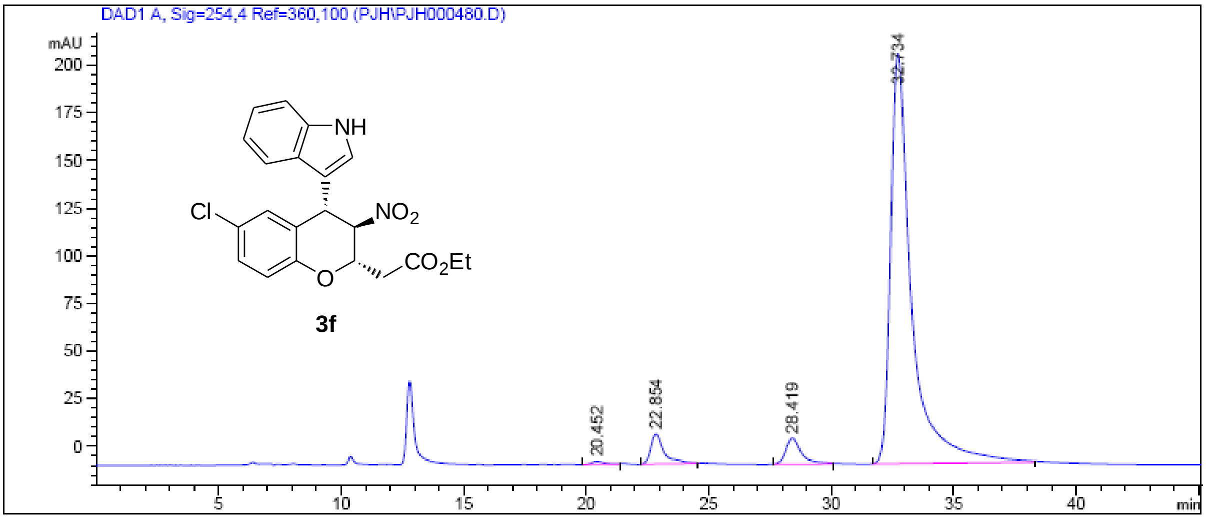

Peak #	RetTime [min]	Type	Width [min]	Area [mAU*s]	Height [mAU]	Area %
1	20.452	BB	0.5064	53.41059	1.58008	0.3784
2	22.854	BB	0.5630	609.21869	15.75852	4.3156
3	28.419	BB	0.6640	610.86908	13.70481	4.3273
4	32.734	BB	0.8688	1.28432e4	215.03745	90.9788

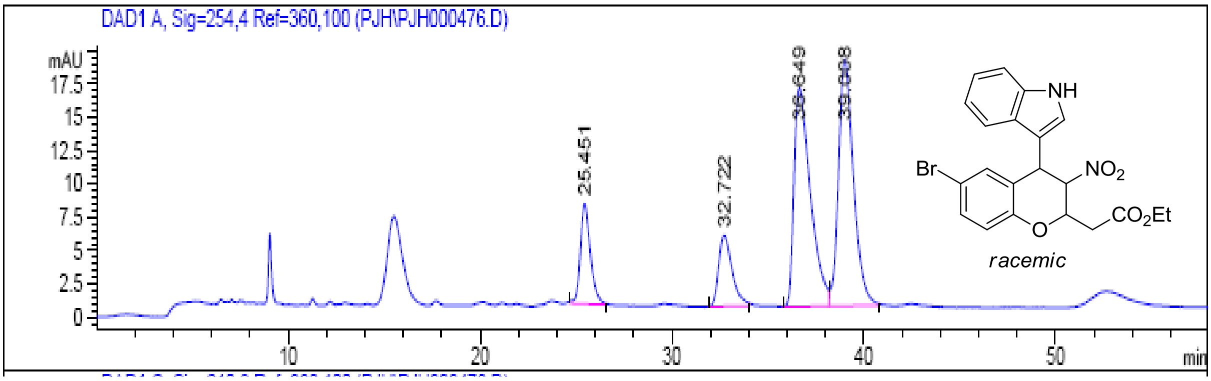

Peak #	RetTime [min]	Type	Width [min]	Area [mAU*s]	Height [mAU]	Area %
1	25.451	BB	0.5600	276.43765	7.53563	10.9628
2	32.722	BB	0.7307	259.29739	5.33794	10.2831
3	36.649	BV	0.8989	979.20367	16.43908	38.8328
4	39.008	VB	0.8221	1006.64923	18.52100	39.9212

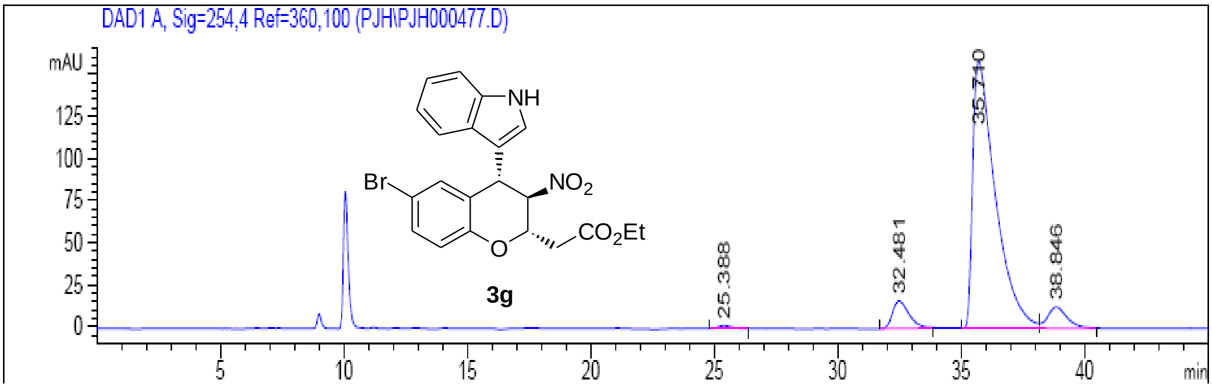

Peak #	RetTime [min]	Type	Width [min]	Area [mAU*s]	Height [mAU]	Area %
1	25.388	BB	0.5063	63.38261	1.76761	0.5450
2	32.481	BB	0.7015	764.64581	16.47381	6.5744
3	35.710	BV	0.9203	1.01006e4	158.32646	86.8442
4	38.846	VB	0.8342	702.08453	12.59846	6.0365

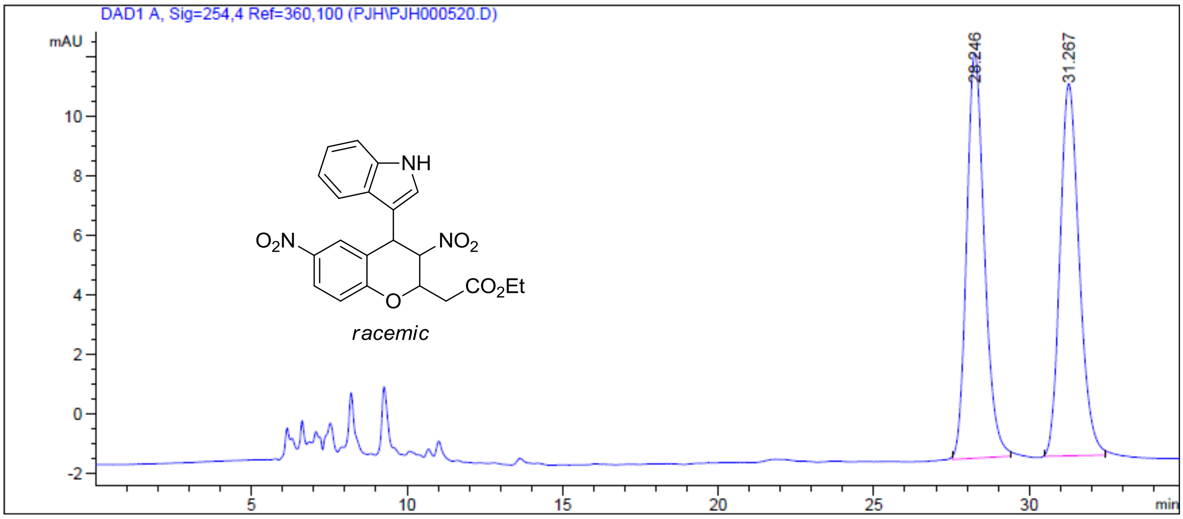

Peak #	RetTime [min]	Type	Width [min]	Area [mAU*s]	Height [mAU]	Area %
1	28.246	BB	0.6059	538.32477	13.59794	50.1577
2	31.267	BB	0.6560	534.94037	12.48315	49.8423

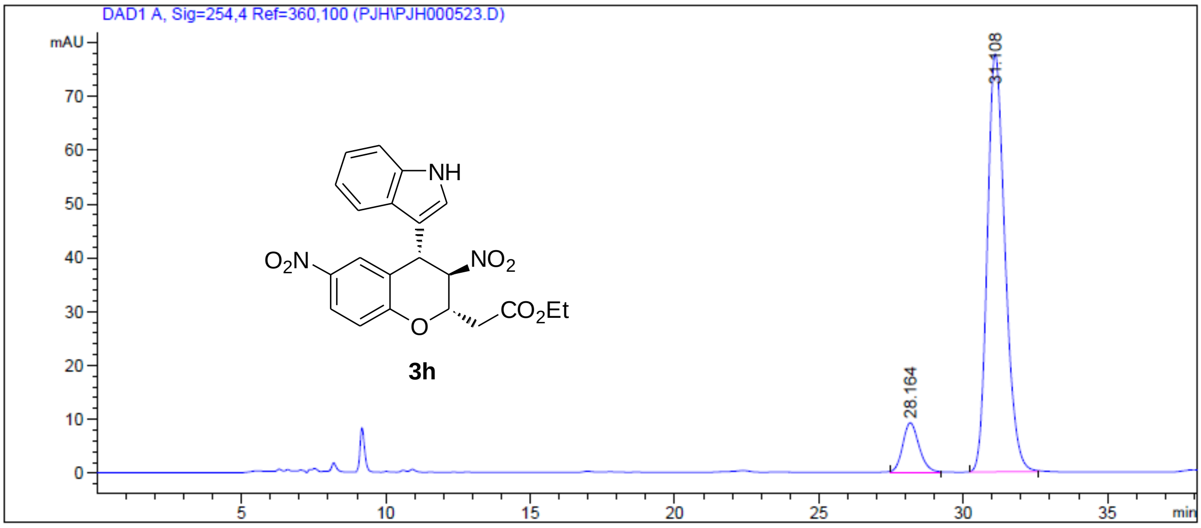

Peak #	RetTime [min]	Type	Width [min]	Area [mAU*s]	Height [mAU]	Area %
1	28.164	BB	0.5974	359.86734	9.17875	9.7714
2	31.108	BB	0.6592	3322.99829	77.65696	90.2286

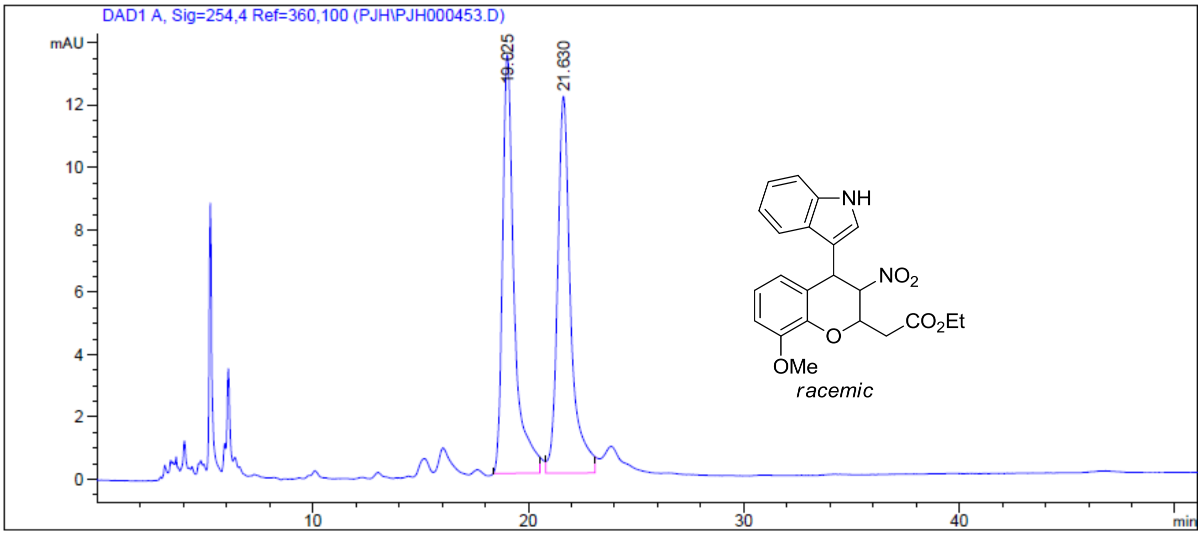

Peak #	RetTime [min]	Type	Width [min]	Area [mAU*s]	Height [mAU]	Area %
1	19.025	BB	0.5542	504.31268	13.42552	49.5421
2	21.630	BB	0.6277	513.63428	12.08769	50.4579

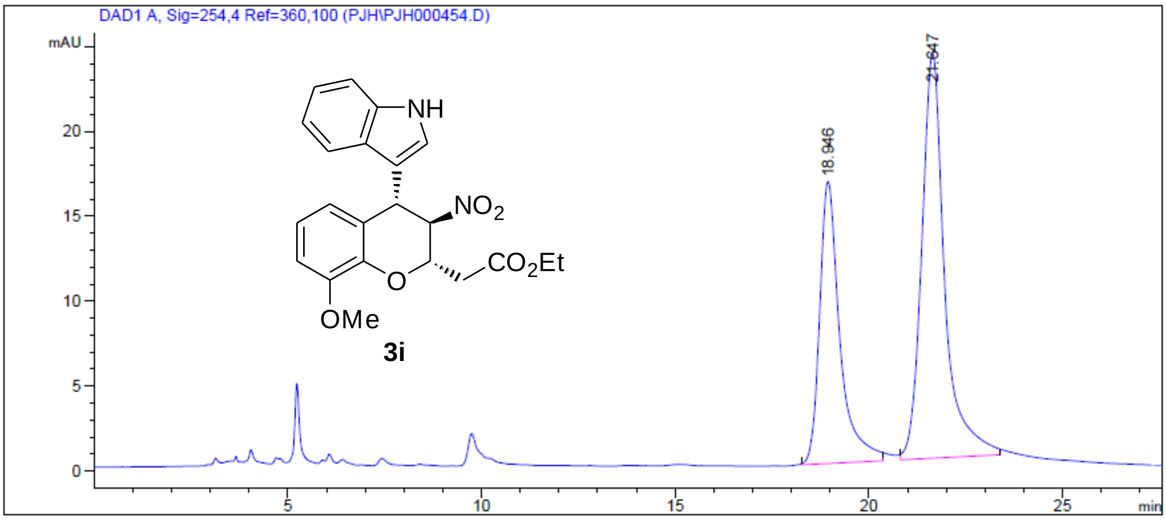

Peak #	RetTime [min]	Type	Width [min]	Area [mAU*s]	Height [mAU]	Area %
1	18.946	BB	0.5387	601.94592	16.61307	37.9814
2	21.647	BB	0.6132	982.89618	23.82914	62.0186

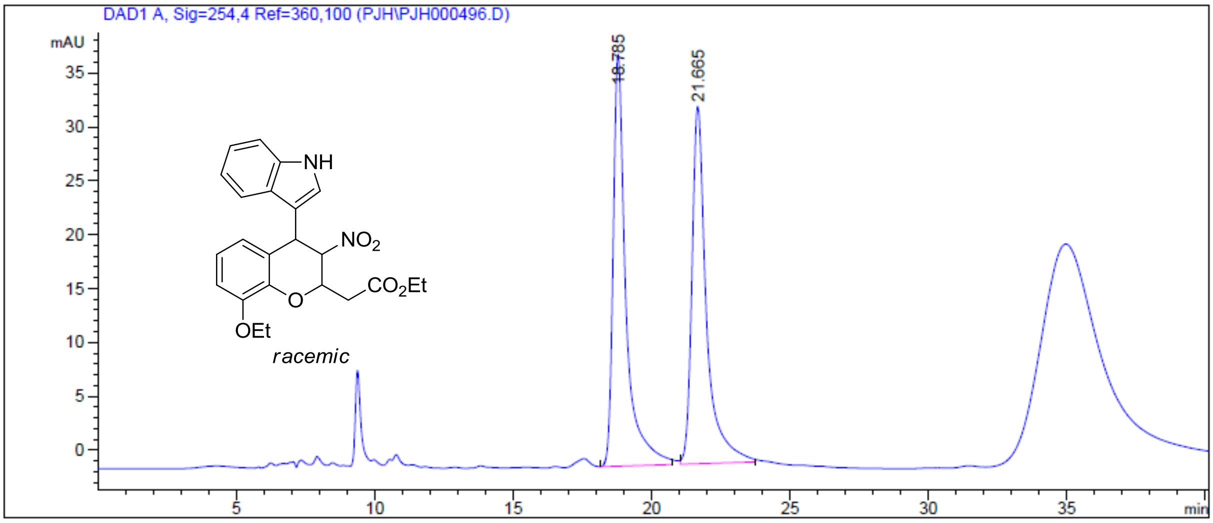

Peak #	RetTime [min]	Type	Width [min]	Area [mAU*s]	Height [mAU]	Area %
1	18.785	VB	0.4666	1227.09937	38.21683	50.3271
2	21.665	BB	0.5338	1211.14795	33.18376	49.6729

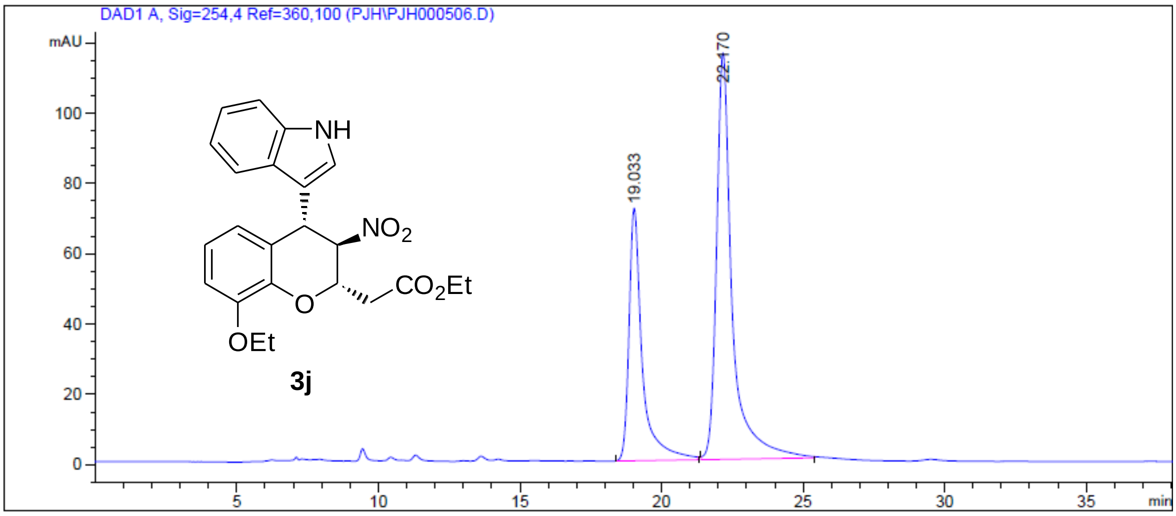

Peak #	RetTime [min]	Type	Width [min]	Area [mAU*s]	Height [mAU]	Area %
1	19.033	BB	0.4772	2345.41577	71.77733	34.5454
2	22.170	BB	0.5608	4443.96289	115.52087	65.4546

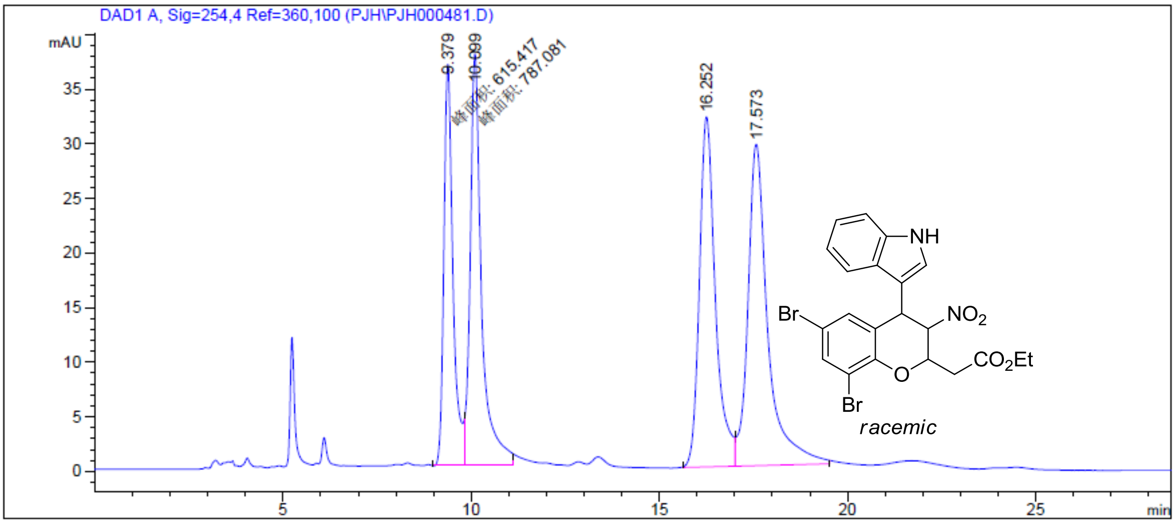

Peak #	RetTime [min]	Type	Width [min]	Area [mAU*s]	Height [mAU]	Area %
1	9.379	MM	0.2796	615.41724	36.67808	17.9115
2	10.099	MM	0.3484	787.08063	37.64749	22.9077
3	16.252	BV	0.4450	948.49451	32.07032	27.6056
4	17.573	VB	0.5379	1084.88025	29.44399	31.5751

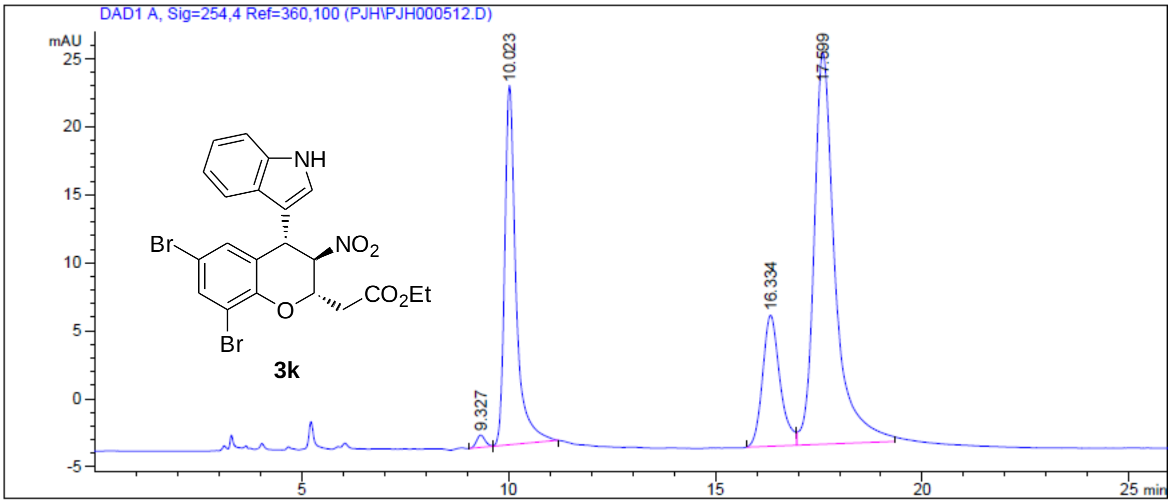

Peak #	RetTime [min]	Type	Width [min]	Area [mAU*s]	Height [mAU]	Area %
1	9.327	BV S	0.2200	12.97383	9.01080e-1	0.7131
2	10.023	VB S	0.2824	503.13354	26.39430	27.6550
3	16.334	BV	0.4416	281.95447	9.62871	15.4978
4	17.599	VB	0.5259	1021.25946	28.78873	56.1341

HPLC for general procedure B:

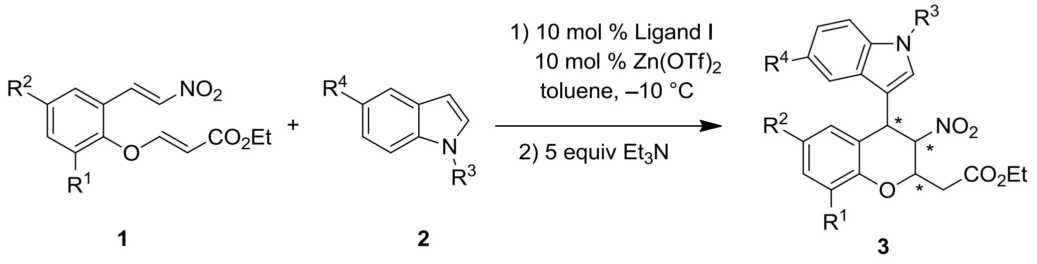

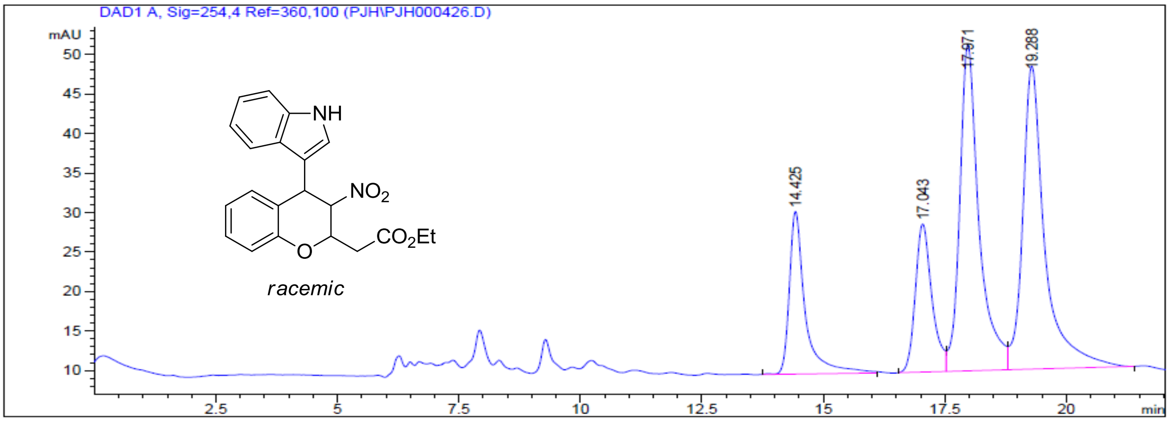

Peak #	RetTime [min]	Type	Width [min]	Area [mAU*s]	Height [mAU]	Area %
1	14.425	BB	0.3348	470.94208	20.59301	14.3463
2	17.043	BV	0.3578	446.39096	18.74902	13.5984
3	17.971	VV	0.4082	1147.83350	41.30601	34.9664
4	19.288	VB	0.4621	1217.51172	38.37741	37.0890

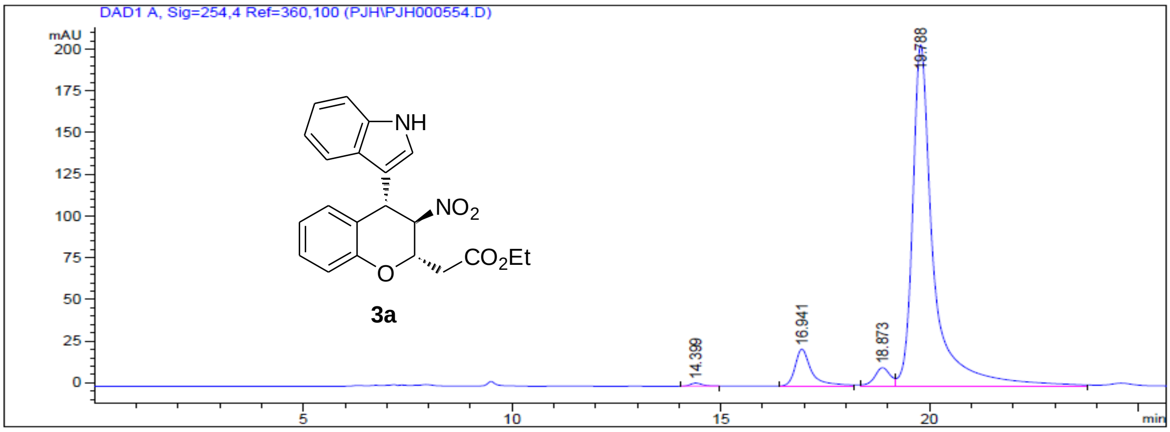

Peak #	RetTime [min]	Type	Width [min]	Area [mAU*s]	Height [mAU]	Area %
1	14.399	BB	0.3060	37.47469	1.83684	0.4644
2	16.941	BB	0.4024	612.50964	22.16479	7.5908
3	18.873	BV	0.3882	288.42459	11.06204	3.5744
4	19.788	VB	0.5028	7130.74170	204.49789	88.3704

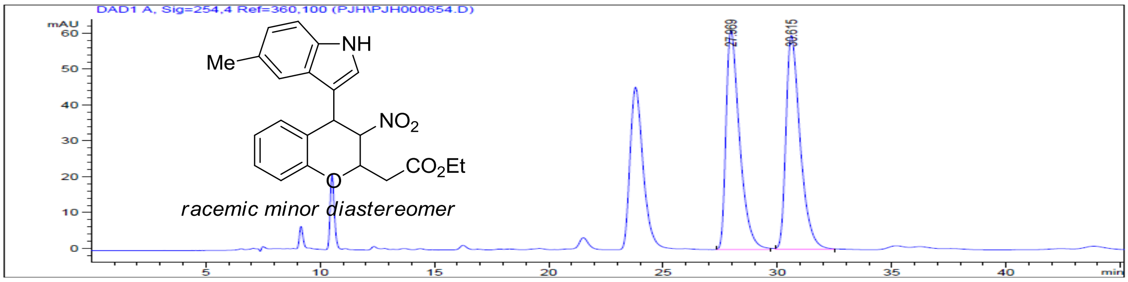

Peak #	RetTime [min]	Type	Width [min]	Area [mAU*s]	Height [mAU]	Area %
1	27.969	BB	0.6207	2516.91699	61.08672	49.3982
2	30.615	BB	0.6520	2578.23779	59.68681	50.6018

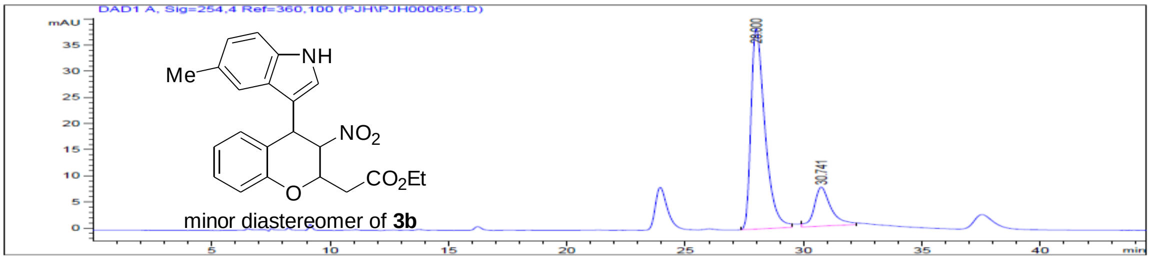

Peak #	RetTime [min]	Type	Width [min]	Area [mAU*s]	Height [mAU]	Area %
1	28.000	BB	0.6187	1573.56995	38.34930	81.0979
2	30.741	BB	0.7213	366.76266	7.46397	18.9021

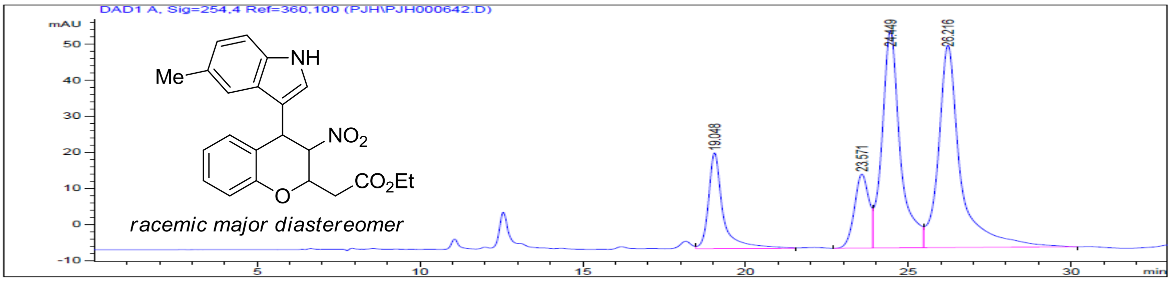

Peak #	RetTime [min]	Type	Width [min]	Area [mAU*s]	Height [mAU]	Area %
1	19.048	VB	0.4509	825.65344	26.54324	12.7752
2	23.571	VV	0.4725	640.87122	20.51011	9.9161
3	24.449	VV	0.5700	2344.78638	60.26061	36.2806
4	26.216	VB	0.6755	2651.61792	56.09292	41.0281

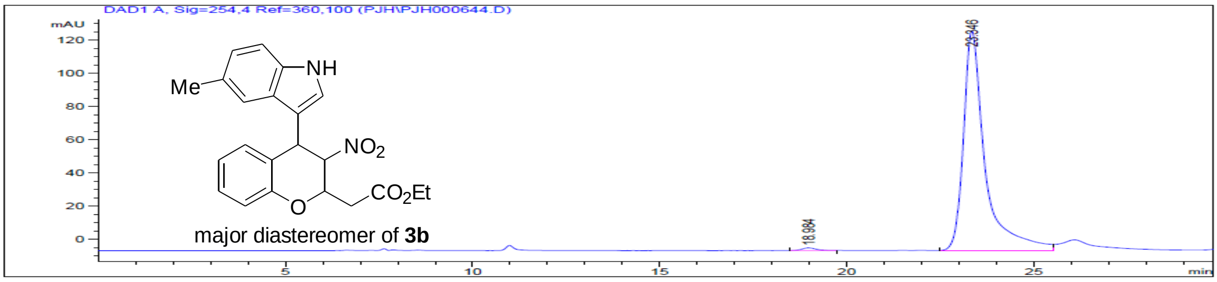

Peak #	RetTime [min]	Type	Width [min]	Area [mAU*s]	Height [mAU]	Area %
1	18.984	BB	0.4179	44.85406	1.62584	0.8390
2	23.346	BV	0.5798	5301.20850	132.19774	99.1610

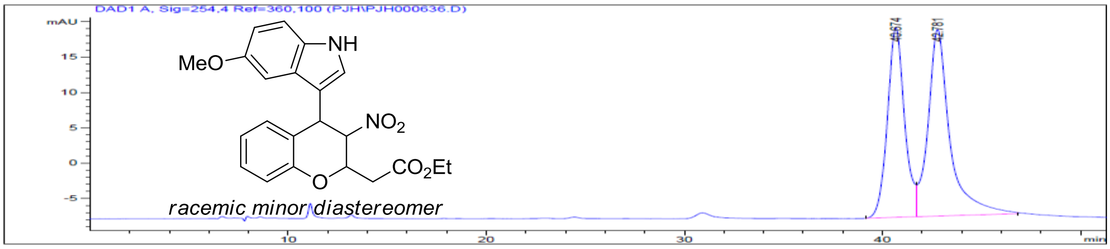

Peak #	RetTime [min]	Type	Width [min]	Area [mAU*s]	Height [mAU]	Area %
1	40.674	BV	0.9110	1618.65234	26.86332	44.3716
2	42.781	VB	1.1007	2029.29077	26.40225	55.6284

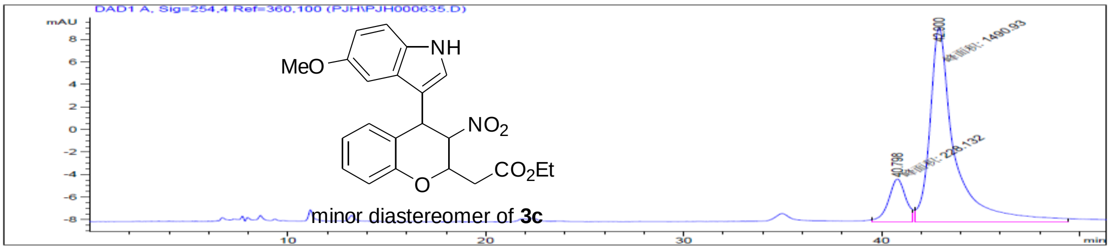

Peak #	RetTime [min]	Type	Width [min]	Area [mAU*s]	Height [mAU]	Area %
1	40.798	MM	1.0038	228.13240	3.78784	13.2707
2	42.900	MM	1.4420	1490.93433	17.23179	86.7293

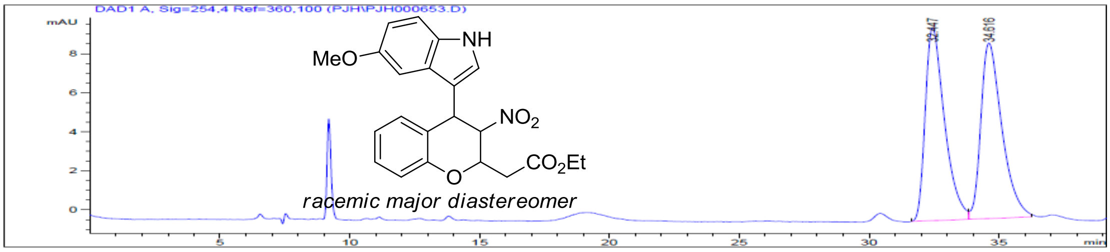

Peak #	RetTime [min]	Type	Width [min]	Area [mAU*s]	Height [mAU]	Area %
1	32.447	BV	0.7725	511.92172	9.87965	50.1833
2	34.616	VB	0.8355	508.18140	8.99018	49.8167

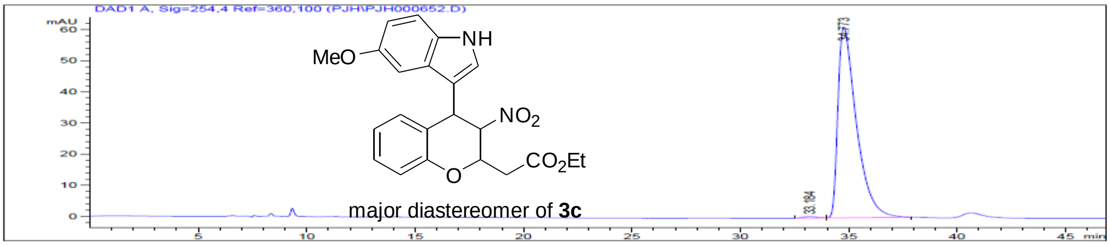

Peak #	RetTime [min]	Type	Width [min]	Area [mAU*s]	Height [mAU]	Area %
1	33.184	BV	0.5506	23.04489	5.00999e-1	0.6309
2	34.773	VB	0.8853	3629.72974	61.26010	99.3691

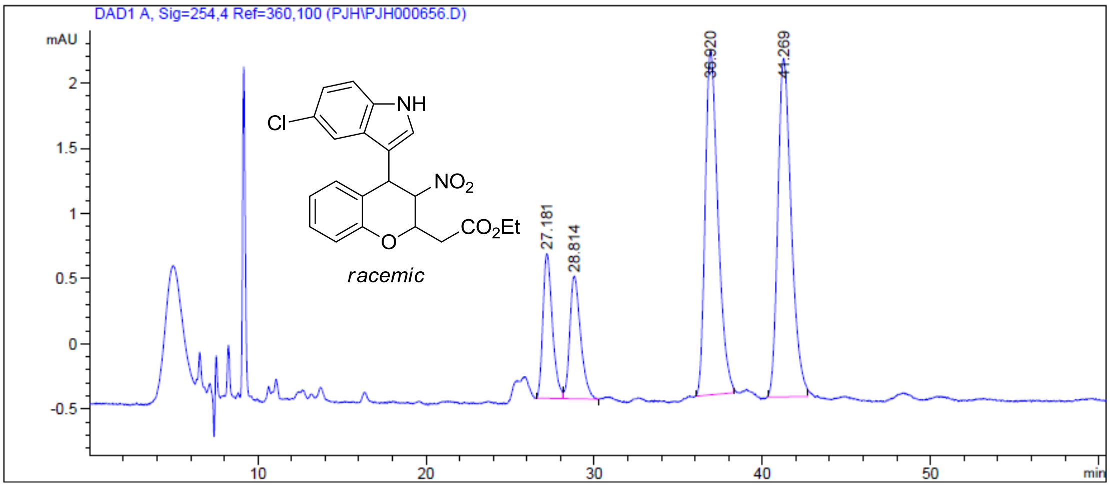

Peak #	RetTime [min]	Type	Width [min]	Area [mAU*s]	Height [mAU]	Area %
1	27.181	BV	0.5521	43.28242	1.10725	11.7468
2	28.814	VB	0.5982	42.10659	9.40729e-1	11.4277
3	36.920	BB	0.7853	138.91309	2.64237	37.7009
4	41.269	BB	0.8198	144.15857	2.59607	39.1245

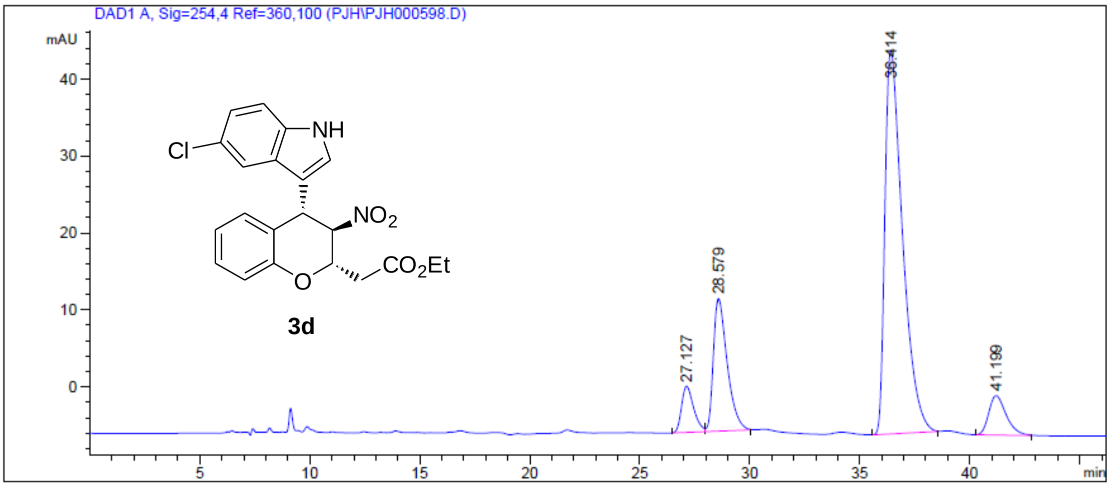

Peak #	RetTime [min]	Type	Width [min]	Area [mAU*s]	Height [mAU]	Area %
1	27.127	BV	0.5724	228.35405	5.99471	5.6419
2	28.579	VB	0.6583	753.54620	17.22746	18.6176
3	36.414	BB	0.8476	2783.51733	49.84023	68.7715
4	41.199	BB	0.8107	282.07034	5.12024	6.9690

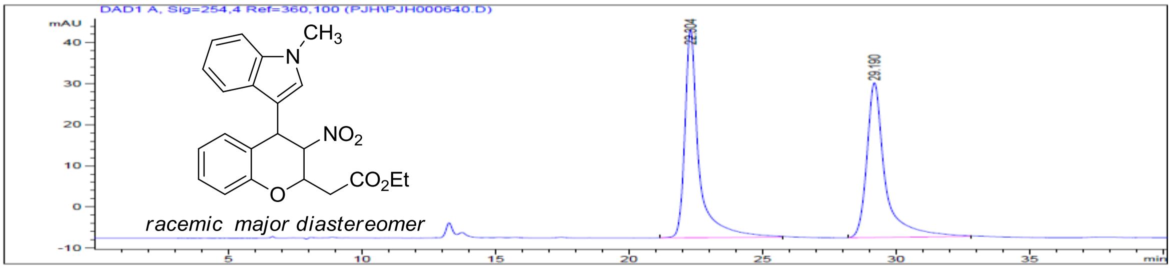

Peak #	RetTime [min]	Type	Width [min]	Area [mAU*s]	Height [mAU]	Area %
1	22.304	BB	0.5271	1856.25916	50.70624	50.7742
2	29.190	BB	0.6971	1799.64893	37.68553	49.2258

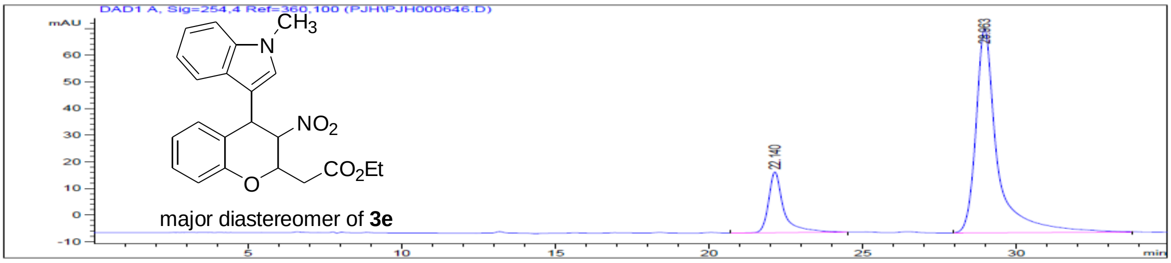

Peak #	RetTime [min]	Type	Width [min]	Area [mAU*s]	Height [mAU]	Area %
1	22.140	VB	0.5027	786.53900	22.78428	17.4382
2	28.963	BB	0.7042	3723.89355	76.46532	82.5618

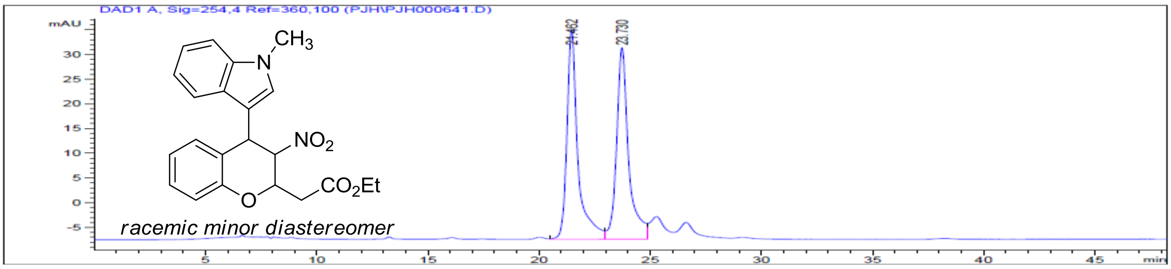

Peak #	RetTime [min]	Type	Width [min]	Area [mAU*s]	Height [mAU]	Area %
1	21.462	VV	0.4957	1452.49719	42.39354	49.9138
2	23.730	VV	0.5507	1457.51196	38.76280	50.0862

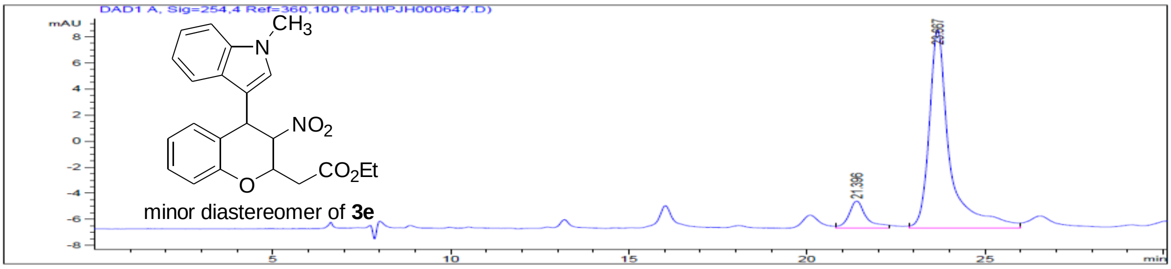

Peak #	RetTime [min]	Type	Width [min]	Area [mAU*s]	Height [mAU]	Area %
1	21.396	VB	0.4954	70.22806	2.07194	10.3365
2	23.667	BB	0.5745	609.18811	15.23444	89.6635

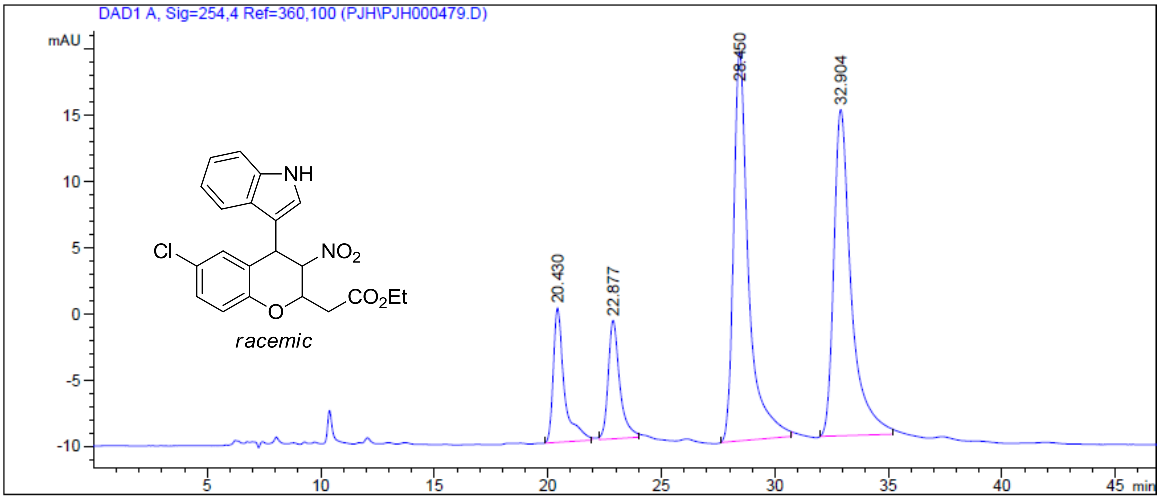

Peak #	RetTime [min]	Type	Width [min]	Area [mAU*s]	Height [mAU]	Area %
1	20.430	BB	0.4955	343.98013	10.14562	10.3510
2	22.877	BB	0.5273	315.23315	8.94243	9.4860
3	28.450	BB	0.6762	1349.34094	29.35835	40.6043
4	32.904	BB	0.7865	1314.59058	24.63610	39.5586

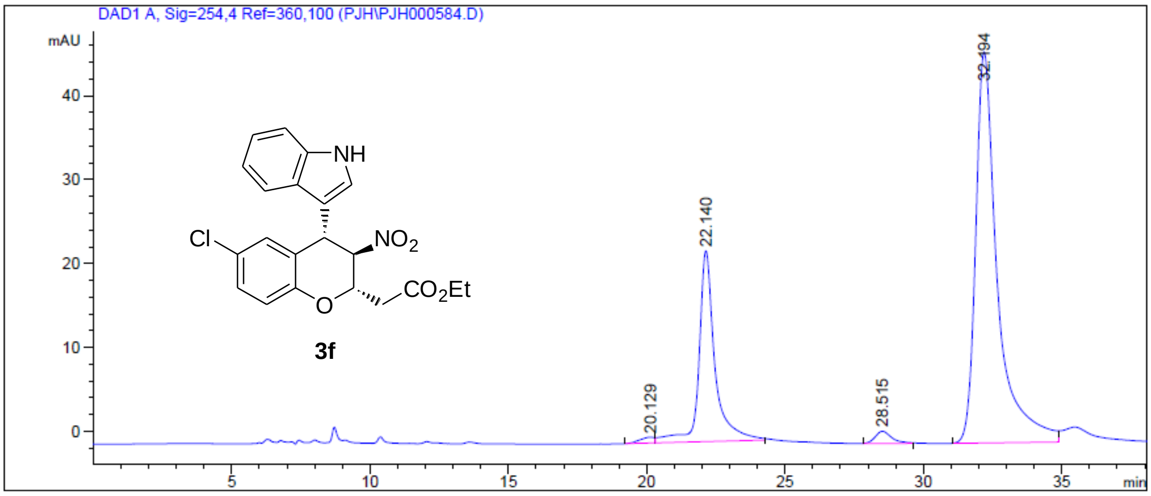

Peak #	RetTime [min]	Type	Width [min]	Area [mAU*s]	Height [mAU]	Area %
1	20.129	BV	0.4537	24.46895	6.83407e-1	0.6895
2	22.140	VB	0.5822	915.72150	22.72285	25.8042
3	28.515	BB	0.6125	59.56060	1.44629	1.6784
4	32.194	BB	0.8024	2548.98413	46.58105	71.8280

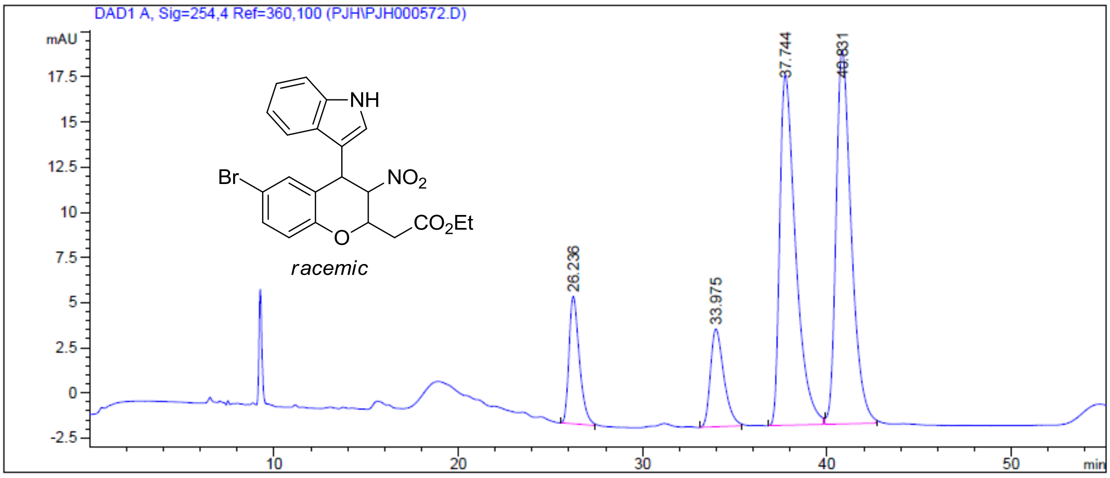

Peak #	RetTime [min]	Type	Width [min]	Area [mAU*s]	Height [mAU]	Area %
1	26.236	BB	0.5927	276.12054	7.05343	9.4728
2	33.975	BB	0.7685	275.09558	5.41643	9.4377
3	37.744	BB	0.8948	1169.99939	19.42315	40.1390
4	40.831	BB	0.8686	1193.65100	20.70538	40.9505

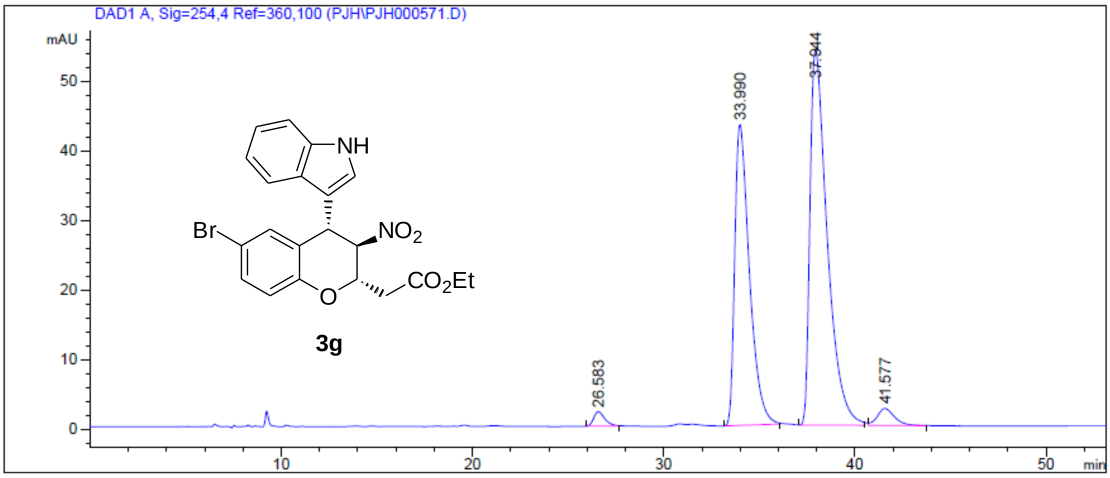

Peak #	RetTime [min]	Type	Width [min]	Area [mAU*s]	Height [mAU]	Area %
1	26.583	BB	0.5809	84.89101	2.09442	1.4342
2	33.990	BB	0.8011	2304.80957	43.29633	38.9378
3	37.944	BB	0.9349	3375.88086	53.88437	57.0327
4	41.577	BB	0.8428	153.62460	2.43924	2.5954

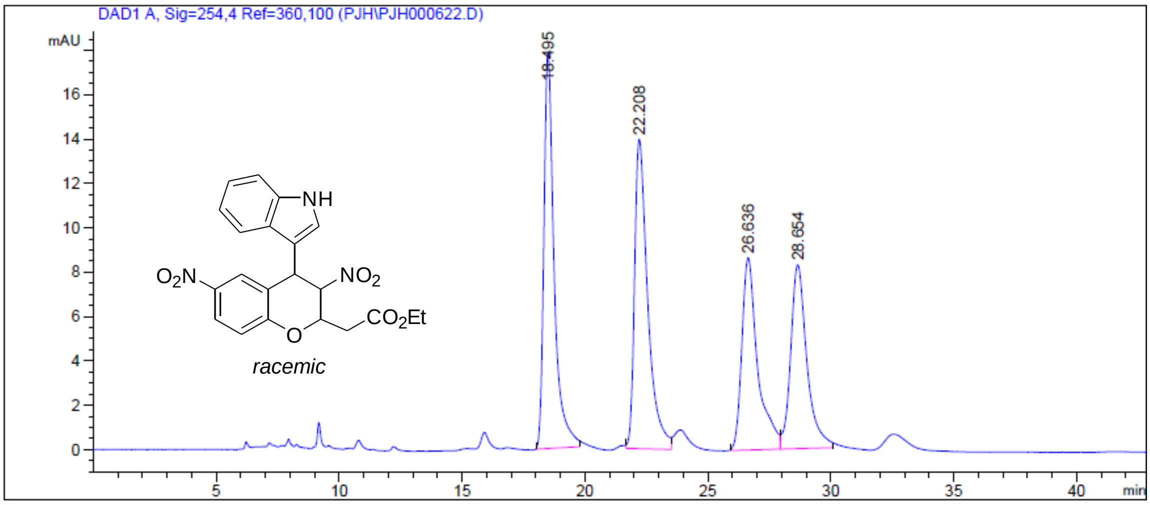

Peak #	RetTime [min]	Type	Width [min]	Area [mAU*s]	Height [mAU]	Area %
1	18.495	BB	0.4285	515.27405	17.86629	28.5916
2	22.208	BB	0.5542	518.97247	13.94456	28.7968
3	26.636	BV	0.6630	388.64386	8.66936	21.5651
4	28.654	VB	0.6781	379.29935	8.28535	21.0466

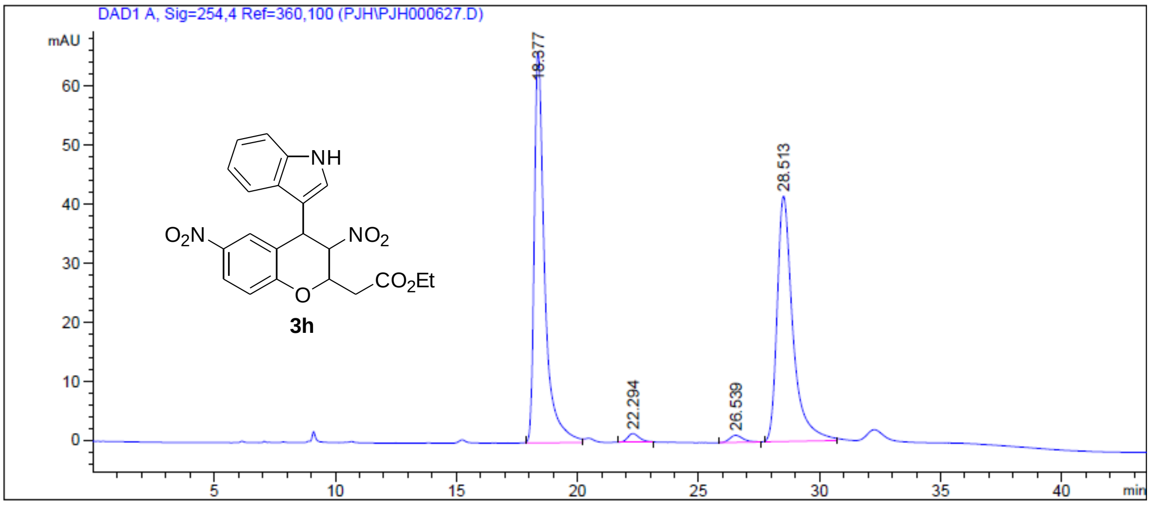

Peak #	RetTime [min]	Type	Width [min]	Area [mAU*s]	Height [mAU]	Area %
1	18.377	BB	0.4403	1977.49097	66.24683	50.0395
2	22.294	BB	0.5321	51.97419	1.48585	1.3152
3	26.539	BB	0.5969	46.21715	1.20120	1.1695
4	28.513	BB	0.6747	1876.17468	41.56017	47.4758

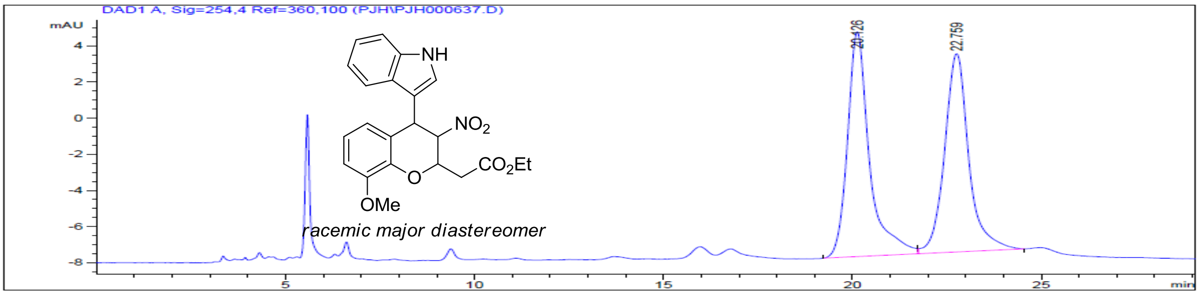

Peak #	RetTime [min]	Type	Width [min]	Area [mAU*s]	Height [mAU]	Area %
1	20.126	BV	0.5909	501.79147	12.43117	50.6645
2	22.759	VB	0.6634	488.62848	10.97617	49.3355

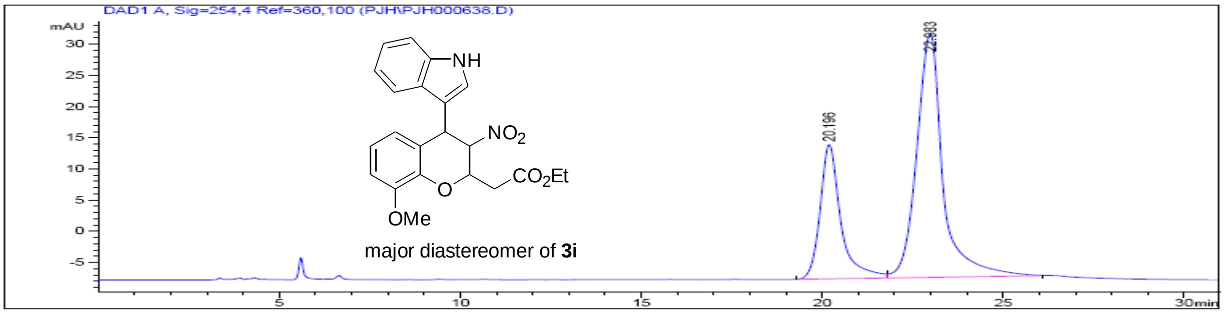

Peak #	RetTime [min]	Type	Width [min]	Area [mAU*s]	Height [mAU]	Area %
1	20.196	BV	0.5874	853.76044	21.49254	30.3346
2	22.983	VB	0.7503	1960.71924	39.01451	69.6654

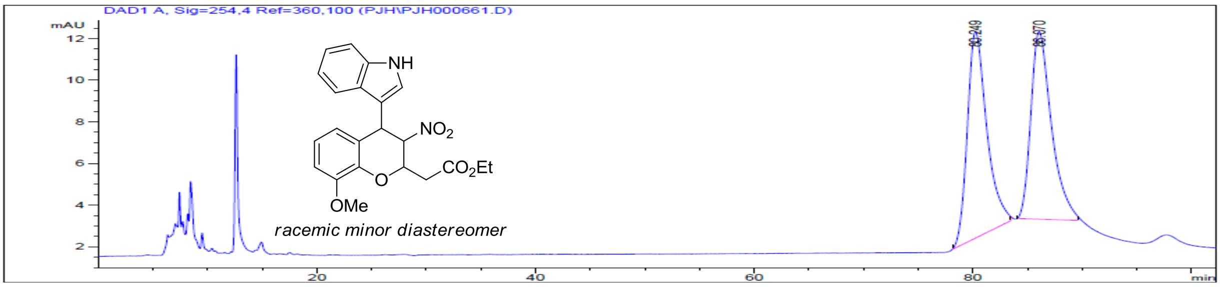

Peak #	RetTime [min]	Type	Width [min]	Area [mAU*s]	Height [mAU]	Area %
1	80.249	BB	1.6820	1227.86475	9.90668	50.9418
2	86.070	BB	1.6936	1182.46167	9.01890	49.0582

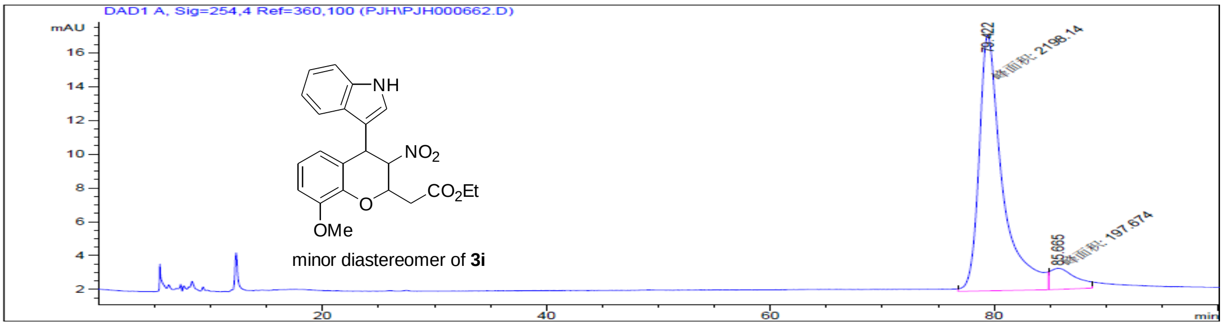

Peak #	RetTime [min]	Type	Width [min]	Area [mAU*s]	Height [mAU]	Area %
1	79.422	MM	2.4190	2198.14111	15.14480	91.7492
2	85.665	MM	2.6341	197.67389	1.25071	8.2508

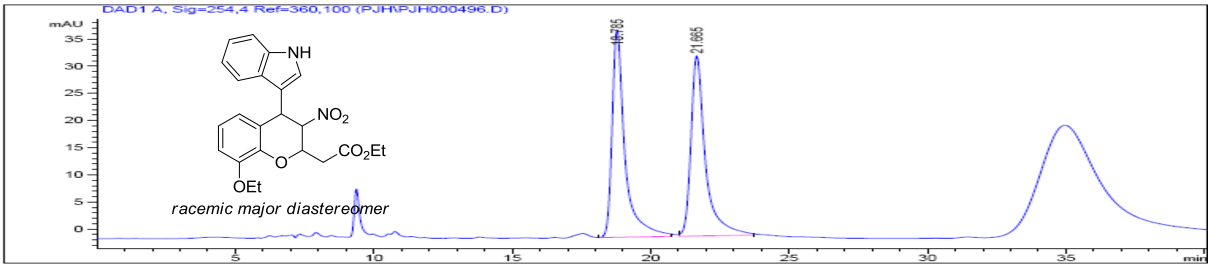

Peak #	RetTime [min]	Type	Width [min]	Area [mAU*s]	Height [mAU]	Area %
1	18.785	VB	0.4666	1227.09937	38.21683	50.3271
2	21.665	BB	0.5338	1211.14795	33.18376	49.6729

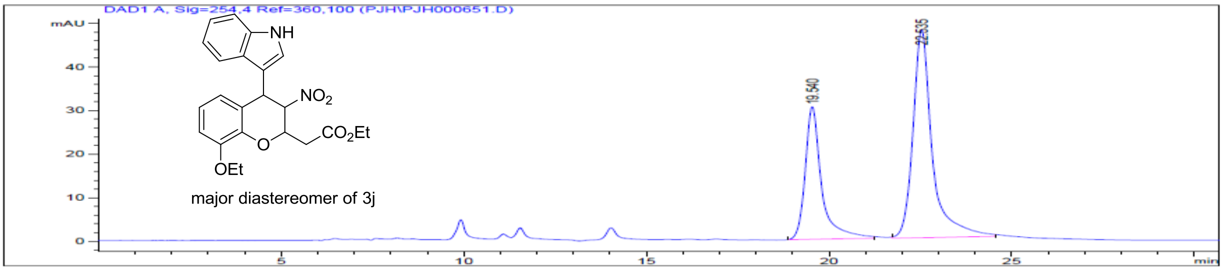

Peak #	RetTime [min]	Type	Width [min]	Area [mAU*s]	Height [mAU]	Area %
1	19.540	BB	0.4573	951.42200	30.38398	35.2151
2	22.535	BB	0.5406	1750.32532	47.64565	64.7849

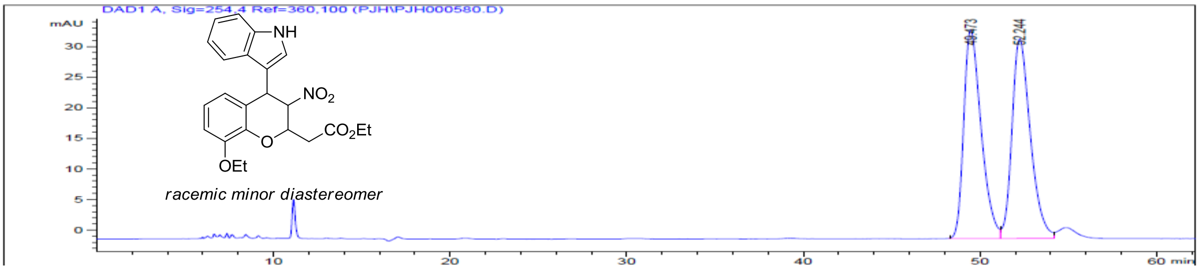

Peak #	RetTime [min]	Type	Width [min]	Area [mAU*s]	Height [mAU]	Area %
1	49.473	BV	1.0371	2334.13867	34.16302	49.7034
2	52.244	VB	1.0916	2361.99219	32.66733	50.2966

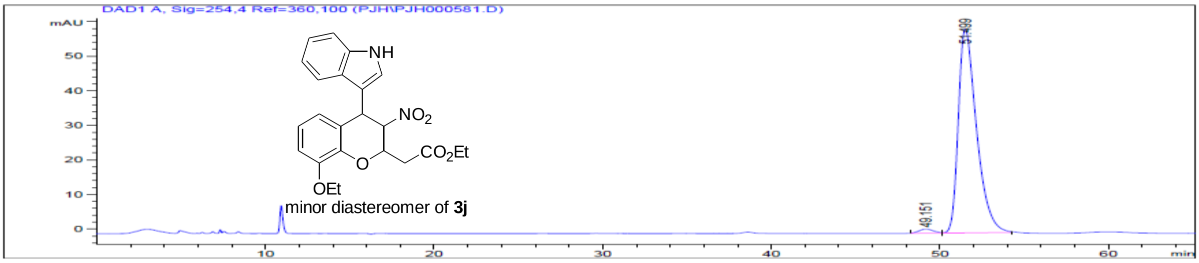

Peak #	RetTime [min]	Type	Width [min]	Area [mAU*s]	Height [mAU]	Area %
1	49.151	BV	0.7148	66.87046	1.14935	1.4918
2	51.499	VB	1.1292	4415.74072	58.99606	98.5082

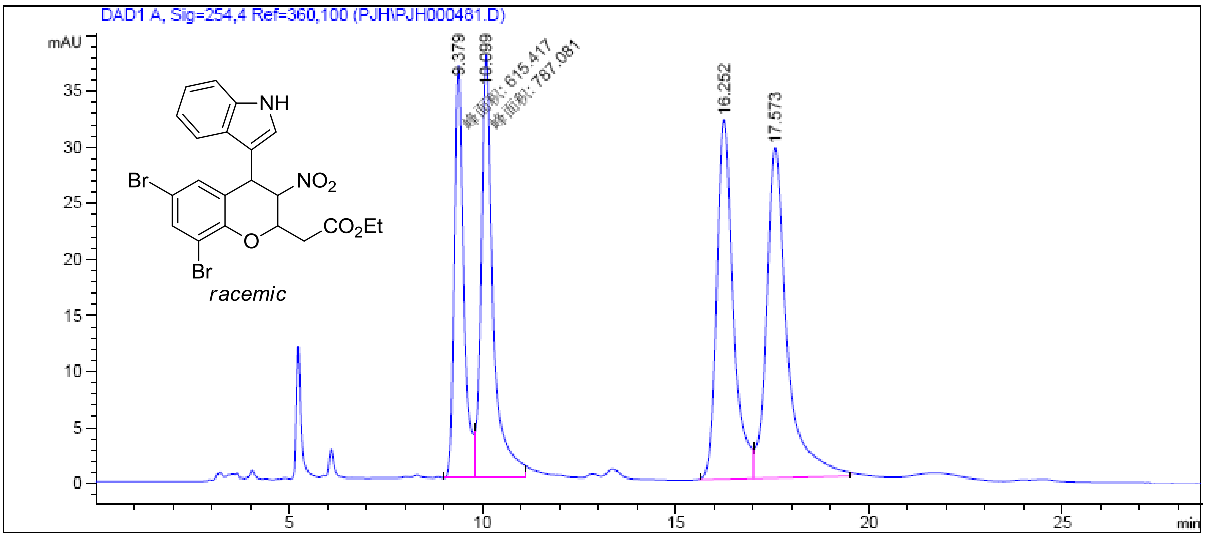

Peak #	RetTime [min]	Type	Width [min]	Area [mAU*s]	Height [mAU]	Area %
1	9.379	MM	0.2796	615.41724	36.67808	17.9115
2	10.099	MM	0.3484	787.08063	37.64749	22.9077
3	16.252	BV	0.4450	948.49451	32.07032	27.6056
4	17.573	VB	0.5379	1084.88025	29.44399	31.5751

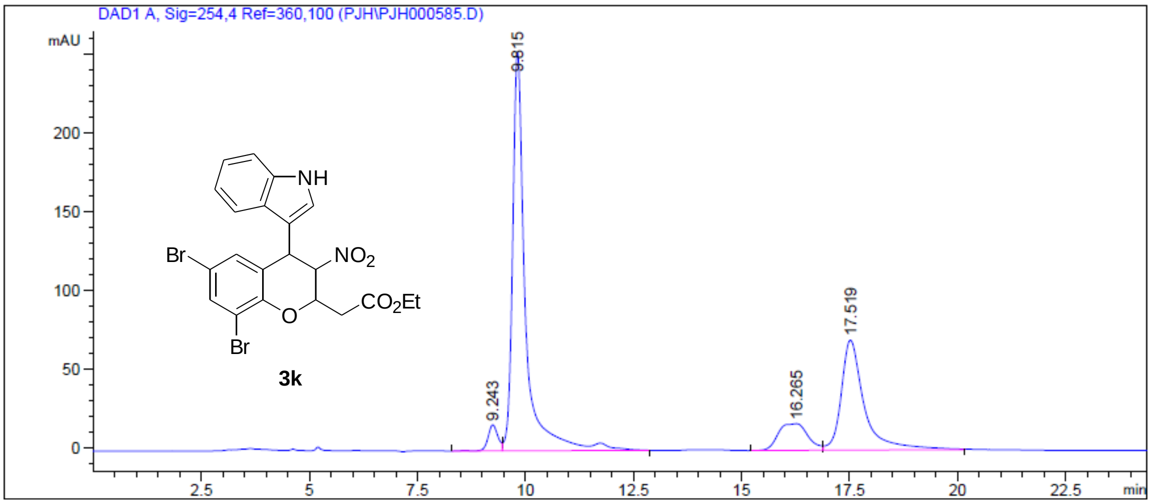

Peak #	RetTime [min]	Type	Width [min]	Area [mAU*s]	Height [mAU]	Area %
1	9.243	BV	0.2447	267.49939	16.53724	3.0027
2	9.815	VB S	0.2999	5246.45068	253.04375	58.8910
3	16.265	VV	0.6382	803.31738	16.91024	9.0172
4	17.519	VB	0.5455	2591.48291	69.75050	29.0892
